# Characterizing nonnative plants in wetlands across the conterminous United States

**DOI:** 10.1007/s10661-019-7317-3

**Published:** 2019-06-20

**Authors:** Teresa K. Magee, Karen A. Blocksom, Alan T. Herlihy, Amanda M. Nahlik

**Affiliations:** 1 Office Research and Development, National Health and Environmental Effects Laboratory, Western Ecology Division, US Environmental Protection Agency, 200 SW 35th Street, Corvallis, OR 97333 USA; 2 Department of Fisheries and Wildlife, Oregon State University, Corvallis, OR USA

**Keywords:** Nonnative plant indicator (NNPI), Potential ecological stress, Nonnative richness, Nonnative relative cover, Nonnative relative frequency, National Wetland Condition Assessment (NWCA), Conterminous United States

## Abstract

Nonnative plants are widely recognized as stressors to wetlands and other ecosystems. They may compete with native plant species or communities and alter ecosystem properties, which can affect ecological condition, posing challenges to resource managers. As part of the United States Environmental Protection Agency’s National Wetland Condition Assessment (NWCA), we characterized the status of nonnative plants in wetlands across the conterminous United States (US). Our primary goals were to (1) document the composition of nonnative taxa at 1138 NWCA sites sampled in 2011 and (2) estimate the areal extent of wetland under stress from nonnative plants within the NWCA 2011 sampled population of ~ 25 million ha of wetland (represented by 967 sampled probability sites and the NWCA survey design). A total of 443 unique nonnative taxa were observed, encompassing a species pool adapted to diverse ecological conditions. For individual sites, the number of nonnative taxa ranged from 0 to 29, and total absolute cover of nonnatives ranged from 0 to 160%. We devised the nonnative plant indicator (NNPI) as a categorical indicator of stress (low to very high) from the collective set of nonnative plant taxa occurring at a particular location, based on a decision matrix of exceedance values for nonnative richness, relative frequency, and relative cover. Wetland area of the sampled population occurring in each NNPI category was estimated at the scale of the conterminous US and within five large ecoregions and four broad wetland types. Potential stress from nonnative plants, as indicated by the NNPI category, was low for approximately 61% (~ 15.3 million ha), moderate for about 20% (~ 5.2 million ha), high for about 10% (~ 2.48 million ha), and very high for about 9% (~ 2.2 million ha) of the wetland area in the entire sampled population. Percent of wetland area with high and very high NNPI varied by ecoregional subpopulations: greater within interior and western ecoregions (~ 29 to 87%) than within ecoregions in the eastern half of the nation (~ 11%). Among wetland type subpopulations, greater percent of wetland area with high and very high NNPI was observed for herbaceous vs. woody types and for inland vs. estuarine types. Estimates of wetland area by NNPI categories are expected to be useful to policy makers or resource managers for prioritizing management actions by identifying situations where stress from nonnative plants is most extensive. We also considered four exploratory analyses aimed at providing ecological information useful in interpreting NNPI extent results. We conducted three population-scale analyses examining ecoregional and wetland type population means for (1) the three NNPI metrics, (2) absolute cover of growth-habit groups of nonnative plants, and (3) metrics describing human-mediated disturbance. Finally, we examined ecological relationships with site-level NNPI status using a random forest (RF) analysis with NNPI as the response variable and predictor variables including ecoregion, wetland type, and a variety of characteristics describing natural vegetation structure, environment, and human-mediated disturbance.

**Electronic supplementary material:**

The online version of this article (10.1007/s10661-019-7317-3) contains supplementary material, which is available to authorized users.

## Introduction

Nonnative plants are recognized as important indicators of stress to wetlands and other ecosystems (Mack and Kentula 2010; Magee et al. 2010; Schweiger et al. 2016). Their presence, richness, and abundance are often positively related to stressors of ecological condition caused by human-mediated disturbances (e.g., physical disturbances to vegetation or ground surface, changes in hydrology, nutrient inputs, changes in surrounding land use, and inadvertent introduction) (Lozon and MacIsaac 1997; Magee et al. 1999; Mack et al. 2000; Magee and Kentula 2005; Aguiar et al. 2007; Ringold et al. 2008; Jakubowski et al. 2014). Nonnative plants can also be direct stressors to ecological condition, altering native plant communities or ecosystem structure and processes (Vitousek et al. 1997; Dukes and Mooney 2004; Ehrenfeld 2010; D’Antonio and Flory 2017) and leading to losses of ecosystem services (Dukes and Mooney 2004; Hooper et al. 2005; Meyerson and Mooney 2007). Ecological impacts from nonnative species reflect changes to the biota, physical habitat, or processes of an ecosystem, with impact varying in type, direction, magnitude, and over space or time (Ricciardi et al. 2013). Reviews and meta-analysis from the literature indicate that nonnative plant species with potential for significant direct and indirect effects to the structure and function of ecosystems are common, and many of these taxa likely have yet to be recognized as harmful (Simberloff 2011; Vilà et al. 2011). In addition, ecological impacts from nonnative species can occur at the levels of organism (e.g., fitness, mortality, growth), species population (e.g., abundance, genetics), plant community (e.g., species richness, species composition, structure), ecosystem (e.g., physical habitat, nutrient cycling), or region (Simberloff 2011; Vilà et al. 2011; Ricciardi et al. 2013).

For example, nonnative plants have been linked to (1) increased risk of local extinction or population declines for many rare, native plant species (Randall 1996; Lesica 1997; Seabloom et al. 2006; Gilbert and Levine 2013); (2) changes in species composition and vegetation structure within and among plant community types, and homogenization of local and regional floras (McKinney 2004; Rooney et al. 2004; Magee et al. 2008); (3) alteration of fire regimes (Dwire and Kauffman 2003; Brooks et al. 2004); (4) alteration of geomorphic and hydrologic processes (Rowantree 1991; Sala et al. 1996; Gebauer et al. 2016); and (5) alteration of carbon storage patterns (Farnsworth and Meyerson 2003; Bradley et al. 2006), nutrient cycling, and soil biota including microbial and mycorrhizal interactions with plants (Belnap and Phillips 2001; Ehrenfeld et al. 2001; Ehrenfeld 2003; Bowen et al. 2017). In addition, impact from nonnative plants to natural ecosystems may be exacerbated by ongoing global changes, with effects varying by region, ecosystem type, plant community type, and type of modification (e.g., climate, land use, and nutrient dynamics) (Dukes and Mooney 1999; Dwire et al. 2017; Liu et al. 2017).

As part of the 2011 National Wetland Condition Assessment (NWCA) (USEPA 2016c; Kentula and Paulsen 2019), conducted by the United States Environmental Protection Agency (USEPA) and its partners, we devised the nonnative plant indicator (NNPI) as an indicator of ecological stress to wetlands (USEPA 2016d). The vegetation data collected during the 2011 NWCA provide an unprecedented opportunity for characterizing nonnative plants in wetlands across the conterminous United States (US). In addition, the field protocols (USEPA 2011a) and the probability design (Olsen et al. 2019) underpinning the NWCA allow for detailed analyses across sampled sites, or, alternatively, for the expression of results as estimates of wetland area (i.e., extent estimates) within a sampled population, at national or regional scales (e.g., this paper, Lomnicky et al. 2019; Magee et al. 2019; Nahlik et al. 2019).

Directly measuring impacts to ecological condition is often not possible because the tools to do so are lacking or are prohibitive in cost and time, especially for large-scale studies. Consequently, stressor indicators have been widely used in large-scale ecological assessments and are typically based on straightforward, easy-to-measure factors that reflect anthropogenic-driven properties related to declining ecological condition, but not necessarily implying direct or causal mechanisms for this decline (e.g., USEPA 2009; Mack and Kentula 2010; Sifneos et al. 2010; USEPA 2016a, b, d; Lomnicky et al. 2019). Similarly, the NNPI is not intended as a direct measure of ecological impact from nonnative plants. Rather, it is a categorical indicator based on a decision matrix, which considers all nonnative plants occurring at a given location using values for three metrics (richness, relative frequency, and relative cover of nonnative plant taxa) that each reflect different pathways of potential ecological impact (USEPA 2016d). In this paper, we give an overview of the NNPI, including the rationale for (1) considering all nonnatives occurring at a sampled location, (2) the selection of the three NNPI metrics, and (3) the assignment of metric-specific exceedance values for designating four categories (low to very high) of potential stress (hereafter, stressor-level categories).

Following the overview of the NNPI, the remainder of our paper focuses on the characterization of nonnative plant species in wetlands across the conterminous US using data from the 2011 NWCA. We, first, briefly characterize the study area by (1) defining the NWCA sampled population and reporting on estimated wetland area within ecoregional and wetland type subpopulations and (2) describing a variety of ecological attributes for each of these subpopulations. Second, we provide a summary of the individual nonnative species observed across sites sampled in 2011 to illustrate the scope of nonnative taxonomic diversity and abundance. Next, for the 2011 NWCA sampled population, we use the NNPI to estimate the area of wetland that falls into each of the NNPI stressor-level categories (low, moderate, high, very high) at the scale of the conterminous US and within major ecoregions and wetland types. Wetland area estimates within the different NNPI categories are expected to be useful to policy makers or resource managers in identifying situations where impact from nonnative plants is most extensive, and in informing and prioritizing management actions and future research. Finally, to aid in understanding the patterns described by the NNPI extent results, we also conduct and discuss a series of *exploratory* analyses. We consider how (1) the individual NNPI metrics, (2) growth-habit groups of nonnative plants, and (3) human-mediated disturbances might parallel population-scale ecoregional and wetland type patterns for NNPI extent results. We also explore how ecosystem and disturbance characteristics relate to site-scale NNPI stressor-level. These exploratory analyses help identify relationships that can be useful for hypothesis generation to inform future research.

## Description of the nonnative plant indicator

### Rationale

Stressor indicators for a large-scale study like the NWCA should be widely applicable across major ecoregions and wetland types. Nonnative plant species meet this criterion because they are not natural components in any ecosystem, are often associated with human-mediated disturbance, and, in many cases, are known to directly impact ecological condition. In addition, the identity and abundance of individual nonnative species or groups of nonnative plants can be readily described using species composition data that are commonly collected in field studies. Many nonnative taxa are known to be particularly invasive or associated with negative ecological impacts in a wide range of plant communities and environments (Randall et al. 2008; Magee et al. 2010; Barney et al. 2013; Blackburn et al. 2014). Rather than focusing only on those nonnative taxa currently documented as highly invasive or impactful, we chose an inclusive approach and based the NNPI on the entire complement of nonnative plants co-occurring at a given location. We did this to retain all useful signal in the nonnative plant data, because consideration of all nonnative species occurring together encompasses a variety of important ecological consequences.

For example, the type and level of impact from nonnatives is often dependent on species traits of invaders, traits of the recipient plant community, and the environmental context (e.g., habitat, biome or region, level of human-mediated disturbance) being invaded (Richardson and Pyšek 2006; Pyšek et al. 2012; Ricciardi et al. 2013). Individual nonnative plant taxa characterized by rapid spread across the landscape or dominance where they occur, or that act as ecosystem engineers (i.e., influence resource availability by altering biotic or abiotic elements of ecosystems), are likely to cause more immediate disruption to ecological condition than infrequent, low cover, or recently naturalized taxa (Richardson and Pyšek 2006; Baiser et al. 2008; Ehrenfeld 2010; Ricciardi et al. 2013). However, the sheer numbers of individual infrequent or low cover nonnative species that occur in natural landscapes (e.g., Seabloom et al. 2006; Pyšek et al. 2017) represent a likely invasion debt (i.e., a delay between introduction and extensive spread (Seabloom et al. 2006; Bennett et al. 2013; Beauvais et al. 2016)); that is, an undetermined subset of such nonnative taxa can be expected to undergo significant expansion in cover and distribution as they come into equilibrium with their introduced ranges, or as shifts in environmental conditions accompanying global change occur (Seabloom et al. 2006; Bennett et al. 2013). In addition, co-occurring multiple invaders can interact with one another and with native species via direct or indirect facilitative, neutral, or competitive pathways that may alter community composition and environmental conditions; potentially leading to nonnative accumulation or invasional meltdown, i.e., acceleration of nonnative establishment or ecological impact resulting in unrecoverable replacement of native communities (Simberloff 2006; Kuebbing et al. 2013; Kuebbing and Nuñez 2016). Taken together, the varied and interactive ecological effects of nonnative plants make it difficult to predict which nonnative species are likely to have the greatest impact in specific environments or plant communities.

In addition to incorporating all nonnative taxa co-occurring at a given location, we based the categorical NNPI on straightforward metrics that can be readily calculated from field observations to allow maximum applicability and ease of use. We selected three complementary metrics for use in the NNPI: (1) relative cover of nonnative species, (2) nonnative species richness, and (3) relative frequency of nonnative species. Formulas for their calculation are provided in Table 1. All three metrics consider all nonnative species at a location and each metric describes different possible avenues to ecological stress. Relative nonnative cover (0 to 100%) reflects preemption of space and resources and is often associated with changes in plant community composition (species identity, richness, and abundance) and vegetation structure (horizontal or vertical), or with alteration of ecosystem processes (e.g., hydrology, nutrient cycling, fire regime). Greater nonnative richness (number of unique nonnative species) increases the risk that individual nonnative taxa are or may become invasive, or act as ecosystem engineers that negatively alter biotic or abiotic properties. Increasing relative nonnative frequency (0 to 100%) across a site reflects increasing numbers of foci from which nonnatives could compete with native species, expand in cover, or spread to new locations.

**Table 1:** Nonnative plant species metrics used in the nonnative plant indicator (NNPI)

Metric name	Calculation^a^
Nonnative relative cover	(∑ Absolute cover nonnative species_*i*_/∑ Absolute cover all species_*i*_) × 100; where for each unique species *i*: Absolute Cover = 0–100%
Nonnative richness	Number of unique nonnative species
Nonnative relative frequency	∑ Frequency nonnative species_*i*_/∑ Frequency all species_*i*_) × 100; where for each unique species *i*: Frequency = 0–100%, calculated as the percent of Veg Plots in which it occurred.

^a^Calculation of metrics based on data collected in the five 100-m^2^ vegetation plots at each site (vegetation data collection described in the section “Plant data collection and trait assignment” under “Methods”)

Relative frequency and relative cover of nonnatives at each site were selected as NNPI metrics, rather than absolute frequency and cover (i.e., the sum of frequencies or cover values across all individual nonnative species occurring at a location). Relative values normalize these two metrics to reflect the proportional influence of nonnatives within the varying vertical structure and species diversity represented by different vegetation types. For example, forested systems have more vertical layers than herbaceous systems (e.g., bottomland hardwood forest vs. seasonal wet prairie) and, therefore, greater total species cover. Likewise, some community types have greater species richness than others (e.g., mountain fen vs. *Spartina alterniflora* salt marsh) and, consequently, greater frequency of species occurrences at a site. In addition, some community types have varying amounts of nonvegetated area, such as bare ground or standing water, as part of their ecosystems. Thus, the same absolute (or total values) for nonnative cover or frequency could reflect very different proportions of the total vegetated component of the ecosystem under consideration.

The NNPI metrics (Table 1) were calculated using R code developed for the NWCA, and site values for each of the three metrics are included in the 2011 NWCA vegetation metric data (USEPA 2016m). See the “Plant data collection and trait assignment” section under “Methods” of this paper for description of the sampling and information gathering procedures for the data used in calculating the NNPI metrics. We evaluated signal:noise (S:N) for each of the NNPI metrics to ensure that, in addition to being ecologically appropriate, they would be effective at detecting signal in the data (USEPA 2016d). S:N is the ratio of variance in a metric across all sampled sites (signal) to the variance in the metric based on repeat sampling of individual sites (noise) (Kaufmann et al. 1999). Ninety-six probability sites, two per state, were identified in the survey design for repeat sampling during the field season (revisit sites) to gauge within sampling period data variability (noise). Metrics having S:N values greater than 2 are considered useful in making ecological distinctions (Stoddard et al. 2008; Magee et al. 2019). S:N exceeded this value for all the NNPI metrics: 5.9 for nonnative species richness, 23.9 for relative frequency of nonnative species, and 14.3 for relative cover of nonnative species.

### NNPI stressor-level categories

The three NNPI metrics (nonnative relative cover, nonnative richness, and nonnative relative frequency, Table 1) were used together in a decision matrix to assign each sampled site to a stressor-level category (low, moderate, high, or very high) based on exceedance values (Table 2) for each metric. The NNPI status for each site was determined by the highest stressor-level category observed across the three NNPI metrics. This *filter approach* of using multiple metrics to designate the indicator status for individual sites was developed by Herlihy and others (e.g., Herlihy et al. 2008; Herlihy et al. 2019a), and we adapted this approach for the NNPI (USEPA 2016d). No established procedure existed to define boundaries for the stressor-level categories based on specific values of the NNPI metrics. Consequently, as a starting point, we defined exceedance values for the four stressor-level categories for each metric (Table 2) using best professional judgment based on our broad experience with numerous wetland community types and our perceptions of change in plant community composition and structure accompanying varying levels of nonnative cover, frequency, or richness. Major changes in plant community composition and structure are also often associated with impact to other biota and ecosystem properties (e.g., see literature cited in the “Introduction” and the “Rationale” sections). Exceedance values for the four stressor-levels were assigned to reflect the strong potential influence of nonnative relative cover, with the values for nonnative richness and nonnative relative frequency set to reflect these two metrics as additional sources of ecological stress. We recognize that other researchers might select different exceedance values for the four stressor-level categories; nevertheless, the exceedance values assigned for the three NNPI metrics were received favorably by NWCA partners (wetland scientists and managers) during discussions at analysis workshops and during extensive peer review of the NWCA Technical Report (USEPA 2016d).

**Table 2:** Stressor-level category exceedance thresholds for each of the three metrics informing the nonnative plant indicator (NNPI)

Stressor-level category*	Relative cover nonnative species	Richness nonnative species	Relative frequency nonnative species
Low	≤ 1	≤ 5	≤ 10
Moderate	> 1–15	> 5–10	> 10–30
High	> 15–40	> 10–15	> 30–60
Very high	> 40	> 15	> 60

*Exceedance of a threshold value for a particular stressor-level category for any of the three component metrics moves the NNPI to the next higher stress level

As an example of how the exceedance values for the nonnative metrics (Table 2) are utilized, consider two hypothetical sites. *Hypothetical site 1* has nonnative relative cover of 7%, placing the site in the moderate stressor-level category. However, this site also has nonnative richness of 14 species and relative frequency of 32%, which reflect the high stressor-level for both metrics; thus, the site would be assigned to the high NNPI category. Even though relative nonnative cover is not extensive at this hypothetical site, the number of individual nonnative species and their frequency of occurrence might indicate shifting community composition and strong risk for expansion of nonnative cover. Next, consider *hypothetical site 2* with 80% nonnative relative cover that places the site in the very high stressor-level category, nonnative richness of 1 indicating the low stressor-level category, and nonnative relative frequency of 59% that indicates high stressor-level. Here, the hypothetical site NNPI would fall into the very high category. Even though there is only one nonnative species present at the site, it occupies 80% of the total vegetation cover and nearly 60% of all species occurrences across the sampled area of the vegetation plots are nonnative.

## Methods

### Survey design and data use

The NWCA probability-based survey design is detailed in Olsen et al. (2019). Sites were identified using a spatially balanced generalized random tessellation stratified design for an area resource (Stevens Jr. and Olsen 1999, 2004) and the US Fish & Wildlife Service’s National Wetland Status and Trends digital sample frame for wetlands (Dahl and Bergeson 2009; Dahl 2011). The target wetland population for the NWCA encompassed tidal and nontidal systems, across the conterminous US, that had rooted vegetation and, when present, open water less than 1 m deep (Table 3). Each selected sample point (i.e., coordinates of site location) received a weight reflecting the wetland area within the target population represented by that point (Olsen et al. 2019). Sample weights for probability sites were used to (1) estimate wetland area with particular characteristics across the nation, regionally, or by wetland type; and (2) to calculate population-weighted means for specific vegetation attributes and environmental metrics (Diaz-Ramos et al. 1996). Area estimates and population means are calculated with known margins of error (two-sided 95% confidence interval (CI)) based on a local neighborhood variance estimate (Stevens Jr. and Olsen 2003). Site selection, weight assignment, wetland area estimation, and calculation of population-weighted means for specific metrics were completed using R statistical software (R_Core_Team 2015, 2017) and the ‘spsurvey’ R contributed package (Kincaid and Olsen Jr. 2015).

**Table 3:** Definition of NWCA target population and description of the NWCA aggregated wetland types used in analysis

Target population		NWCA aggregated wetland type	Description
Wetlands across conterminous US representing tidal and nontidal systems with rooted vegetation and, *when present, open water ≤ 1 m deep*	Estuarine intertidal	EH—estuarine intertidal herbaceous	Estuarine or intertidal emergent wetlands
		EW—estuarine intertidal woody	Estuarine or intertidal shrub and forested wetlands
	Inland	PRLH—palustrine, riverine, and lacustrine herbaceous	Emergent, ponded, or previously farmed wetlands in palustrine, shallow riverine, or shallow lacustrine littoral settings
		PRLW—palustrine, riverine, and lacustrine woody	Forest- or shrub-dominated wetlands in palustrine, shallow riverine, or shallow lacustrine littoral settings

**Fig. 1:**
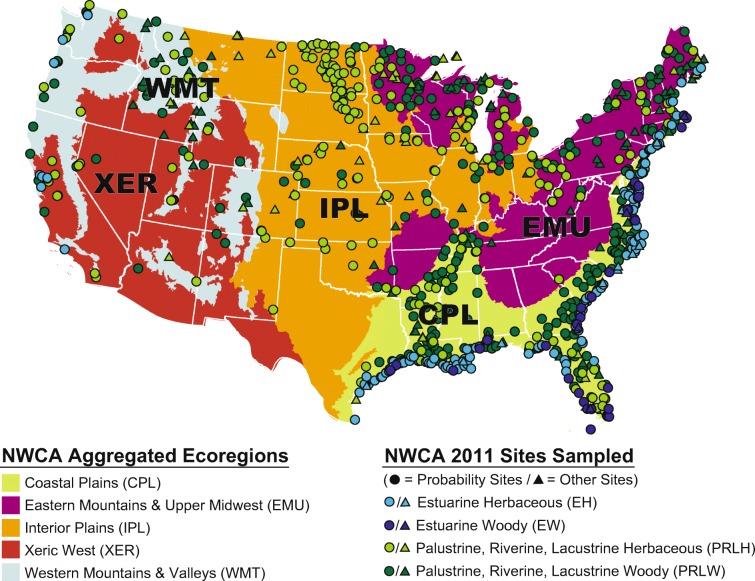
Locations of 1138 sites sampled in the 2011 National Wetland Condition Assessment (NWCA) and their distribution across five NWCA ecoregions (see Table 4). See Table 3 for definitions of the estuarine (EH and EW) and inland (PRLH and PRLW) wetland types

The inference (or sampled) population for the NWCA was described by 967 probability sites that were identified in the survey design and sampled in 2011 (Fig. 1, Table 4). Only data from these 967 probability sites were used to make wetland area estimates for the sampled wetland population or subpopulations and to calculate population-weighted means for specific metrics describing vegetation attributes or environmental conditions for the sampled population or subpopulations.

**Table 4:** Distribution of the 1138 sites sampled in the 2011 NWCA. ‘Other’ sites were selected outside NWCA design. ‘Probability’ sites are from survey design. ‘Wetland area’ is area and 95% CIs represented by the probability sites

NWCA population		Total sites	Other sites	Probability sites	Wetland area (10^6^ ha) in sampled population	% sampled population area
Conterminous US						
	All sites^a^	1138	171	967	25.15 ± 2.13	100
Ecoregion						
CPL	Coastal Plain	567	54	513	12.5 **±** 1.51	50
EMU	Eastern Mountains and Upper Midwest	214	62	152	8.08 ± 1.26	32
IPL	Interior Plains	190	34	156	3.10 ± 0.74	12
XER	Xeric West	62	3	59	1.08 ± 0.31	4
WMT	Western Mountains and Valleys	105	18	87	0.40 ± 0.11	2
Wetland type						
EH	Estuarine intertidal herbaceous	272	14	258	2.01 ± 0.42	8
EW	Estuarine intertidal woody	73	4	69	0.20 ± 0.13	1
PRLH	Inland herbaceous	358	56	302	5.70 ± 0.81	23
PRLW	Inland woody	435	97	338	17.43 ± 1.91	69

^a^Totals across subpopulations under ecoregions and wetland type equal the all sites totals. See Fig. 1 for NWCA ecoregion boundaries and Table 3 for aggregated wetland type definitions

An additional 171 sites were selected outside of the NWCA survey design (Fig. 1) and were used only in two analyses that did not involve extrapolation of results across the areal extent of the NWCA sampled population. This set of ‘other’ sites included 21 that supported state-level studies that were part of the NWCA, but could be used only for site-level analyses because their locations were selected outside the NWCA probability design (USEPA 2016d). The remaining 150 ‘other’ sites were handpicked in an effort to represent locations with limited anthropogenic disturbance; however, these sites actually spanned a range of disturbance levels with only about half meeting criteria for least-disturbed reference sites (Herlihy et al. 2019a). We used data from all 1138 sampled sites (probability + other sites) to maximize sample sizes for two site-scale analyses: (1) summarizing the occurrence and abundance of all individual nonnative species observed and (2) examining site-scale relationships of ecological predictors to NNPI stressor-level categories. Sample weights could not be used when all 1138 sites were considered; thus, results of these two analyses reflect site-specific patterns that should not be inferred across the entire sampled population.

All 1138 sampled sites were classified into five aggregated ecoregions (Fig. 1, hereafter ecoregions) and four broad aggregated wetland types (Table 3, Fig. 1; hereafter, wetland types) to minimize within-group variation and still maintain sufficient sample sizes for analysis (see, Herlihy et al. 2019a; Magee et al. 2019). Table 2 lists the distribution of the 1138 sites sampled across the conterminous US, by ecoregion and wetland type. Ecoregion and wetland type designations and a variety of NWCA site descriptors (e.g., site identifiers, location, sample weights) used to support analyses are included in NWCA site information data (USEPA 2016l).

### Plant data collection and trait assignment

Field and laboratory methods for collecting data on vegetation for the 2011 NWCA are outlined here and detailed elsewhere (USEPA 2011a, b; Magee et al. 2019). Vegetation sampling methods were widely reviewed, tested, and vetted during the development of the NWCA sampling protocols described in the NWCA Field Operations Manual (USEPA 2011a). The 53 NWCA field crews received intensive training in sampling protocols prior to the field season and had access to expert and logistic support throughout the field season (McCauley et al. 2019). Each field crew included a two-member vegetation team, at least one of whom had strong expertise in the flora of the state or region where the crew worked. Plant species data were collected during the peak growing season (determined regionally as the time period when most plants were in flower or fruit) to optimize species identification and characterization of species abundance. At each site, plant data were gathered from five 100-m^2^ Vegetation (Veg) Plots that were systematically placed within a typically circular, 0.5 ha assessment area (AA). Alternate configurations for AA shape and other systematic Veg Plot layouts were used only when necessary, as determined by rules related to specific site conditions (USEPA 2011a).

All vascular plant taxa occurring in each Veg Plot were identified to the lowest taxonomic level possible (typically species or lower, but occasionally genus or family). Specimens were collected for plant taxa that could not be identified in the field, and later identified in the lab by regionally expert botanists. Percent absolute cover for each taxon (0 to 100%) in each Veg Plot was visually estimated across the entire 100-m^2^ area (USEPA 2011a), and cover data for the observed taxa can be found in USEPA (2016i). Taxonomy for all observed vascular plant taxa was standardized (USEPA 2016d) to PLANTS Database nomenclature (USDA-NRCS 2014).

State-level native status categories (USEPA 2016d; Magee et al. 2019) were designated for the nearly 13,000 taxon–state pairs (trait data: USEPA 2016h) observed across the 1138 NWCA sites sampled across the conterminous US. A body of evidence approach was used to identify the state-level native status of each observed taxon. Native status categories (Table 5 and *listed in italics below*) for the taxon–state pairs were assigned based on review of numerous taxonomic and ecological sources (*n* ~ 85), including state and regional floras and checklists, and state and national floristic databases and distribution maps (USEPA 2016d). For species with complex origins or species for which limited information was available, consultation with the PLANTS Database (USDA-NRCS 2013) nomenclatural team helped inform native status determinations. Taxa recognized as indigenous to a certain state were identified as *Native*. *Alien* plants were designated as those that were either (1) *Introduced* to the conterminous US, or (2) *Adventive*, that is, native to some parts of the conterminous US but introduced to the location of occurrence at a particular NWCA site. *Cryptogenic* species (Carlton 1996; Galatowitsch et al. 1999) include taxa with both introduced (often aggressive) and native (generally less prevalent) genotypes, varieties, or subspecies. Many cryptogenic taxa found in wetlands have strongly invasive components (e.g., *Phalaris arundinacea* (Brodersen et al. 2008; Jakubowski et al. 2014), *Phragmites australis* (Simberloff et al. 2012; Allen et al. 2017a, b; Bowen et al. 2017)). Thus, we grouped cryptogenic species with alien taxa as *Nonnatives* (Table 5) for purposes of our analysis. Taxa identified only to growth habit or family, or to genera with native and alien species, were given a native status of *Undetermined*.

**Table 5:** Definition of state-level native status designations for NWCA taxa

Native status designations	
*Native:* indigenous to specific states in the conterminous US	
*Nonnative*: alien + cryptogenic	
*Alien:* introduced + adventive	
*Introduced:* indigenous outside of, and not native in, the conterminous US	
*Adventive:* native to some areas of the US, but introduced in the location of occurrence	
*Cryptogenic:* both native and introduced genotypes, varieties, or subspecies *Undetermined*: growth habits, families, genera with native and alien species	

Species growth-habit designations were obtained from the PLANTS database (USDA-NRCS 2012) and summarized (USEPA 2016d) to assign standardized growth habit to the individual vascular taxa observed in the NWCA (trait data: USEPA 2016j). These standardized growth-habit categories were further consolidated to forb, graminoid, vine, shrub, and tree. Regionally specific wetland indicator status (OBL—obligate, FACW—facultative wetland, FAC—facultative, or FACU—facultative upland) for each observed NWCA species (trait data: USEPA 2016k) was based on the National Wetland Plant List (NWPL) and associated Wetland Regions defined by US Army Corps of Engineers (USACE 2014). Upland (UPL) status was assigned to NWCA taxon-region pairs not listed in the NWPL. In addition, a numeric value was assigned to each wetland indicator status (i.e., OBL = 1, FACW = 2, FAC = 3, FACU = 4, and UPL = 5).

### Characterization of the 2011 NWCA sampled population

The extent of the NWCA sampled population was characterized using sample weights for the individual sampled probability sites (*n* = 967) to estimate the area by wetland type within ecoregions. In addition, population-weighted means (± 95% CI) were calculated for several native vegetation and environmental attributes, using values observed at the 967 sampled probability sites and the site sample weights, to provide a general description of ecoregion and wetland type subpopulations. Attributes of native vegetation included native species richness (number of unique native species) and the absolute percent cover for native plants by growth habit (forb, graminoid, shrub, tree, and vine). Absolute cover was used to represent the area at each sampled site influenced by each growth-habit group. A wetland index (WI) based on species composition was calculated for each site. The WI is a cover-weighted wetland affinity score based on all plant species observed at a site: the sum of the numeric value (1 to 5) representing wetland indicator status (OBL to UPL) for each species weighted by its absolute cover, divided by the sum of absolute cover values for all observed species (Wentworth et al. 1988). A WI with a value of 1 indicates entirely obligate wetland vegetation and a value of 5 indicates entirely upland vegetation (Wentworth et al. 1988). Because the WI is correlated with moisture gradients, it provides a rough description of relative hydric conditions (Wentworth et al. 1988; Schweiger et al. 2016). Native vegetation attributes and the wetland index were based on data collected in the Veg Plots at each sampled site and are included in or calculated from the 2011 NWCA vegetation metric database (USEPA 2016d, m). Environmental characteristics included percent cover of bareground observed across the Veg Plots at each sampled site (USEPA 2016m), mean annual precipitation (30-year average within 1000-m radius surrounding the AA center for each site) from the PRISM database (Daly et al. 2008; PRISM Climate Group 2013), and maximum elevation (within 200-m radius surrounding the AA center) based on NHDPlusV2 NEDSnapshot (USGS 2006; McKay et al. 2012). Precipitation and elevation metric values for NWCA sites are available from the NWCA land use data set (USEPA 2016g).

### Characterization of the complement of observed nonnative plant taxa

We examined the distribution of the 443 unique nonnative taxa observed across all NWCA sampled sites (*n* = 1138) to document the scope of nonnative taxonomic diversity and abundance patterns. To do this, we constructed an ordered table (Supplement 1) listing each nonnative species by (1) growth-habit group (forb, graminoid, vine, and shrub/tree), (2) total number of site occurrences, (3) mean importance ((% frequency + % cover)/2) at sites of occurrence, and (4) number of site occurrences in each of the five NWCA ecoregions.

### Estimates of wetland area by NNPI stressor-level

The NNPI status and the sample weight from the 2011 NWCA survey for each sampled probability site (*n* = 967) were used to estimate the wetland area with low, moderate, high, or very high stressor-level categories for the NWCA sampled population. Area estimates with 95% CIs for NNPI stressor-level categories were made nationally, by wetland type, and by ecoregion.

### Exploratory analyses

#### Population means for NNPI metrics and growth-habit groups for nonnatives

Population-weighted means (± 95% CI), based on observed values at the 967 probability sites and the site sample weights, were calculated for the three NNPI metrics (Table 1: nonnative richness, nonnative relative frequency, and nonnative relative cover) for ecoregion and wetland type subpopulations. We also examined population-weighted means for absolute percent cover of nonnatives within four growth-habit types (forb, graminoid, vine, trees/shrubs) at the scale of the conterminous US, and for ecoregion and wetland type subpopulations to evaluate how nonnative abundance might vary by growth habit. Nonnative trees and shrubs were combined into one group because many individual nonnative woody species occur in both shrub and tree growth habits, and because mean cover values for nonnatives classed only as shrubs were often small. For the growth-habit metrics, we examined absolute percent cover, rather than relative cover, to distinguish the spatial area influenced by each growth-habit group at each sampled site. Differences in population-weighted means between different variables were characterized based on nonoverlapping 95% CIs.

#### Characterization of human-mediated disturbance

We evaluated population-weighted means (± 95% CI), calculated using the observed values at the 967 probability sites and the site sample weights, for three attributes of human-mediated disturbance for ecoregions and wetland types. Indicators of human-mediated disturbance included a site-scale index and two landscape metrics (percent agricultural and percent developed land use). The site-level disturbance index summarizes the overall human-mediated disturbance observed on-the-ground at each site at the date of sampling, and was based on combination of eight indices, which describe several disturbance types defined in USEPA (2016d). These eight disturbance indices were derived from approximately 85 disturbance descriptors that were evaluated at each sampled site within the AA and its associated 100-m radius buffer. Five of these indices summarized categories of physical disturbances (agriculture, residential/urban, industrial, hydrologic, and habitat modifications) in the AA and buffer area, two described the level of hydrologic alterations in the AA (USEPA 2016d), and one described heavy metal concentrations in the soil of the AA (Nahlik et al. 2019). Data for these eight indices are available in USEPA (2016f). To create the overall site-level disturbance index, we standardized values for each specific disturbance index across all sampled sites to a 0 to 10 continuous scale using the formula: ((observed value − minimum)/(maximum − minimum) × 10), then summed these scores and multiplied this total by 10/8 to obtain an overall site-level disturbance index value with a possible range from 0 to 100. Percent agricultural and developed land use coverages (within a 1000-m radius surrounding the AA center) were based on the 2006 National Land Cover Database (Fry et al. 2011) and are available from the NWCA land use data set (USEPA 2016g).

#### Ecological relationships to site-level NNPI status

Random forest classification (RF) analysis (Liaw and Wiener 2002; Liaw and Wiener 2015) was used (1) to determine if the vegetation, environmental, and human-mediated disturbance metrics described in the previous sections, along with ecoregion and wetland type, might usefully predict NNPI stressor-level category; and (2) to examine the relative importance of each predictor variable in the resulting model. Complex ecological processes often involve multiple interactions and nonlinear relationships among variables, and RF analysis performs well with data that reflect these properties (De’ath and Fabricius 2000; Cutler et al. 2007; Fox et al. 2017). RF reduces model variance and increases prediction accuracy by building numerous decisions trees from bootstrap samples of a data set and averaging the predictions made by each tree in the forest (Cutler et al. 2007; Fox et al. 2017). In addition, RF is robust to the inclusion of variables with low importance (Fox et al. 2017), and variable importance is distributed across all the predictor variables in RF models, preventing elimination of ecologically important predictors of the response that might be correlated with other predictors (Cutler et al. 2007; Fox et al. 2017). We constructed our RF model for predicting NNPI status using the R computing language (R_Core_Team 2015, 2017) and the ‘randomForest’ package ver. 4.6-12 (Liaw and Wiener 2002, 2015) with the following options: (1) number of trees used to build the model (ntree) set to 1000 and (2) number of variables randomly selected at each tree node (mtry) set to the package default mtry = $\sqrt{p}$, where *p* is the number of predictor variables. NWCA sample weights were not considered in the analysis.

Although the NNPI is normally categorized into four stressor-levels (low, moderate, high, very high), for the RF analysis, these were aggregated into two combined stressor-levels (low–moderate vs. high–very high). Thus, the response for the RF classification is the categorization of all NWCA sampled wetland sites (*n* = 1138, probability + other sites) as having either low–moderate or high–very high NNPI stressor-level. We elected to use these combined stressor levels because some wetland type or ecoregion subpopulations had comparatively few sampled sites with high or very high NNPI. Also, preliminary RF analysis results showed greater percent correct classification of sites for the two-combined vs. four separate NNPI stressor-levels. Because the response data were unbalanced, with 72% of sampled sites having LM (*n* = 824) and 28% of sampled sites with high–very high (*n* = 314) NNPI status, we used a downsampling approach recommended by Fox et al. (2017) to improve predictive accuracy of the minority class. Each tree in the forest was built by drawing a bootstrap sample with the same number of cases from the low–moderate and high–very high categories based on the size of the minority class (*n* = 314 sites).

Procedures included in ‘randomForest’ were used to assess model performance based on correct classification of the response for each site (Liaw and Wiener 2002, 2015), in our case, low–moderate vs. high–very high stressor-level. The randomForest package uses the portion of data not contained in the bootstrap sample for an individual tree (the out-of-bag (OOB) data), to predict the response of site *i* for each modeled tree in the forest where *i* is OOB and takes the majority vote as the predicted stressor-level and the proportion of high–very high votes as the OOB predicted probability for that site (Cutler et al. 2007; Fox et al. 2017). Measures of model performance computed using these OOB predictions are essentially cross-validated accuracy estimates (Cutler et al. 2007; Fox et al. 2017). Here, we consider the following performance measures: percent of all sites (*n* = 1138) correctly classified, percent of high–very high NNPI sites correctly classified, and percent of low–moderate NNPI sites correctly classified.

To determine which predictors were most important in identifying when the NNPI was likely to be high–very high, variable importance was calculated as mean decrease in accuracy, and the results were plotted to show relative importance of the variables (using the ‘varImpPlot’ function in the ‘randomForest’ package, Liaw and Wiener 2015). Mean decrease in accuracy is a permutation measure of variable importance calculated using only OOB data, and higher values indicate greater importance of a predictor variable to the classification (Cutler et al. 2007). We also generated partial dependence plots for each predictor variable in the RF model, using the ‘partialPlot’ function in the ‘randomForest’ package (Liaw and Wiener 2015), to examine the influence of individual predictors. Partial dependence plots depict the probability of the high–very high stressor-level for the NNPI as a function of a specific predictor variable after averaging out the effects of the other predictor variables in the model (Liaw and Wiener 2015).

## Results and discussion

First, we define and briefly characterize the NWCA sampled population to which results of this study apply. Second, the complement of individual nonnative plant taxa observed across sites sampled in 2011 is described to illustrate the scope of nonnative taxonomic diversity and abundance. Next, we estimate the wetland area in the 2011 NWCA sampled population that falls into each of the four NNPI stressor-levels across the conterminous US and within major ecoregions and wetland types. To aid in understanding the patterns described by the NNPI extent results, we conduct a series of four *exploratory* analyses. We evaluate how population-scale means for (1) the three individual NNPI metrics, (2) growth-habit groups of nonnative plants, and (3) human-mediated disturbances might parallel NNPI stressor-level extent results. The final exploratory analysis uses RF classification to examine relationships of potentially interacting ecological attributes and disturbance characteristics to site-level NNPI status.

Most of the analyses presented in this paper are based on the 967 probability sites and use sample weights to reflect population-scale patterns (see “Methods” for details). All figures and tables reporting population-scale results include 95% CIs. Differences in wetland area, or in metric or attribute means, are recognized based on nonoverlapping CIs (USEPA 2016c, d). Although nonoverlapping CIs provide quantification of the level of confidence in the difference for a certain comparison, they do not necessarily equate to significant difference because multiple individual comparisons are considered in these analyses. Note, confidence intervals will tend to be larger where sample sizes are smaller (USEPA 2016c).

For the characterization of the complement of individual nonnative taxa observed in 2011 and the exploratory RF analysis, sample weights were not used because these analyses considered all sampled sites (*n* = 1138), rather than only probability sites. Thus, results for these two analyses reflect the specific sites sampled and should not be extrapolated to the entire NWCA sampled population.

### Characterization of the 2011 NWCA sampled population

**Fig. 2:**
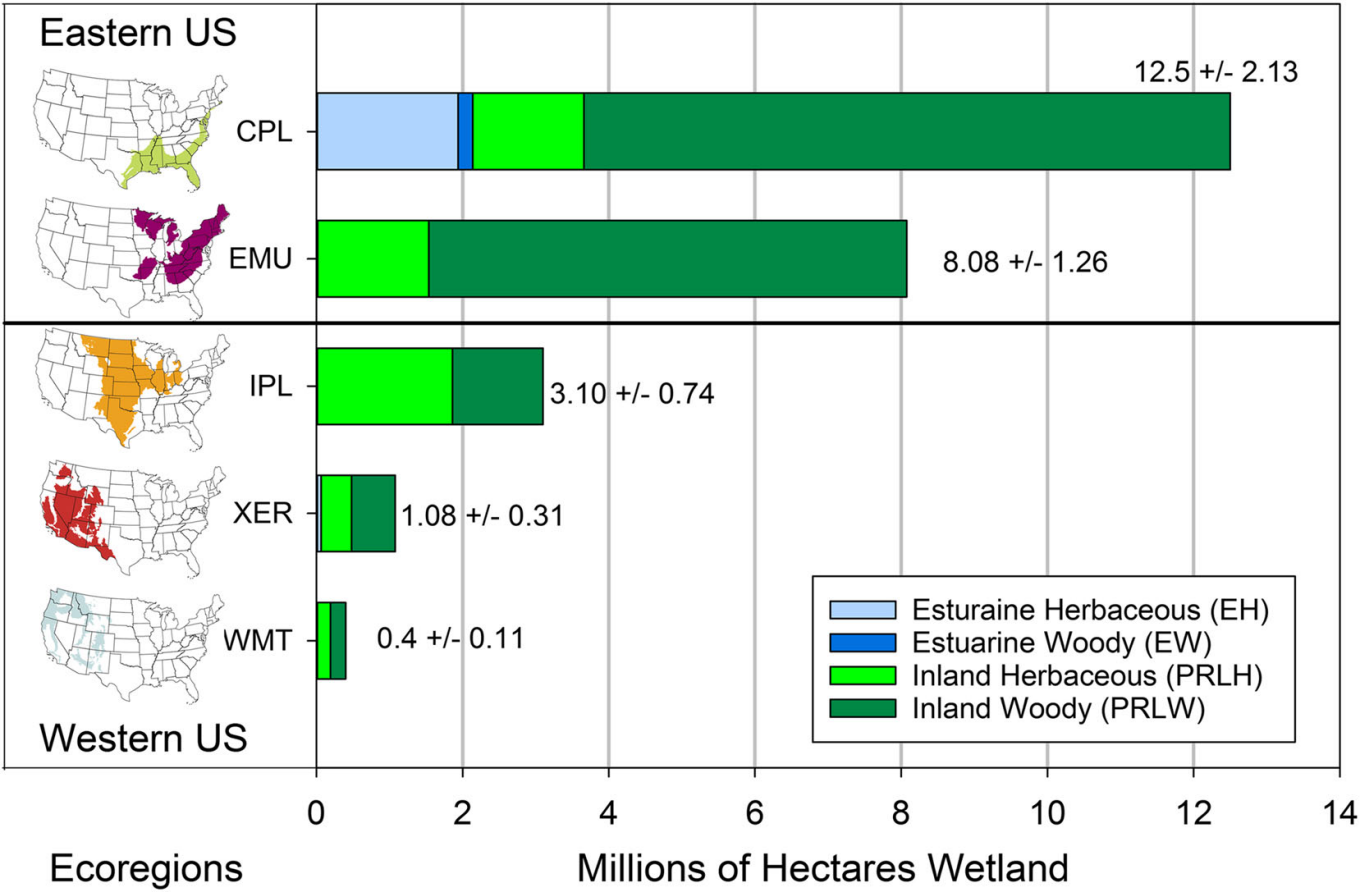
Estimated wetland area in millions of hectares by wetland type within ecoregions for the National Wetland Condition Assessment sampled population. Ecoregions: CPL = Coastal Plains, EMU = Eastern Mountains and Upper Midwest, IPL = Interior Plains, XER = Xeric West, WMT= Western Mountains and Valleys. See Table 3 for wetland type (EH, EW, PRLH, PRLW) definitions. See Supplement 2—Table A for tabular presentation of these results including 95% confidence intervals for wetland type area and number of sampled probability sites on which area estimates were based

The wetland area of the 2011 NWCA target population across the conterminous US was estimated at approximately 38 million ha; however, approximately one third of this area was represented by sites selected by the design, but not sampled due to denial of access by land owners, inaccessibility, or safety constraints (Olsen et al. 2019). Consequently, the NWCA sampled population, characterized by the 967 sampled probability sites, encompassed a subset of the NWCA target population area and represents approximately 25 million ha of wetland (USEPA 2016d). In the sampled population, both area and wetland types are unequally distributed across the five major ecoregions (Fig. 2, Supplement 2 (S2)—Table A, and Table 4). This unequal distribution of wetland area and types was driven, in part, by the survey design, which accounted for the spatial distribution of wetlands across the US, and in part, because access issues precluded sampling some sites identified in the survey design (Olsen et al. 2019). As a result, the percent of the NWCA target population area accounted for in the sampled population was less in some ecoregions (Xeric West, Western Mountains and Valleys) and some wetland types (EW—estuarine woody, PRLH—inland herbaceous) than others (Table 6). Nonetheless, the scale of the overall sampled population and subpopulations, represented by the probability-based 2011 NWCA data set, is unique in areal extent and proportion of the wetland resource described with ecological data.

**Table 6:** Estimated area in millions of hectares (ha) for wetland target population and the percent of target population represented by sampled (inference) population and subpopulations

Region	Estimated area target population (millions ha)	% Target area represented by sampled population
Conterminous US	38.41 ± 2.31	65 ± 3.9
Ecoregion		
CPL	19.70 ± 1.51	63 ± 5.7
EMU	10.00 ± 1.16	80 ± 8.3
IPL	4.97 ± 0.74	62 ± 7.7
XER	2.08 ± 0.35	52 ± 9.5
WMT	1.65 ± 0.56	24 ± 10.1
Wetland type		
EH	2.27 ± 0.42	89 ± 4.0
EW	0.40 ± 0.12	50 ± 16.8
PRLH	10.41 ± 0.92	53 ± 5.7
PRLW	25.34 ± 2.01	69 ± 5.5

Margin of error estimates are two-sided 95% confidence intervals. See Fig. 1 for ecoregion definition, Table 3 for wetland type descriptions, and Table 4 for sampled population areas

To provide a general description of the NWCA ecoregion and NWCA wetland type subpopulations, we looked at population-weighted means for several native vegetation attributes and environmental characteristics (Table 7). Mean native species richness varied ecoregionally (Table 7): greatest in the Eastern Mountains and Upper Midwest (46), followed by the Western Mountains and Valleys (35), somewhat less in the Interior Plains (29) and Coastal Plains (27), and least in the Xeric West (10). Mean native richness also varied by wetland type (PRLW—inland woody = 39, PRLH = 24, EW = 13, EH—estuarine herbaceous = 4), and was greater for woody than herbaceous types and greater in inland than estuarine systems. Numerous kinds of wetland exist in inland settings (e.g., bottom-land deciduous forests, coniferous dominated wetlands, fens, bogs, marshes, wet prairie, potholes), many of which have high site-level richness (Mitsch and Gosselink 2007), likely contributing to the greater mean richness across the NWCA inland wetland types. In contrast, the lower mean richness observed for estuarine wetlands may result, in part, from requirements for species to be adapted to saline conditions and tidal fluctuation of water levels (Keddy 2000).

**Table 7:** Population-weighted means and 95% CI for characteristics describing natural vegetation and environmental conditions by NWCA ecoregion (see Fig. 1) and by NWCA wetland type (Table 3)

Characteristics	NWCA ecoregions					NWCA wetland types			
	CPL	EMU	IPL	XER	WMT	EH	EW	PRLH	PRLW
Native richness (no. of native species)	26.64 ± 1.88	45.67 ± 5.03	28.61 ± 6.75	9.66 ± 1.92	35.02 ± 1.92	4.22 ± 1.23	13.29 ± 3.29	23.57 ± 3.72	38.67 ± 2.81
Native forb absolute cover	15.60 ± 3.48	22.96 ± 4.49	28.88 ± 5.87	11.06 ± 3.91	23.65 ± 5.04	5.38 ± 1.58	4.38 ± 2.94	24.52 ± 4.34	19.77 ± 3.22
Native graminoid absolute cover	23.05 ± 3.98	31.52 ± 6.76	24.44 ± 6.72	19.82 ± 6.60	37.23 ± 6.69	75.43 ± 11.78	28.32 ± 11.01	38.39 ± 5.79	16.37 ± 3.25
Native shrub absolute cover	11.69 ± 3.00	21.94 ± 6.76	2.77 ± 2.12	2.98 ± 2.13	17.49 ± 5.89	4.19 ± 4.17	8.11 ± 6.64	3.93 ± 2.58	17.81 ± 3.75
Native tree absolute cover	68.78 ± 8.58	71.69 ± 11.91	32.67 ± 11.86	2.28 ± 1.67	16.27 ± 5.50	2.67 ± 2.39	52.90 ± 11.10	7.18 ± 2.22	85.69 ± 7.13
Native vine absolute cover	13.14 ± 2.32	1.94 ± 1.14	3.12 ± 2.19	0.08 ± 0.06	1.16 ± 1.14	0.33 ± 0.19	1.11 ± 0.50	0.87 ± 0.42	10.58 ± 1.83
Wetland index (1–5, 1 = wettest)	2.16 ± 0.08	1.98 ± 0.16	2.04 ± 0.17	2.59 ± 0.22	2.38 ± 0.20	1.38 ± 0.09	1.73 ± 0.42	1.74 ± 0.12	2.32 ± 0.08
Bareground cover (0–100%)	5.35 ± 0.97	3.52 ± 1.17	11.02 ± 3.01	23.77 ± 12.49	3.51 ± 0.80	3.64 ± 1.32	10.02 ± 4.41	7.52 ± 1.98	6.07 ± 1.34
Mean annual precipitation (cm)	134.47 ± 2.05	86.36 ± 2.31	66.82 ± 5.01	24.59 ± 3.24	77.24 ± 11.72	137.20 ± 8.19	138.27 ± 5.68	82.38 ± 5.04	108.14 ± 2.90
Elevation (m)	26.39 ± 3.61	320.41 ± 19.58	439.14 ± 55.17	1033.56 ± 106.31	1953.49 ± 174.36	1.30 ± 0.27	1.38 ± 0.36	405.25 ± 5.08	225.86 ± 22.63

See Table 4 for estimated wetland area by NWCA ecoregion and wetland types and for the number of sampled probability sites on which population-weighted means are based

Absolute cover of native species within growth-habit groups (Table 7) was used to describe natural vegetation structure, recognizing this natural structure could be impacted where native vegetation is strongly altered by human-mediated disturbance or replaced by nonnative plant species (Magee et al. 2008). Native vegetation structure varied most strongly by wetland type. For example, mean native forb cover was about 20 to 25% in inland wetland types, but only about 5% in estuarine wetland types. In contrast, mean native graminoid cover was greater in herbaceous (EH ~ 75%, PRLH ~ 38%) than woody (EW ~ 28%, PRLW ~ 16%) systems. Mean native shrub and tree cover were greatest in the woody-dominated wetlands (EW, trees ~ 52%, shrubs ~ 8%; PRLW, trees ~ 86%, shrubs ~ 18%) as would be expected, and mean native tree cover was least in wetlands of the Xeric West (~ 2%) and Western Mountains and Valleys (~ 16%). Means for native vine cover were generally low, but vines were most prominent in inland woody wetland (PRLW ~ 11%) and in the Coastal Plains (~ 13%). The distribution of native species cover by growth-habit groups is likely to influence the trait requirements of nonnative species most adapted to invading different plant community types (Weihe and Neely 1997; Brewer 2011; Pyšek et al. 2012).

Not surprisingly, the largest differences in environmental conditions (Table 7) were observed across ecoregional subpopulations. Differences in the mean WI indicated wetlands in the Coastal Plains, the Eastern Mountains and Upper Midwest, and the Interior Plains had, on average, somewhat wetter overall hydrologic conditions than those in the Western Mountains and Valleys and the Xeric West. Among wetland types, the WI indicated the PRLW had on average somewhat drier conditions compared to other types. Mean annual precipitation was greatest in the Coastal Plains (~ 135 cm), intermediate in the Eastern Mountains and Upper Midwest (~ 86 cm) and the Western Mountains and Valleys (~ 77 cm), somewhat less in the Interior Plains (~ 67 cm), and least in the Xeric West (~ 25 cm). WI and annual precipitation means may reflect general differences in available moisture that could facilitate or hinder some nonnative taxa over in situ native taxa, depending on the moisture regimes to which individual nonnative species are adapted (e.g., Magee and Kentula 2005; Dwire et al. 2006). Mean percent cover of exposed bareground was greatest in the Interior Plains (~ 11%) and Xeric West (~ 24%). Wetlands with more exposed bareground (i.e., lacking vegetation or vegetative litter), whether from natural processes or from human-mediated disturbance, could provide microsites for establishment of nonnative species (e.g., Quinn and Holt 2008). Mean elevation increased from east to west across the country, beginning in the Coastal Plains at 26 m and ranging to 1953 m in the Western Mountains and Valleys. Elevation represents gradients of temperature, moisture, and growing season length that can be expected to influence establishment or competitive ability of natives and nonnatives and select for nonnatives adapted to specific conditions (e.g., Averett et al. 2016).

### Characterization of the complement of observed nonnative plant taxa

Across the 1138 sites sampled in the 2011 NWCA, 443 unique nonnative plant taxa were detected (see Supplement 1—Ordered table of nonnative plants) and represented 12% of the total number (3640) of taxa observed (USEPA 2016d). Based on occurrences at the 2011 sampled sites, 80 of the observed nonnative taxa were found in three or more of the five NWCA ecoregions (see Supplement 1). More than half the 443 nonnative taxa were forbs (54%) and 20% were graminoids, with trees (9%), shrubs (7%), and vines (10%) encompassing smaller percentages of the observed nonnative flora. The number of nonnative taxa observed per site ranged from 0 to 29. Total nonnative cover (sum of absolute percent covers for all nonnative taxa occurring at a location) also varied markedly across individual sampled sites, ranging from 0 to 160%. The array of nonnatives present, including the complement of specific taxa and growth habits (Supplement 1), suggests a species pool adapted to many ecological conditions, consequently representing diverse opportunities for invasion and interactions among nonnatives with varying impacts to native plant communities (e.g., Pyšek et al. 2012; Barney et al. 2013; Brewer and Bailey 2014; Kuebbing et al. 2014; Kuebbing et al. 2015; Rai 2015; Giorgis et al. 2016).

The 443 individual nonnative taxa encountered in the 2011 NWCA were found at between 1 and 166 of the sampled sites; 327 nonnative taxa were found at 5 or fewer sites, 50 occurred at 6–10 sites, and 51 occurred at 11–40 sites. Fifteen nonnative taxa were observed at more than 40 of the sampled sites: *Phalaris arundinacea* L. (*n* = 166), *Poa pratensis* L. (*n* = 116), *Taraxacum officinale* F.h. Wigg. (*n* = 114), *Phragmites australis* (Cav.) Trin. Ex Steud. (*n* = 108), *Rumex crispus* L. (*n* = 96), *Cirsium arvense* (L.) Scop. (*n* = 77), *Typha angustifolia* L. (*n* = 67), *Bromus inermis* Leyss. (*n* = 50), *Typha* ×*glauca* Godr. (Pro Sp.) (*n* = 49), *Phleum pratense* L. (*n* = 46), *Solanum dulcamara* L. (*n* = 46), *Rosa multiflora* Thunb. (*n* = 44), *Elymus repens* (L.) Gould (*n* = 43), *Lonicera japonica* Thunb. (*n* = 41), and *Triadica sebifera* (L.) Small (*n* = 41). The mean importance value (IV = (cover + frequency)/2) across sites of occurrence for these 15 most frequently observed nonnative taxa ranged from 25 to 47. Nonnative taxa with higher mean importance at sites of occurrence may presage their increased invasiveness in wetlands going forward (e.g., Randall et al. 2008; Ehrenfeld 2010; Magee et al. 2010). In addition, 82% of the observed nonnative taxa are recognized as invasive, noxious, weedy, or invaders of natural areas (see Supplement 1). Although many of these aggressive taxa had low importance or were found only at a small number of NWCA sampled sites in 2011, the large number of nonnative taxa suggests a substantial invasion debt (sensu, Seabloom et al. 2006; Essl et al. 2011) for wetlands. At least some of these taxa are likely to expand in abundance and distribution and to have increasing impact over time; particularly, where (1) propagule pressures increase, (2) lag times in population growth or to reproductive maturity are overcome, (3) shifts in environmental conditions occur, (4) human-mediated disturbance increases (Seabloom et al. 2006; Rouget et al. 2016; Antunes and Schamp 2017; Dwire et al. 2017), or (5) with the advent of synergies among co-occurring nonnatives (Kuebbing et al. 2013; Kuebbing and Nuñez 2016).

### Estimates of wetland area by NNPI stressor-level

**Fig. 3:**
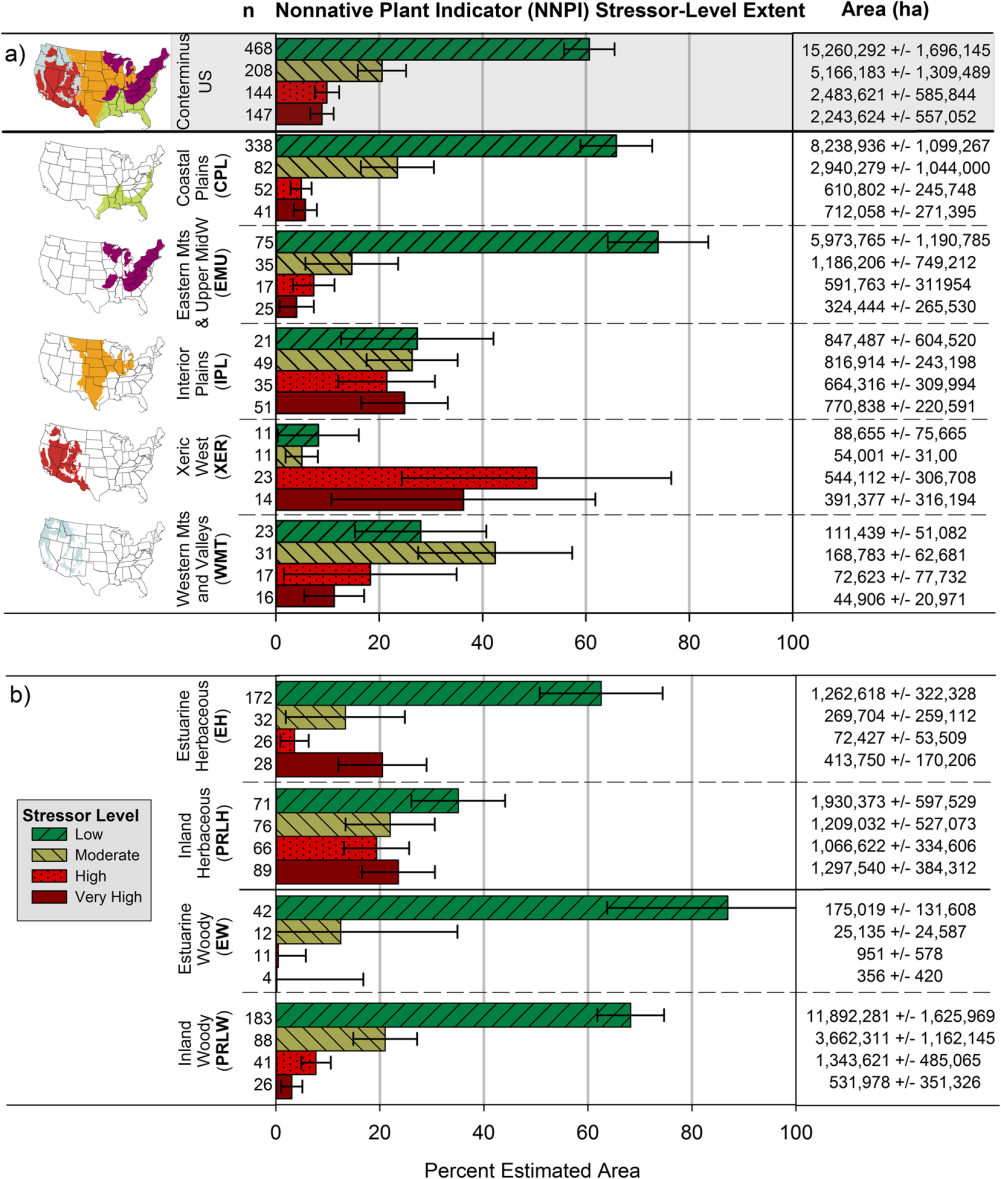
Estimated wetland area in the National Wetland Condition Assessment sampled population by stressor-level category (low, moderate, high, and very high) for the nonnative plant indicator (NNPI). Subpopulation results are displayed in horizontal panels with bar charts reflecting percent area in each NNPI stressor-level; area in hectares (ha) is listed to the right of each bar. Results in graph **a** are for the conterminous US and ecoregional subpopulations and in graph **b** for wetland type subpopulations. *n* = number of sampled probability sites on which area estimates are based. Margins of error are two-sided 95% confidence intervals. Note total wetland areas across ecoregions or across wetland types equal the total area in the NWCA sampled population at the national scale

The NNPI is a categorical indicator of ecological stress from the assemblage of nonnative plants occurring at a given location. To characterize the extent of the 2011 NWCA sampled population wetland area with varying impacts from nonnative species, percent area and area in hectares were estimated within each of the four NNPI stressor-level (low, moderate, high, and very high) categories for the national scale and for the five ecoregional and four wetland type subpopulations. Although about 61% of the estimated wetland area in the sampled population across the conterminous US had low NNPI, nearly 20% of the wetland area exhibited high or very high NNPI (Fig. 3a). The national-scale distribution of wetland area within different NNPI stressor-levels was mirrored by the pattern observed in the Coastal Plains and in the Eastern Mountains and Upper Midwest (Fig. 3a), which together encompass approximately 82% of the estimated wetland area in the sampled population (Fig. 2, Table 4). Even so, the percent area within specific NNPI stressor-levels varied markedly by ecoregion (Fig. 3a) and wetland type (Fig. 3b).

Based on percent area with high or very high NNPI, woody wetland types were less influenced by nonnatives than herbaceous wetland types, and estuarine herbaceous wetland appeared less affected than inland herbaceous wetland (Fig. 3b). About 68% of the inland woody (PRLW) wetland area had low NNPI, while about 11% of the area for this type fell in the high or very high categories. Estuarine woody (EW) wetland had the smallest percent area with high or very high NNPI; nearly all of its area was characterized as having low (87%) or moderate (12%) NNPI. In contrast, about 24% of the estuarine herbaceous (EH) wetland area fell into high or very high NNPI categories. Inland herbaceous (PRLH) wetland was the type most influenced by nonnative plants based on the extent estimates, with 43% of the area distributed between high and very high NNPI.

Nearly 90% of the wetland area in the eastern ecoregions (Coastal Plains and the Eastern Mountains and Upper Midwest) had low or moderate NNPI stressor-levels (Fig. 3a). In interpreting these results, it is important to note that the greatest area of PRLW wetland in the sampled population was found in these two eastern ecoregions, and all of the EW and most of the EH wetland occurred in the Coastal Plains (Fig. 2). These three wetland types each had large percent area with low or moderate NNPI (Fig. 3b) and, thus, contributed strongly to the prevalence of lower NNPI stressor-levels observed in the Coastal Plains and the Eastern Mountains and Upper Midwest (Fig. 3a). Although the NNPI stressor-level was low for these two regions in 2011, this could change over time in response to the complement of specific taxa that are present in the regional nonnative species pool (e.g., see Supplement 1 and the “Population means for absolute cover of nonnative growth-habit groups” section under “Exploratory analyses”).

The percent area with high and very high NNPI was much greater in three western ecoregions (Interior Plains, Xeric West, and the Western Mountains and Valleys) than in the eastern ecoregions (Fig. 3a). Combined percentages of wetland area with high and very high NNPI were about 45% for the Interior Plains, 86% for the Xeric West, and 29% for the Western Mountains and Valleys (Fig. 3a). Greater percent area in the higher stressor-levels for these ecoregions is likely related to greater percent area of PRLH wetlands compared to the Coastal Plains and the Eastern Mountains and Upper Midwest (Fig. 2), because the inland herbaceous type had the largest percent area with high and very high NNPI (Fig. 3b).

The sampled subpopulations for the Xeric West and the Western Mountains and Valleys represent about one half (52%) and one fourth (24%) of the target population area in these two regions, respectively (Table 6). Thus, it is possible that the 2011 sampled population may under- or overrepresent the amount of area with specific NNPI stressor-levels across the target population of wetlands in these two ecoregions. Nevertheless, the relatively small estimated wetland area in the target population for the Xeric West and the Western Mountains and Valleys (see Table 6 and Olsen et al. (2019)) and the large percent of the sampled population area with high or very high NNPI in the Interior Plains, Xeric West, and the Western Mountains and Valleys indicate nonnative plant species pose strong threats to wetlands in these three ecoregions.

### Exploratory analyses

The next four subsections include the results and discussion for four exploratory analyses that aid in the characterization of nonnative plants in wetlands at a variety of scales. Note, several individual species are highlighted in these subsections as examples illustrating particular points in the discussion, but they are not necessarily the most abundant or widespread nonnative taxa observed in the 2011 NWCA data.

#### Population means for NNPI metrics

**Fig. 4:**
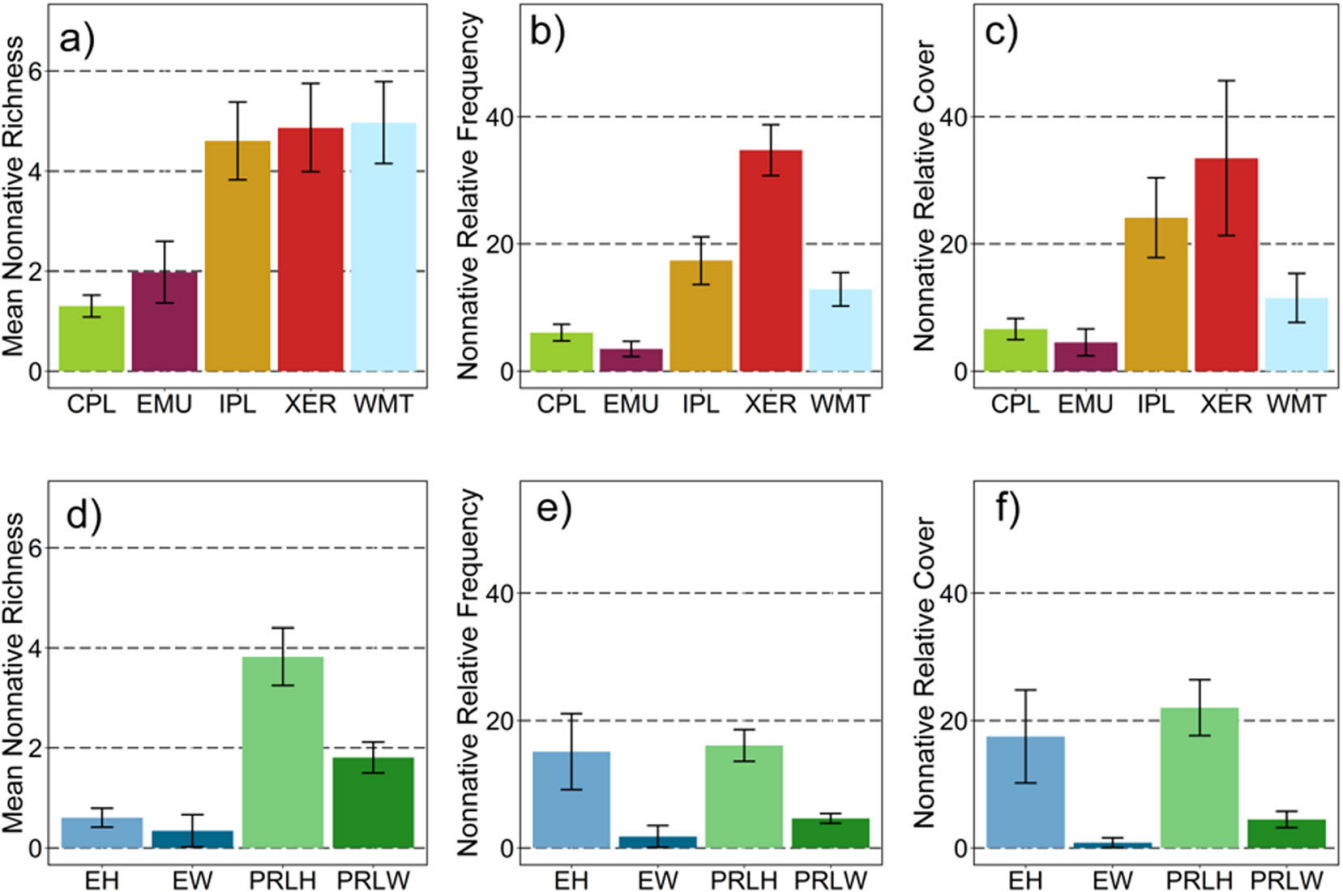
Population-weighted mean and 95% CI by ecoregion (**a**–**c**) and wetland type (**d**–**e**) for the three nonnative plant indicator (NNPI) component metrics (nonnative richness, nonnative relative frequency, and nonnative relative cover). Ecoregions: CPL = Coastal Plains, EMU = Eastern Mountains and Upper Midwest, IPL = Interior Plains, XER = Xeric West, WMT= Western Mountains and Valleys. Wetland type: EH = estuarine herbaceous, EW = estuarine woody, PRLH = inland herbaceous, PRLW = inland woody. See Table 3 for wetland type definitions. See Table 4 for estimated wetland area within NWCA ecoregion and wetland types and for the number of sampled probability sites on which population-weighted means are based. See Supplement 2—Table B for tabular presentation of these results

In our first exploratory analysis, we evaluated population-weighted means (± 95% CI) for each of the three NNPI metrics (Table 1) for ecoregional and wetland type subpopulations. Differences in means, based on nonoverlapping 95% CIs, for the three NNPI metrics (Fig. 4, S2-Table B) were observed among ecoregions and wetland types. As would be expected, these observations paralleled patterns in the area estimates by NNPI stressor-levels (Fig. 3), but the behavior of the individual metrics in the different subpopulations provides additional insights.

The western ecoregions (Interior Plains, Xeric West, and the Western Mountains and Valleys) all had mean nonnative richness (Fig. 4a) of approximately 5 species, compared to means of 1 to 2 nonnative species for the eastern ecoregions (Coastal Plains and the Eastern Mountains and Upper Midwest). Ecoregional patterns for nonnative relative frequency (Fig. 4b) and nonnative relative cover (Fig. 4c) were similar to one another, but both abundance metrics had lower values in the eastern than western ecoregions. Means for nonnative relative frequency and nonnative relative cover were similar in the Coastal Plains and the Eastern Mountains and Upper Midwest, ranging from about 4 to 7%. In the Western Mountains and Valleys, means for nonnative relative frequency (~ 13%) and nonnative relative cover (~ 12%) exceeded those in the eastern ecoregions, but were substantially less than in the Interior Plains and Xeric West. Mean nonnative relative frequency and nonnative relative cover were greater in the Xeric West (frequency ~ 35%, cover ~ 34%) than in the Interior Plains (frequency ~ 17%, cover ~ 24%), although for cover, the CIs overlapped. Among wetland types, mean nonnative richness (Fig. 4d) was least in the estuarine systems (EH, EW) and was greater for inland herbaceous (PRLH ~ 4) than inland woody (PRLW ~ 2) systems. Mean relative nonnative frequency (Fig. 4e) was greater for herbaceous (EH ~ 15%, PRLH ~ 16%) than for woody wetlands (EW ~ 1%, PRLW ~ 5%). Likewise, mean relative nonnative cover (Fig. 4f) was much greater for herbaceous (EH ~ 18%, PRLH ~ 22%) than for woody wetland types (EW ~ 1%, PRLW ~ 5%).

Several hypotheses about ongoing threats from nonnative species to specific subpopulations are suggested by these results. First, all else being equal, greater colonization pressure related to higher numbers of nonnative species in a given location or across a region will increase the likelihood of nonnative species establishing in new areas (Lockwood et al. 2009), which, in turn, increases the probability that a harmful nonnative taxon (Alpert 2006), or synergistic interactions among nonnatives (Ricciardi et al. 2013), will be added to a plant community. Thus, greater mean richness of nonnative species in the western ecoregions vs. eastern ecoregions (Fig. 4a), or in inland herbaceous (PRLH) vs. other wetland types (Fig. 4d), suggests these subpopulations may be most vulnerable to the risk that one (or more) of the nonnatives present is or will become invasive or an ecosystem engineer. In contrast, lower mean nonnative richness in estuarine wetlands than in inland wetlands (Fig. 4d) likely relates, in part, to the pool of nonnative taxa able to establish or spread in estuarine wetlands being limited to those adapted to brackish or saline conditions. Nevertheless, salt-tolerant nonnative ecosystem engineers with wide ecologic amplitude pose ongoing threats to estuarine systems. For example, *Phragmites australis* (Cav.) Trin. ex Steud. occurred at 108 NWCA sampled sites (Supplement 1), 78 of which were estuarine sites. Although this species has a native component (*Phragmites australis* (Cav.) Trin. ex Steud. ssp. *americanus* Saltonst., P.M. Peterson & Soreng), the aggressive nonnative subspecies (*Phragmites australis* (Cav.) Trin. ex Steud. ssp. *australis*) now dominates many inland and coastal marshes in the eastern US and has been increasingly observed in western locations (Chambers et al. 1999; Saltonstall 2002; Allen et al. 2017a). *P. australis* ssp. *australis* has the capacity to alter plant community composition and structure and ecosystem function (Silliman and Bertness 2004; Meyerson et al. 2010a; Uddin et al. 2017). In addition, the nonnative subspecies has been shown to hybridize with the native subspecies (Meyerson et al. 2010b; Saltonstall et al. 2014, 2016).

In the Xeric West and Interior Plains ecoregions (Fig. 4b) and for herbaceous wetland types (Fig. 4e), greater mean nonnative relative frequency may represent greater average numbers of nonnative foci per site for these subpopulations. More frequent occurrences of nonnatives can provide more locations from which nonnative taxa might disperse or expand across sites. In addition, higher values for relative frequency of nonnatives at a site could, in some circumstances, reflect increased potential for neighbor-to-neighbor competition between natives and nonnatives, possibly resulting in the reduced resiliency of the native plant community (e.g., Kuebbing and Nuñez 2016). High abundance or biomass of nonnative plants results in major changes in community composition and structure, which, in turn, often leads to alteration of ecosystem processes (e.g., Denslow and Hughes 2004; Hejda et al. 2009; Ehrenfeld 2010). Thus, the greater mean nonnative relative cover observed in 2011 (Fig. 4c) for wetlands of three western ecoregions compared to that observed in the two eastern ecoregions could reflect, on average, greater overall impact from nonnative plants in the western ecoregions. Given observed mean cover values, this is likely to be especially true for the Xeric West and Interior Plains regions. Further, although the mean nonnative relative cover in wetlands in the Western Mountains and Valleys was considerably less than for those in the Xeric West or Interior Plains (Fig. 4c), similar mean nonnative richness (Fig. 4a) suggests that wetlands in the Western Mountains and Valleys ecoregion may be at risk for increases in nonnative abundance. For example, some currently low cover nonnative species might transition from a lag phase (delayed or slow spread) to an expansion phase (rapid spread) of invasion (e.g., Simberloff 2011; Antunes and Schamp 2017).

#### Population means for absolute cover of nonnative growth-habit groups

Plant growth habit (e.g., graminoid, forb, vine, tree/shrub) is often related to other functional traits such as relative growth rate, height, or biomass (Lavorel et al. 2007), so the growth-habit type of nonnative plants can differentially influence their success in diverse ecosystems and environments (e.g., Herron et al. 2007; Tecco et al. 2010; Giorgis et al. 2016). Across the individual probability sites (*n* = 967) sampled in the 2011 NWCA, there was a wide range in absolute cover values for nonnative forbs (0–98%), graminoids (0–101%), shrub/trees (0–81%), and vines (0–34%). However, we wondered if the abundance of growth habits for nonnatives might vary at the scale of wetland populations. Thus, our second exploratory analysis was aimed at examining whether certain growth-habit types of nonnative taxa tended to be more abundant than others in different NWCA subpopulations.

**Fig. 5:**
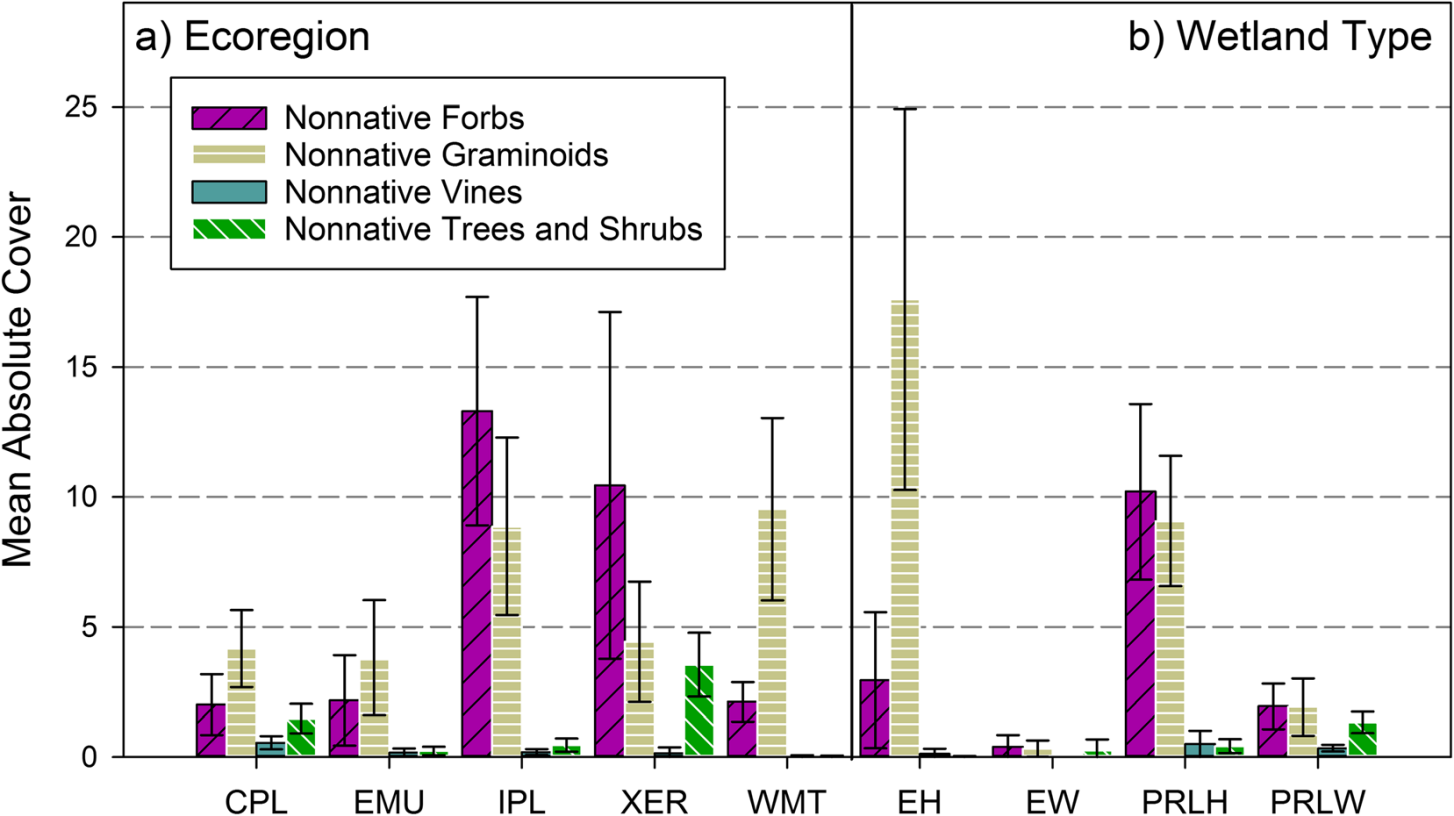
Population-weighted mean and 95% CI for the nonnative absolute percent cover in four growth-habit groups (forbs, graminoids, vines, and trees and shrubs) by ecoregion (**a**) and wetland type (**b**). Ecoregions: CPL = Coastal Plains, EMU = Eastern Mountains and Upper Midwest, IPL = Interior Plains, XER = Xeric West, WMT= Western Mountains and Valleys. Wetland type: EH = estuarine herbaceous, EW =estuarine woody, PRLH = inland herbaceous, PRLW = inland woody. See Table 3 for wetland type definitions. See Table 4 for the number of sampled probability sites and the estimated wetland area within the NWCA ecoregion and wetland types on which population-weighted means are based. See Supplement 2—Table C for tabular presentation of these results

We calculated population-weighted means (± 95% CI), for nonnative absolute cover by four growth-habit groups for the five ecoregions and four wetland types (Fig. 5, S2-Table C). Across ecoregions (Fig. 5a), the greatest nonnative cover was observed for the forb (means ranging from ~ 2 to 13%) and graminoid (means ranging from ~ 4 to 10%) groups. Nonnative absolute cover in the Western Mountains and Valleys was dominated by graminoids (mean ~ 10%). Although CIs were overlapping in the other four ecoregions, nonnative forbs tended to have greater mean cover than nonnative graminoids in the Interior Plains and Xeric West, whereas the reverse was true in the Coastal Plains and in the Eastern Mountains and Upper Midwest. Forbs and graminoids also made up most of the nonnative cover in the inland and estuarine herbaceous wetland types (Fig. 5b), with mean nonnative cover of forbs and graminoids about equal (~ 10%) in the PRLH, and graminoids the most abundant nonnative growth-habit group (~ 18%) in the EH. In contrast, the nonnative vine and tree/shrub groups had low mean cover (< 1 to about 3.5%) across all subpopulations (Fig. 5a, b**)**. Mean nonnative vine cover was greatest in the Coastal Plains (Fig. 5a) but still represented only a small amount of the nonnative plant cover for wetlands in this region. The greatest values for mean nonnative tree/shrub cover were observed in the Coastal Plains (~ 1.5%) and Xeric West (~ 4%) (Fig. 5a) and in inland woody wetlands (PRLW ~ 1.4%) **(**Fig. 5b). Based on these results, the population-scale means for absolute cover indicate that in 2011, on average, graminoid and forb nonnative taxa had more extensively invaded wetlands across the five ecoregions of the sampled population than taxa in the vine or tree/shrub groups, especially for herbaceous wetland types.

This pattern is perhaps not unexpected because, globally, numerous nonnative forb and grass (Poaceae) species are known to be highly successful invaders and many have been associated with ecological impact (Pyšek et al. 2012, 2017; Linder et al. 2018). Forb species that are tall and capable of forming populations with cover greater than the dominant native species, for example, have been shown to negatively impact plant communities (e.g., Hejda et al. 2009). Nonnative grasses are particularly successful because of their capacity to colonize, persist, dominate vegetation, and transform environments with impacts on ecosystem processes, resource availability, and local disturbance regimes (Martina and von Ende 2013; Gebauer et al. 2016; Linder et al. 2018). Canopy cover from trees and shrubs likely limits the establishment of shade-intolerant nonnative forbs and graminoids in woody wetlands. However, shade-tolerant nonnative taxa of all growth forms are often competitive in forested settings, and many have detrimental long-term impacts (Martin et al. 2009). Consequently, even though the mean absolute cover for nonnative forbs and graminoids in woody wetlands in the NWCA population was low in 2011, going forward, shade-tolerant nonnative forbs and graminoids may pose greater threat as they come into equilibrium with their potential introduced ranges. For instance, in the 2011 NWCA, the shade-tolerant annual grass, *Microstegium vimineum* (Trin.) A. Camus, was found at 20 sampled sites with a mean importance of 35 at sites of occurrence (Supplement 1). *M. vimineum* forms dense monocultures and alters nitrogen cycling (DeMeester and Richter 2010) and soil microbial communities (Kourtev et al. 2002; North and Torzilli 2017), and its greatest negative community-level effects occur in shady forested settings (Brewer 2011; Brewer and Bailey 2014).

Although mean absolute cover of nonnative vines (including lianas and species transitional between vines and shrubs) was low across the NWCA sampled population (Fig. 5), it was greatest in the Coastal Plains and in inland herbaceous wetlands (PRLH), and wetlands in these subpopulations may be at increased risk for expansions of nonnative vine cover in the future. A diverse set of 24 nonnative vine species was observed in the Coastal Plain sites sampled in 2011; many of these vine taxa are strong invaders (see Supplement 1, * next to PLANTS Symbol). Invasive vines can usurp space, overtop other vegetation, alter availability of light and nutrients, change habitat to facilitate other invasive taxa, and alter fire regimes (e.g., *Lonicera japonica* Thunb. (GISD 2018) and *Lygodium japonicum* (Thunb.) Sw. (CABI 2018b)). In 2011, *Lonicera japonica* occurred at 41 NWCA sampled sites (35 in the Coastal Plains) and *Lygodium japonicum* was found at 10 sites (all in the Coastal Plains) (Supplement 1). In addition to the 24 vine taxa primarily observed in the Coastal Plains in 2011, other vine species were found across sampled sites in one or multiple ecoregions, and many of these were also recognized as invasive (Supplement 1). For example, *Solanum dulcamara* L. was found in 46 sampled sites across all five ecoregions and *Rubus armeniacus* Focke was observed only at 4 sites in the Western Mountains and Valleys; however, both are highly invasive and readily overtop native vegetation (Waggy 2009; CABI 2018c).

Mean absolute cover values for the nonnative tree/shrub group tended to be low; however, inland woody systems had greater mean nonnative cover for this growth-habit group than other wetland types (Fig. 5b). The Coastal Plains and the Eastern Mountains and Upper Midwest ecoregions had the greatest prevalence and percent of woody wetland area (Fig. 2), and across all 2011 NWCA sites sampled for these two ecoregions, 43 nonnative taxa in the tree/shrub growth-habit group were observed, with 9 of these taxa detected in both regions, and all 43 are recognized as invasive, noxious, weedy, and/or known invaders of natural areas (Supplement 1). Thus, it will be important to watch for changes over time in the amount of woody wetland area with high or very high NNPI. Due to tall stature, canopy structure, and longevity, nonnative trees and shrubs are often ecosystem engineers that can alter overall plant community structure and composition, nutrient inputs, soil biota, and light regimes (Reinhart et al. 2006). As a result, nonnative trees and shrubs may have potential for increased expansion in cover and impact over time in both herbaceous and woody systems, as lag times related to height growth and reproductive age are overcome (e.g., Martin et al. 2009). Nonnative tree or shrub species with wide ecologic amplitude could have potential impacts on a variety of wetland types. For instance, the introduced tree *Triadica sebifera* (L.) Small thrives in fresh to saltwater and in sunny to shady conditions, displaces native plant species, forms monotypic stands, and alters nutrient cycles (GISD 2015), and it was observed in the Coastal Plains ecoregion at 41 NWCA sampled sites encompassing all four NWCA wetland types. Among ecoregions, the Xeric West had the highest population mean for absolute nonnative tree/shrub cover (Fig. 5a), and this result likely reflects occurrences of *Tamarix chinensis* Lour. and *Elaeagnus angustifolia* L., which were the two most commonly observed species in this growth habit for sampled sites in this ecoregion (Supplement 1). Both species are aggressive invaders and ecosystem engineers of riparian wetlands, altering successional trajectories as well as soil and hydrologic conditions (Lindgren et al. 2010; CABI 2018a).

#### Characterization of human-mediated disturbance in the sampled population

**Fig. 6:**
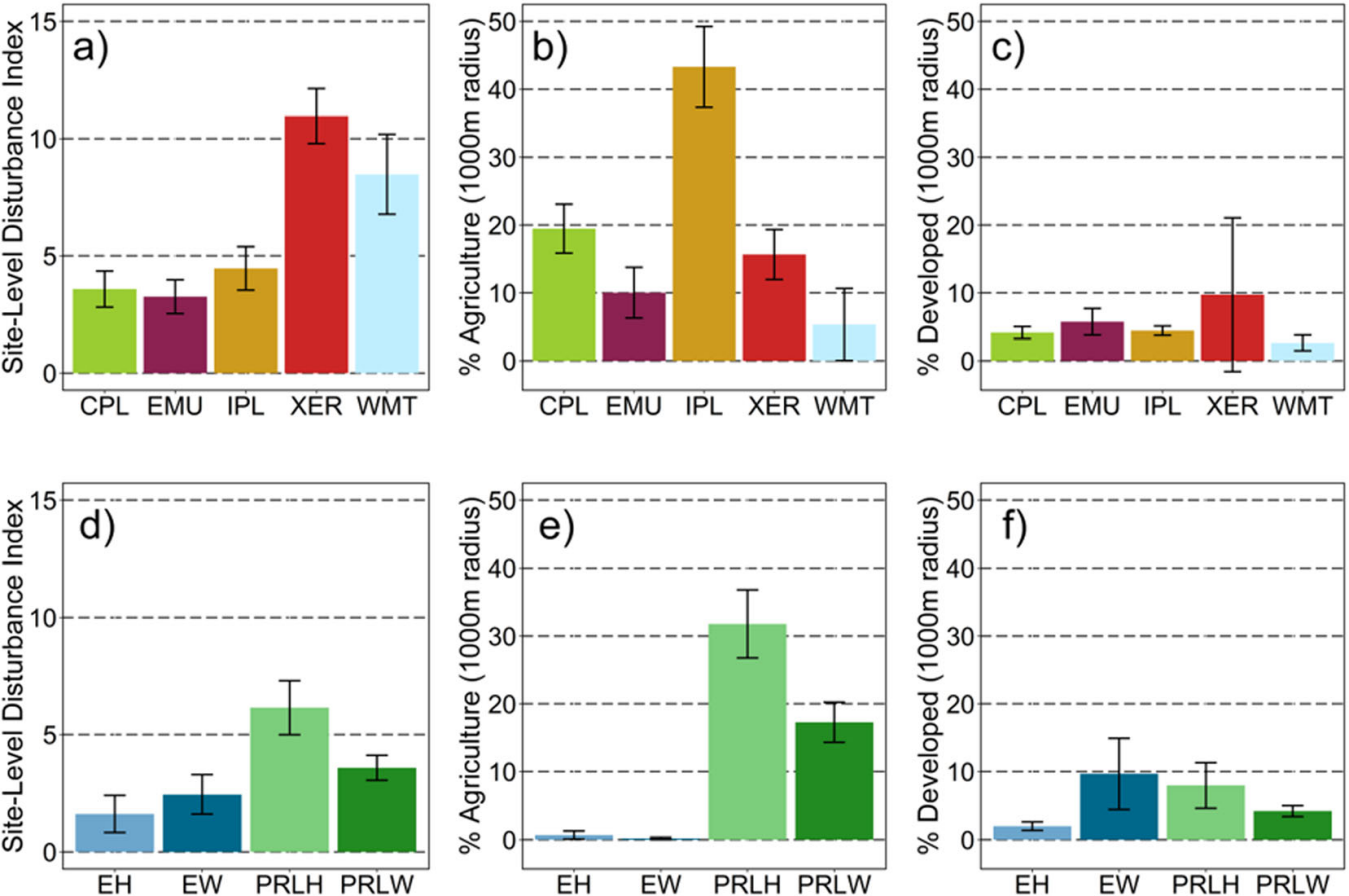
Population-weighted means and 95% CI (error bars) by ecoregion (**a**–**c**) and wetland type (**d**–**e**) for the three metrics of human-mediated disturbance (site-level disturbance index, and percent agriculture and percent developed land use in the 1000-m radius around each sampled site). Ecoregions: CPL = Coastal Plains, EMU = Eastern Mountains and Upper Midwest, IPL = Interior Plains, XER = Xeric West, WMT= Western Mountains and Valleys. Wetland type: EH = estuarine herbaceous, EW = estuarine woody, PRLH = inland herbaceous, PRLW = inland woody. See Table 3 for wetland type definitions. See Table 4 for estimated wetland area within the NWCA ecoregion and wetland types and for the number of sampled probability sites on which population-weighted means are based. See Supplement 2—Table D for tabular presentation of these results

Although not all nonnative plant species require disturbance to invade natural plant communities, human-mediated disturbances (either at the site level or in the surrounding landscape) are often linked to increased levels of invasion by nonnative plant species (e.g., Alpert et al. 2000; Silliman and Bertness 2004; Ehrenfeld 2008; Ringold et al. 2008; Brewer and Bailey 2014; Herlihy et al. 2019b). So, in our third exploratory analysis, we examined three descriptors of human-mediated disturbance (Fig. 6, S2-Table D) to see if, at the scale of the 2011 NWCA ecoregional and wetland type subpopulations, disturbance patterns paralleled NNPI extent results (Fig. 3).

We found that the population-weighted means for (1) the index describing overall site-level disturbance, and (2) for percent agriculture and percent developed land in the 1000-m surrounding each site, did vary by ecoregion (Fig. 6a–c) and wetland type (Fig. 6d–f). Mean values for the site-level disturbance index were greater in the Xeric West (~ 11) and the Western Mountains and Valleys (~ 9) compared to the other three ecoregions (Coastal Plains, Eastern Mountains and Upper Midwest, Interior Plains) where the mean ranged from about 3 to 5 (Fig. 6a). Among wetland types, site-level disturbance was greatest in the inland systems and was higher for the herbaceous (PRLH ~ 6) than the woody (PRLW ~ 4) type (Fig. 6d). Mean percent agriculture within the 1000-m radius surrounding each probability site (Fig. 6b) was greatest in the Interior Plains (~ 43%), substantial in the Coastal Plains (~ 20%) and Xeric West (~ 16%), somewhat less for the Eastern Mountains and Upper Midwest (~ 10%), and lowest in the Western Mountains and Valleys (~ 5%). Results also show that mean percent surrounding agriculture was small in the subpopulation of estuarine wetlands, but much higher for the inland wetland subpopulations (PRLH ~ 32%, PRLW ~ 17), particularly for the herbaceous type (Fig. 6e). Values for mean percent developed land in the 1000-m surrounding probability sites were relatively low (< 10% with CIs tending to overlap) both for ecoregions (Fig. 6c) and wetland types **(**Fig. 6f), but was least for estuarine herbaceous wetland (EH ~ 2%), and greatest for the estuarine woody (EW ~ 10%) and inland herbaceous (PRLH ~ 8%) types.

Qualitative comparison of the disturbance results (Fig. 6) with percent area by NNPI stressor-level category for each wetland subpopulation (Fig. 3) suggests that a large percent area with high or very high NNPI is associated, at least in part, with high levels of human-mediated disturbance. This is most evident for site-level disturbance and percent surrounding agriculture, where the three ecoregions (Interior Plains, Xeric West, and Western Mountains and Valleys) with high means for these two disturbance metrics also have the greatest percent area with high and very high NNPI status (Fig. 3). In the Interior Plains, the high mean percent surrounding agriculture for wetlands may result in (1) propagule and colonization pressure from nonnative taxa associated with agricultural settings, (2) changes in wetland nutrient dynamics or influx of contaminants through fertilizer or pesticide run-off, and (3) changes in hydrology due to farming practices (e.g., tiling, irrigation run-off, or direct water extraction), all processes that can enhance the success of nonnative taxa. Overall site-level disturbance observed in 2011 was greatest for wetlands in the Xeric West and the Western Mountains and Valleys (Fig. 6a). Key elements of the site-level disturbance for these two regions are reflected in related NWCA work for the combined wetland area of the Xeric West and the Western Mountains and Valleys subpopulations, where approximately 61, 70, and 75% of this area were estimated to have high stressor-levels for vegetation removal (e.g., grazing, mowing), soil hardening (e.g., animal trampling, trails, impervious surfaces), and ditching, respectively (USEPA 2016c). These three physical stressors are likely to alter ecosystem properties (e.g., hydrology, vegetation structure, resource availability) and facilitate spread and establishment (e.g., via grazing animals, trail or road corridors, water transport of propagules) of nonnatives. Among the wetland type subpopulations, inland herbaceous wetland (PRL), in addition to having the greatest percent area in high or very high NNPI status (Fig. 3), also exhibited the greatest means for both site-level disturbance and percent agriculture (Fig. 6d, e).

#### Ecological relationships with site-level NNPI status

In our final exploratory analysis, we shifted from the population-scale to a site-scale focus to evaluate which ecological attributes might be most strongly associated with site-level NNPI status. Results discussed in previous sections suggested interactions between NNPI stressor-levels and ecoregion, wetland type, natural vegetation attributes, environmental characteristics, and disturbance descriptors. Consequently, we used RF analysis to explore how this set of ecological attributes and their potential interactions might collectively relate to the NNPI status at individual NWCA sampled sites. All sampled sites (*n* = 1138) and 15 predictor variables (ten native vegetation and environmental characteristics, Table 7; three disturbance descriptors, Fig. 6; NWCA ecoregion, Fig. 1; and NWCA wetland type, Table 3, Fig. 1) were included in the RF analysis.

**Fig. 7:**
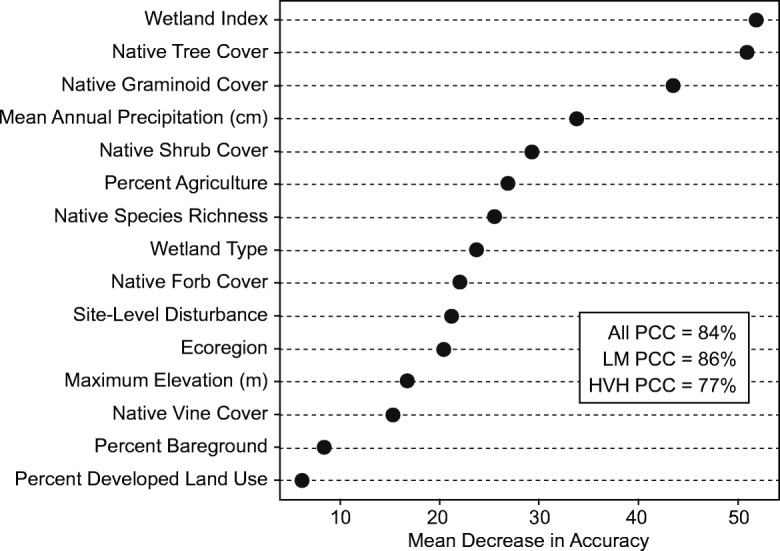
Variable importance plot for random forests classification of NNPI in two combined stressor-level categories (LM = low and moderate, HVH = high and very high). Predictor variables are listed on the left. Higher values for *mean decrease in accuracy* indicate greater importance of a predictor variable to the classification. Percent of sites correctly classified (PCC) by the model are listed in the text box for All, LM, and HVH sites

The RF analysis performed well, correctly classifying the two NNPI combined stressor-level categories (low–moderate and high–very high) 84% of the time for all sites, 86% of the time for low–moderate sites, and 77% of the time for high–very high sites (Fig. 7), based on the collective set of predictor variables. The RF variable importance plot (Fig. 7) shows the relative importance of each predictor in distinguishing sites likely to have high–very high vs. low–moderate NNPI stressor-levels. Partial dependence plots for all predictor variables are provided in Supplement 3 (S3), Figs. A–O, and indicate the specific relationship of each individual predictor with the probability of the occurrence of high–very high NNPI status. Note, all partial dependence plots exhibited nonlinear relationships. Considering the order of variable importance (Fig. 7) and partial dependence plots for the individual predictor variables used in the RF (S3) together highlights some key associations.

Wetland type (S3-Fig. H) and ecoregion (S3-Fig. K) were, respectively, the eighth and 11th most important predictors of NNPI (Fig. 7). This ranking is interesting because the 2011 area estimates for NNPI stressor-levels (Fig. 3) and the population-weighted means for the natural vegetation and environmental attributes (Table 7) and disturbance metrics (Fig. 6) varied with ecoregional and wetland type subpopulations. Nevertheless, although wetland type and ecoregion are important in predicting high–very high NNPI at the site level, other metrics may more specifically reflect site-level conditions associated with a given NNPI status.

Metrics related to moisture conditions were strong predictors based on variable importance in the RF analysis. The wetland index (WI) had the greatest variable importance (Fig. 7), with the likelihood of a site having high–very high NNPI status increasing with larger WI values (S3-Fig. A). Higher WI reflects lower abundance of obligate (OBL) or facultative wetland (FACW) indicator species and typically drier conditions (Wentworth et al. 1988). Among the 443 nonnative taxa observed across the 1138 sampled sites (Supplement 1), only 105 taxa had OBL or FACW indicator status. More than 75% of the observed nonnative taxa were facultative (FAC, no. of taxa = 88), facultative upland (FACU, no. of taxa = 117), or upland (UPL, no. of taxa = 116) indicators, suggesting that many of these nonnative taxa might be more prevalent at drier wetland sites. In addition, mean annual precipitation was the fourth most important predictor of NNPI status (Fig. 7), with probability of high–very high NNPI status greatest for sites where annual precipitation was less than 50 cm (S3-Fig. D).

Elevation and percent bareground, the two other environmental metrics evaluated, were among the weaker predictors in the suite of ecological attributes included in the RF, with variable importance at the 12th and 14th positions, respectively (Fig. 7). However, elevation greater than about 2500 m was associated with lower probability of having high–very high NNPI than lower elevations (S3-Fig. L). The 23 sampled sites with elevations greater that 2500 m were distributed in mountains across Arizona, Colorado, Nevada, New Mexico, and Utah. Higher elevations often are related to more extreme environments and to greater distance from nonnative propagule sources which could limit nonnative establishment; however, the growth form of invading nonnatives can differentially influence invasibility at higher elevations (Giorgis et al. 2016). Sites with less than about 25% bareground had much lower probability of having high–very high NNPI than sites with more bareground (S3-Fig. N), suggesting that in some situations greater amounts of available bareground may represent unexploited habitat for nonnatives (e.g., Quinn and Holt 2008).

Native vegetation structure is an ecosystem property that can influence the influx of nonnative plants (see discussion in the “Characterization of the 2011 NWCA sampled population” and “Population means for absolute cover of nonnative growth-habit groups” sections). This was reflected in absolute cover of native trees and native graminoids having the second and third highest variable importance in the RF analysis (Fig. 7). The probability of high–very high NNPI was greatest when there was no tree cover, decreased steadily as native tree cover increased from 0 to about 60%, then dropped precipitously until native tree cover exceeded 100%, where the probability of high–very high was lowest (S3-Fig. B). Greater cover of native trees can limit establishment and abundance of many shade-intolerant nonnatives. However, shade-tolerant nonnatives, including invasive shrubs, trees, graminoids, and vines, did occur in forest-dominated locations (Supplement 1), and, over time, these taxa might be expected to expand both their site-level cover and regional occurrence. Considering native graminoid cover alone (S3-Fig. C), the likelihood of having high–very high NNPI was least where native graminoids were abundant (e.g., 75 to 200% absolute cover), but increased more or less linearly as graminoid cover dropped from 75 to 0%. Greater native graminoid cover can contribute to exclusion of some nonnatives through competition and physical barriers where sod or thatch are formed.

Native absolute shrub and forb cover were the fifth and ninth most important predictors (Fig. 7), with the probability of having high–very high NNPI steadily increasing as native cover declined, beginning from about 50% cover for native shrubs (S3-Fig. E) and at about 100% for forbs (S3-Fig. I). Native shrubs may limit shade-intolerant nonnatives. Native vine cover was 13th in variable importance (Fig. 7), and the partial dependence plot did not indicate a clear response of NNPI to native vine cover alone (S3-Fig. M), possibly because native vine absolute cover tended to be low except in the Coastal Plains ecoregion (Table 7). In our RF model, native species richness was the seventh most important predictor (Fig. 7), with its partial dependence plot **(**S3-Fig. G**)** showing a complex pattern in relation to the probability of occurrence of high–very high NNPI stressor-level, which was greatest when native richness was low (e.g., about 15 or fewer species), least when native richness ranged from about 20 to 70 species, and intermediate when native richness was greater.

Human-mediated disturbance was related to increased probability of high–very high NNPI status. The disturbance metrics included in the RF analysis varied in importance. Percent agriculture in the 1000 m surrounding a site was the sixth most important predictor in the RF (Fig. 7), but among the three disturbance metrics, it had the strongest relationship to increasing likelihood of high–very high NNPI. The site-level disturbance index and the percent developed land in the 1000 m surrounding a site were the tenth and 15th most important predictor variables in the RF model, respectively (Fig. 7). Nevertheless, the partial dependence plots show marked increase in probability of occurrence of high–very high NNPI with increasing values for all three of the disturbance metrics (percent agriculture, S3-Fig. F; site-level disturbance, S3-Fig. J; percent developed land, S3-Fig. O), suggesting that increases in any of these disturbance measures are likely to be associated with greater stress from nonnatives.

This exploratory RF analysis effectively classified site-level NNPI status; so, the relative importance of predicator variables in this exploratory model is likely to be a useful starting point in informing monitoring or management efforts. For example, for a given ecological setting, NNPI status and site-level values for specific predictor variables might suggest possible pathways of risk for increasing impact from nonnative plants or management actions that could potentially aid in reducing nonnative success.

## Summary and next steps

As part of the 2011 NWCA, this study is unique in scope. It characterizes the status of nonnative plants in wetlands for a sampled population representing approximately 25 million ha of wetland and allows results to be viewed at a variety of scales, e.g., nationally, or for major ecoregional and wetland type subpopulations. To provide an ecological context for our work, we described the sampled population in terms of (1) estimated area by wetland type within ecoregions (Fig. 2) and (2) population-weighted means for a variety of ecological attributes describing natural vegetation structure and environmental characteristics by ecoregion and wetland type (Table 7). We used several approaches to evaluate nonnative plants in wetlands. First, we characterized the complement of nonnative plant species observed during the 2011 NWCA. The 443 nonnative plant taxa observed across NWCA sampled sites (Supplement 1) reflected a species pool adapted to many ecological conditions, thus providing diverse opportunities for continued invasion and potential impact to wetlands and other ecosystems.

Next, we considered potential impact from nonnative species, as indicated by the NNPI, by examining the extent of the 2011 NWCA sampled population (percent area and hectares of wetland) that fell into the four NNPI stressor-level categories (Fig. 3). Results at the scale of the conterminous US indicated nearly 20% of wetland area in the sampled population had high to very high NNPI. However, ecological stress from nonnatives varied markedly by ecoregion and wetland type. The extent patterns for NNPI stressor-levels observed at the national scale were driven by the large wetland area in the eastern ecoregions (the Coastal Plains and the Eastern Mountains and Upper Midwest) where nearly 90% of the wetland area was estimated to have low to moderate NNPI, which likely relates to the prevalence of woody wetland types in these two regions. Woody wetland types had greater percent area in lower NNPI stressor-level categories than herbaceous wetland types across the NWCA sampled population. In contrast, although the overall wetland area was much smaller in the western ecoregions (Interior Plains, Xeric West, and the Western Mountains and Valleys) than in eastern ecoregions, a much greater percent of the wetland area in the western regions had high to very high NNPI (ranging from about 27 to 87%). This pattern is related, at least in part, to the greater proportion of the wetland area in inland herbaceous types in the western ecoregions.

We also conducted a series of exploratory analyses that provide ecological information that aids in interpreting patterns described by the NNPI extent results. Three population-scale analyses examined ecoregional and wetland type population means for (1) the three NNPI metrics (nonnative richness, relative frequency, and relative cover, Fig. 4), (2) absolute cover for four growth-habit groups of nonnative plants (Fig. 5), and (3) three metrics describing human-mediated disturbance (Fig. 6). Results from these three analyses highlight some potential avenues of impact from nonnative plants, which vary ecoregionally and by wetland type and could suggest associations with the 2011 observed patterns for NNPI or suggest risks for future incursions of nonnatives. In addition, qualitative comparisons of the NNPI results with results of population-scale exploratory analyses suggested interactions between NNPI stressor-levels and ecoregion, wetland type, natural vegetation attributes, environmental characteristics, and human-mediated disturbance. Consequently, we examined ecological relationships with site-level NNPI status using a RF analysis (Fig. 7, Supplement 3) and NNPI as the response variable with predictor variables including ecoregion, wetland type, and a variety of characteristics describing natural vegetation structure, environment, and human-mediated disturbance. This exploratory RF correctly classified site-level NNPI for a combined high–very high class 77% of the time; thus, the relative importance of variables in this exploratory model may be useful in informing future research or monitoring efforts.

Examination of the NNPI patterns and the ecological relationships indicated by the exploratory analyses can help inform national or regional management and conservation priorities for wetlands, or suggest research questions that would support management needs. Moving forward, as additional data are collected in future NWCA assessments, changes over time in the NNPI stressor-levels can be used to assess whether impact from nonnative plants in wetlands is increasing. The second iteration of the NWCA was implemented in the field during the summer of 2016, with an expanded survey design to allow more complete representation of the wetland area across the conterminous US, particularly for the western part of the country (Olsen et al. 2019). As the 2016 data are considered, it will be possible to evaluate changes in wetland area within NNPI stressor-levels between 2011 and 2016. Finally, the results for the NNPI from the 2011 and 2016 assessments are likely to suggest a variety of specific questions that might be addressed in future research using NWCA data (USEPA 2016e). For example, modeling approaches (e.g., Seabloom et al. 2006; Thuiller et al. 2006; Schweiger et al. 2016; Hill et al. 2017) might be used to elucidate ecological correlates or mechanisms associated with stress from nonnative plants, or to predict and map locations with low or high stress from nonnatives at varying geographic scales.

## Electronic supplementary material

ESM 1 (PDF 1111 kb)

ESM 2 (PDF 122 kb)

ESM 3 (PDF 229 kb)
