# Supplementary material for: Characterizing nonnative plants in wetlands across the conterminous United States
Source: Environ Monit Assess. 2019 Jun 20;191(Suppl 1):344. doi: 10.1007/s10661-019-7317-3 (PMC6586712; doi:10.1007/s10661-019-7317-3)
Supplement: Supplementary file 1 — (PDF 1111 kb) [file 10661_2019_7317_MOESM1_ESM.pdf]

Supplement 1 - Ordered table of nonnative plant taxa observed at one or more of 1138 wetland sites sampled during the 2011 National Wetland Condition Assessment spanning the conterminous United States (US)

Journal: Environmental Monitoring and Assessment

Paper title: *Characterizing nonnative plants in wetlands across the conterminous United States*

Authors: Teresa K. Magee, Karen A. Blocksom, Alan T. Herlihy, and Amanda M. Nahlik

Corresponding Author: Teresa K. Magee, US Environmental Protection Agency, Office Research and Development, National Health Effects Laboratory, Western Ecology Division, Corvallis, Oregon

Ordered Table Arrangement: Nonnative plant taxa ordered by 1) growth form, 2) number of sites of occurrence across the conterminous US, and 3) co-occurrence across five major ecoregions. **Species occurring at five or more sites are highlighted in bold**.

Ecoregion Definitions: CPL = Coastal Plains, EMU = Eastern Mountains & Upper Midwest, IPL = Interior Plains, XER = Xeric West, WMT = Western Mountains and Valleys. See journal article Figure 1 for region boundaries.

Nomenclature follows (Scientific Name): USDA-NRCS (2014). The PLANTS Database (http://plants.usda.gov, 18 March 2014). **PLANTS Symbol** is the accepted symbol used by the PLANTS database.

*** Asterisk added to PLANTS symbol if taxon is invasive/noxious/weedy/invading natural areas in one or more US states:** Based on information in 1) USDA-NRCS (2017): The PLANTS Database (http://plants.usda.gov, November 2017), 2) Global Invasive Species Database (http://www.iucngisd.org/gisd/, November 2017), or 3) Invasive.org: Center for Species and Ecosystem Health (https://www.invasive.org, November 2017). Indicated here by an * following the PLANTS Symbol.

Wetland Indicator Status: USACE (2014). National Wetland Plant List, version 3.2, US Army Corps of Engineers (http://wetland_plants.usace.army.mil/) downloaded from PLANTS database to maintain consistency in nomenclature. OBL = obligate, FACW = facultative wetland, FAC = facultative, FACU = facultative upland, UPL = upland. See journal article for more detail.

State-Level Native Status: See journal article for details on how state-level native status was determined. INTR = introduced from outside the conterminous United States, ADV = adventive, that is native some locations in the conterminous United States, but introduced at the location of occurrence, CRYP = cryptogenic, that is having some elements (e.g., genotypes, varieties, subspecies, cultivars) that are native and some that are introduced. **Note**: For purposes of this study, INTR, ADV, and CRYP species were considered nonnative.

n = number of sites sampled across conterminous US or the number of the subset of sites that occurred in a specific ecoregion.

# Occur = the number of sampled sites at which a particular species occurred across the conterminous US or within a particular ecoregion.

Mean Importance ± Standard Error: the mean importance (± standard error) observed for a species across the sites at which it occurred nationally or by ecoregion, where importance at a site = (percent frequency + percent cover/2), possible values range from 0 - 100.

Status			Nonnative Plant Taxa	All Sampled Sites (n = 1138)		CPL (n=567)	EMU (n=214)	IPL (n=190)	XER (n=62)	WMT (n=105)
PLANTS Symbol*	Wetland Indicator Status	Native Status	Scientific Name	# Occur	Mean Importance ± Standard Error	# Occur	# Occur	# Occur	# Occur	# Occur
			Trees/Shrubs							
**TRSE6***	**FAC**	**INTR**	***Triadica sebifera* (L.) Small**	**41**	**36.25 ± 3.41**	**41**				
**SCTE***	**FAC**	**INTR**	***Schinus terebinthifolius* Raddi**	**7**	**26.41 ± 6.88**	**7**				
CICA*	UPL	INTR	*Cinnamomum camphora* (L.) J. Presl	4	10.30 ± 0.12	4				
MEAZ*	UPL	INTR	*Melia azedarach* L.	3	20.77 ± 5.91	3				

Status			Nonnative Plant Taxa	All Sampled Sites (n = 1138)		CPL (n=567)	EMU (n=214)	IPL (n=190)	XER (n=62)	WMT (n=105)
PLANTS Symbol*	Wetland Indicator Status	Native Status	Scientific Name	# Occur	Mean Importance ± Standard Error	# Occur	# Occur	# Occur	# Occur	# Occur
TAGA*	FACW	INTR	*Tamarix gallica* L.	3	17.67 ± 5.92	3				
URLO*	FAC	INTR	*Urena lobata* L.	3	21.64 ± 7.23	3				
RUPH*	NA	INTR	*Rubus phoenicolasius* Maxim.	2	10.01 ± 0.00	2				
ALJU*	UPL	INTR	*Albizia julibrissin* Durazz.	2	10.01 ± 0.00	2				
GLTR*	FAC/FACU	ADV occurrences	*Gleditsia triacanthos* L.	2	10.31 ± 0.30	2				
AIAL*	FACU	INTR	*Ailanthus altissima* (Mill.) Swingle	1	10.05	1				
POAL7*	UPL	INTR	*Populus alba* L.	1	21.70	1				
LIJA*	FAC	INTR	*Ligustrum japonicum* Thunb.	1	20.30	1				
MEQU*	FAC	INTR	*Melaleuca quinquenervia* (Cav.) S.f. Blake	1	10.01	1				
PSCA*	FACU	INTR	*Psidium cattleianum* Sabine	1	10.01	1				
SAAT2	FACW	INTR	*Salix atrocinerea* Brot.	1	10.10	1				
**LISI***	**FAC/FACU**	**INTR**	***Ligustrum sinense* Lour.**	**16**	**20.85 ± 3.88**	**15**	**1**			
**BETH***	**FACU/UPL**	**INTR**	***Berberis thunbergii* Dc.**	**10**	**25.68 ± 5.65**	**1**	**9**			
LIGUS2	NA	INTR	*Ligustrum* L.	3	26.70 ± 16.40	2	1			
SACI*	FACW	INTR	*Salix cinerea* L.	2	27.05 ± 16.95	1	1			
SANI4	FACW/FAC	CRYP occurrences	*Sambucus nigra* L.	2	20.20 ± 10.10		2			
**LOMO2***	**FACU**	**INTR**	***Lonicera morrowii* A. Gray**	**6**	**13.51 ± 2.14**		**6**			
PIAB*	UPL	INTR	*Picea abies* (L.) Karst.	4	17.71 ± 4.78		4			
LIVU*	FACU	INTR	*Ligustrum vulgare* L.	3	13.68 ± 3.18		3			
LOBE*	FACU	INTR	*Lonicera ×bella* Zabel	2	52.40 ± 21.20		2			
MALUS	NA	INTR	*Malus* Mill.	2	10.16 ± 0.15		2			
ACPL*	UPL	INTR	*Acer platanoide* s L.	1	22.10		1			
ROPS*	FACU/UPL	ADV occurrences	*Robinia pseudoacacia* L.	1	22.50		1			
PRAV*	NA	INTR	*Prunus avium* (L.) L.	1	10.01		1			
AEHI*	UPL	INTR	*Aesculus hippocastanum* L.	1	10.10		1			
MABA*	UPL	INTR	*Malus baccata* (L.) Borkh.	1	10.10		1			
SHAR	FACU	ADV	*Shepherdia argentea* (Pursh) Nutt.	1	12.00		1			
SOAU*	UPL	INTR	*Sorbus aucuparia* L.	1	20.02		1			
LIOB*	UPL	INTR	*Ligustrum obtusifolium* Siebold & Zucc.	1	20.02		1			
RUBTR*	UPL	INTR	*Rubus trifidus* Thunb.	1	10.01		1			
SPJA*	FACU	INTR	*Spiraea japonica* L. F.	1	20.21		1			

Status			Nonnative Plant Taxa	All Sampled Sites (n = 1138)		CPL (n=567)	EMU (n=214)	IPL (n=190)	XER (n=62)	WMT (n=105)
PLANTS Symbol*	Wetland Indicator Status	Native Status	Scientific Name	# Occur	Mean Importance ± Standard Error	# Occur	# Occur	# Occur	# Occur	# Occur
MAPU*	UPL	INTR	*Malus pumila* Mill.	2	10.11 ± 0.09		1	1		
**FRAL4***	**FACW/FAC**	**INTR**	***Frangula alnus* Mill.**	**22**	**27.08 ± 3.70**		**17**	**5**		
VIOP*	FACW/FAC	CRYP occurrences	*Viburnum opulus* L.	3	13.41 ± 3.35		2	1		
**MOAL***	**FAC/FACU/UPL**	**INTR**	***Morus alba* L.**	**16**	**22.55 ± 4.11**	**2**	**4**	**10**		
**RHCA3***	**FAC/FACU**	**INTR**	***Rhamnus cathartica* L.**	**16**	**25.86 ± 4.80**	**1**	**7**	**8**		
**LOTA***	**FACU**	**INTR**	***Lonicera tatarica* L.**	**13**	**23.88 ± 3.64**	**2**	**9**	**2**		
**LOMA6***	**UPL**	**INTR**	***Lonicera maackii* (Rupr.) Herder**	**5**	**14.13 ± 2.50**	**1**	**3**	**1**		
**ELUM***	**UPL**	**INTR**	***Elaeagnus umbellata* Thunb.**	**7**	**14.56 ± 4.29**	**1**	**4**	**2**		
**ELAN***	**FAC/FACU**	**INTR**	***Elaeagnus angustifolia* L.**	**12**	**25.40 ± 5.34**		**2**	**7**	**3**	
TARA*	FACW	INTR	*Tamarix ramosissima* Ledeb.	3	80.17 ± 1.33			3		
SAAL2	FACW	INTR	*Salix alba* L.	2	17.51 ± 6.51			2		
SAFR*	FAC	INTR	*Salix fragilis* L.	2	24.35 ± 14.15			2		
MAPO*	FACU	ADV	*Maclura pomifera* (Raf.) C.k. Schneid.	1	10.70 ± 0.00			1		
FRPE	FACW/FAC	ADV occurrences	*Fraxinus pennsylvanica* Marsh.	3	17.37 ± 3.65			2	1	
ULPU*	UPL	INTR	*Ulmus pumila* L.	2	11.05 ± 0.95			1	1	
**TACH2***	**FAC**	**INTR**	***Tamarix chinensis* Lour.**	**10**	**48.01 ± 4.59**				**10**	
ACSA2	FACW/FAC	ADV occurrences	*Acer saccharinum* L.	1	61.31				1	
FRQU	UPL	ADV	*Fraxinus quadrangulata* Michx.	1	11.20				1	
PONI*	UPL	INTR	*Populus nigra* L.	1	10.10				1	
AMFR*	FACW	ADV occurrences	*Amorpha fruticosa* L.	1	53.40				1	
CYSC4*	UPL	INTR	*Cytisus scoparius* (L.) Link	1	21.80					1
ILAQ80*	FACU	INTR	*Ilex aquifolium* L.	1	10.01					1
RUMA	UPL	INTR	*Rubus macrophyllus* Weihe & Nees	1	10.10					1
			Vine/Vine-Shrubs							
**LYJA***	**FAC**	**INTR**	***Lygodium japonicum* (Thunb.) Sw.**	**10**	**36.68 ± 5.67**	**10**				
**IPCO3***	**FAC**	**INTR**	***Ipomoea coccinea* L.**	**5**	**41.62 ± 5.44**	**5**				
CLTE4*	FACU	INTR	*Clematis terniflora* Dc.	3	10.04 ± 0.03	3				
ROBR*	UPL	INTR	*Rosa bracteata* J.c. Wendl.	2	28.71 ± 18.41	2				
HEHE*	NA	INTR	*Hedera helix* L.	2	15.11 ± 5.10	2				

Status			Nonnative Plant Taxa	All Sampled Sites (n = 1138)		CPL (n=567)	EMU (n=214)	IPL (n=190)	XER (n=62)	WMT (n=105)
PLANTS Symbol*	Wetland Indicator Status	Native Status	Scientific Name	# Occur	Mean Importance ± Standard Error	# Occur	# Occur	# Occur	# Occur	# Occur
LYMI*	FACW	INTR	*Lygodium microphyllum* (Cav.) R. Br.	1	10.10	1				
MIMI5*	UPL	INTR	*Mikania micrantha* Kunth	1	10.01	1				
PUMOL*	FACU	INTR	*Pueraria montana* (Lour.) Merr. var. *lobata* Willd.) Maesen & S. Almeida	1	51.50	1				
LACA2*	FACU	INTR	*Lantana camara* L.	1	33.00	1				
ROLA*	UPL	INTR	*Rosa laevigata* Michx.	1	22.00	1				
CASES*	FAC	INTR	*Calystegia sepium* (L.) R. Br. ssp. *sepium* (L.) R. Br.	1	40.22	1				
IPPU2*	UPL	INTR	*Ipomoea purpurea* (L.) Roth	1	49.20	1				
IPWR*	FACW	INTR	*Ipomoea wrightii* A. Gray	1	30.03	1				
PLPOP*	FAC	INTR	*Pleopeltis polypodioides* ssp. *polypodioides* (L.) Andrews & Windham	1	20.30	1				
WIFL*	UPL	INTR	*Wisteria floribunda* (Willd.) Dc.	1	20.20	1				
WISI*	UPL	INTR	*Wisteria sinensis* (Sims) Dc.	1	20.20	1				
VICR*	UPL	INTR	*Vicia cracca* L.	4	20.26 ± 7.13		4			
SEVA4*	UPL	INTR	*Securigera varia* (L.) Lassen	3	26.92 ± 11.96		3			
CONVO	NA	INTR	*Convolvulus* L.	1	10.01		1			
CYLO11*	UPL	INTR	*Cynanchum louiseae* Kartesz & Gandhi	1	10.01		1			
CYRO8*	UPL	INTR	*Cynanchum rossicum* (Kleopow) Borhidi	1	20.02		1			
EUFO5*	UPL	INTR	*Euonymus fortunei* (Turcz.) Hand.-Maz.	4	15.26 ± 5.25		2	2		
**POSC3**	**FAC/FACU**	**CRYP occurrences**	***Polygonum scandens* L.**	**8**	**31.97 ± 6.69**		**2**	**6**		
HULU	FACU	CRYP occurrences	*Humulus lupulus* L.	2	25.12 ± 5.01		1	1		
SODUD*	FAC	INTR	*Solanum dulcamara* L. var. *dulcamara* L.	2	35.13 ± 14.92		1	1		
IPHE*	FAC/FACU	INTR	*Ipomoea hederacea* Jacq.	2	36.55 ± 15.25	1		1		
**CEOR7***	**FACU/UPL**	**INTR**	***Celastrus orbiculatus* Thunb.**	**7**	**19.02 ± 5.71**	**1**	**6**			
CASE13*	FAC	CRYP occurrences	*Calystegia sepium* (L.) R. Br.	3	31.51 ± 12.83	2	1			
**LOJA***	**FAC/FACU**	**INTR**	***Lonicera japonica* Thunb.**	**41**	**24.46 ± 2.48**	**35**	**4**	**2**		
**ROMU***	**FACU**	**INTR**	***Rosa multiflora* Thunb.**	**44**	**26.07 ± 2.35**	**5**	**30**	**9**		
**CAHA13***	**FAC/FACU**	**CRYP/INTR**	***Cardiospermum halicacabum* L.**	**14**	**27.88 ± 4.78**	**12**	**1**	**1**		
**SODU***	**FAC/FACU**	**INTR**	***Solanum dulcamara* L.**	**46**	**25.65 ± 2.01**	**1**	**25**	**14**	**2**	**4**
**POCO10***	**FACU**	**INTR**	***Polygonum convolvulus* L.**	**10**	**18.36 ± 3.83**	**1**	**4**	**4**		**1**

Status			Nonnative Plant Taxa	All Sampled Sites (n = 1138)		CPL (n=567)	EMU (n=214)	IPL (n=190)	XER (n=62)	WMT (n=105)
PLANTS Symbol*	Wetland Indicator Status	Native Status	Scientific Name	# Occur	Mean Importance ± Standard Error	# Occur	# Occur	# Occur	# Occur	# Occur
**COAR4***	**UPL**	**INTR**	***Convolvulus arvensis* L.**	**13**	**19.43 ± 3.71**			**8**	**2**	**3**
HUJA*	FACU	INTR	*Humulus japonicus* Siebold & Zucc.	2	10.01 ± 0.00			2		
DIOP*	UPL	INTR	*Dioscorea oppositifolia* L.	1	10.40			1		
PAQU2	FACU	ADV occurrences	*Parthenocissus quinquefolia* (L.) Planch.	1	11.00			1		
ROCA3*	UPL	INTR	*Rosa canina* L.	1	20.41				1	
VIMA*	UPL	INTR	*Vinca major* L.	1	22.40				1	
RULA*	FACU	INTR	*Rubus laciniatus* Willd.	4	15.11 ± 5.04					4
RUAR9*	FACU	INTR	*Rubus armeniacus* Focke	4	26.48 ± 6.27					4
RUID*	FACU	CRYP occurrences	*Rubus idaeus* L.	3	13.47 ± 3.37		2			1
LALA4*	UPL	INTR	*Lathyrus latifolius* L.	1	55.00					1
SOLAN	NA	INTR	*Solanum* L.	1	10.01					1
VIHI*	UPL	INTR	*Vicia hirsuta* (L.) Gray	1	10.01					1
			Graminoids							
**PAUR2***	**FAC**	**INTR**	***Paspalum urvillei* Steud.**	**9**	**26.48 ± 5.96**	**9**				
**PADI3***	**FAC**	**INTR**	***Paspalum dilatatum* Poir.**	**7**	**27.57 ± 5.71**	**7**				
**SPIN4***	**FACU**	**INTR**	***Sporobolus indicus* (L.) R. Br.**	**5**	**34.83 ± 10.99**	**5**				
PARE3*	FACW	INTR	*Panicum repens* L.	4	34.18 ± 17.92	4				
ECCO2*	FACW	INTR	*Echinochloa colona* (L.) Link	3	34.04 ± 9.29	3				
HYAM2*	OBL	INTR	*Hymenachne amplexicaulis* (Rudge) Nees	1	40.20	1				
DAAE*	UPL	INTR	*Dactyloctenium aegyptium* (L.) Willd.	2	16.31 ± 3.81	2				
ECCR2*	OBL	INTR	*Echinochloa crus-pavonis* (Kunth) Schult.	2	26.80 ± 16.70	2				
AVSA*	UPL	INTR	*Avena sativa* L.	1	32.40	1				
CAEX8	OBL	INTR	*Carex extensa* Goodenough	1	20.20	1				
DIIS*	UPL	INTR	*Digitaria ischaemum* (Schreb.) Schreb. ExMuhl.	1	21.01	1				
ERSC3	UPL	INTR	*Eragrostis scaligera* Salzm. Ex Steud.	1	30.03	1				
IMCY*	UPL	INTR	*Imperata cylindrica* (L.) P. Beauv.	1	30.22	1				
PAIN	FAC	INTR	*Parapholis incurva* (L.) C.e. Hubbard	1	25.50	1				
PHCA5*	FACU	INTR	*Phalaris canariensis* L.	1	10.10	1				
SCLA15*	FACW	INTR	*Scleria lacustris* C. Wright	1	10.05	1				
TRAE*	UPL	INTR	*Triticum aestivum* L.	1	20.11	1				

Status			Nonnative Plant Taxa	All Sampled Sites (n = 1138)		CPL (n=567)	EMU (n=214)	IPL (n=190)	XER (n=62)	WMT (n=105)
PLANTS Symbol*	Wetland Indicator Status	Native Status	Scientific Name	# Occur	Mean Importance ± Standard Error	# Occur	# Occur	# Occur	# Occur	# Occur
ZEMA*	UPL	INTR	*Zea mays* L.	1	30.11	1				
JUAR4	OBL	ADV occurrences	*Juncus articulatus* L.	2	15.04 ± 5.03	1		1		
**SEFA***	**FACU/UPL**	**INTR**	***Setaria faberi* Herrm.**	**9**	**25.28 ± 5.16**	**1**		**8**		
**SOHA***	**FACU**	**INTR**	***Sorghum halepense* (L.) Pers.**	**9**	**19.91 ± 5.79**	**6**		**3**		
**PANO2***	**FAC/FACU**	**INTR/CRYP**	***Paspalum notatum* Flueggé**	**6**	**39.07 ± 11.11**	**5**		**1**		
**POMO5***	**FACW**	**INTR**	***Polypogon monspeliensis* (L.) Desf.**	**18**	**24.93 ± 3.75**	**3**		**2**	**13**	
**LOPE***	**FAC/FACU**	**INTR**	***Lolium perenne* L.**	**6**	**22.96 ± 4.43**	**3**		**1**	**2**	
ARDO4*	FACW/FAC	INTR	*Arundo donax* L.	3	36.10 ± 13.05	2			1	
**CYDA***	**FACU**	**INTR**	***Cynodon dactylon* (L.) Pers.**	**18**	**28.22 ± 4.07**	**14**			**4**	
ARHI3*	FAC	INTR	*Arthraxon hispidus* (Thunb.) Makino	3	24.57 ± 14.47	2	1			
**MIVI***	**FAC**	**INTR**	***Microstegium vimineum* (Trin.) A. Camus**	**20**	**34.87 ± 3.97**	**12**	**6**	**2**		
**AGCA5***	**FAC**	**INTR**	***Agrostis capillaris* L.**	**5**	**18.33 ± 6.05**		**5**			
CAMU7	FAC	INTR	*Carex muricata* L.	1	10.01		1			
CAOV8	FACW/FAC	ADV occurrences	*Carex ovalis* Goodenough	1	30.12		1			
CYES*	FACW	CRYP/INTR	*Cyperus esculentus* L.	4	26.26 ± 10.55		1	3		
SEPU8*	FAC/FACU	INTR	*Setaria pumila* (Poir.) Roem. & Schult.	3	30.21 ± 11.69	1		2		
**SEPUP2***	**FAC/FACU**	**INTR**	***Setaria pumila* ssp. *pumila* (Poir.) Roem. & Schult.**	**7**	**15.37 ± 3.56**	**2**	**1**	**4**		
**PHAU7***	**FACW**	**INTR**	***Phragmites australis* (Cav.) Trin. Ex Steud.**	**108**	**45.23 ± 2.93**	**82**	**12**	**9**	**5**	
**ECCR***	**FACW/FAC**	**INTR**	***Echinochloa crus-gall* i (L.) P. Beauv.**	**21**	**26.28 ± 3.83**	**2**	**5**	**12**	**2**	
**DISA***	**FACU**	**INTR**	***Digitaria sanguinalis* (L.) Scop.**	**7**	**29.89 ± 5.97**	**1**	**1**	**1**	**4**	
**PHAR3***	**OBL/FACW**	**CRYP**	***Phalaris arundinacea* L.**	**166**	**43.60 ± 2.08**	**4**	**52**	**90**	**2**	**18**
**ELRE4***	**FAC/FACU**	**INTR**	***Elymus repens* (L.) Gould**	**43**	**23.60 ± 2.30**	**1**	**4**	**33**	**2**	**3**
**SCAR7***	**FAC/FACU**	**INTR**	***Schedonorus arundinaceus* (Schreb.) Dumort.**	**15**	**28.40 ± 4.60**	**2**	**4**	**1**	**1**	**7**
**AGST2***	**FACW/FAC**	**INTR**	***Agrostis stolonifera* L.**	**25**	**31.50 ± 4.35**	**2**	**5**	**6**		**12**
PUDI	OBL/FACW	INTR	*Puccinellia distans* (Jacq.) Parl.	4	25.27 ± 9.46	1	1			2
**HOLA***	**FAC/FACU**	**INTR**	***Holcus lanatus* L.**	**15**	**23.63 ± 4.33**	**1**	**6**		**1**	**7**
**FERU2***	**FAC/FACU**	**CRYP occurrences**	***Festuca rubra* L.**	**15**	**28.81 ± 3.85**	**4**	**4**			**7**
**ANOD***	**FACU**	**INTR**	***Anthoxanthum odoratum* L.**	**11**	**13.72 ± 1.53**	**1**	**8**			**2**
**POTR2***	**FACW/FAC**	**INTR**	***Poa trivialis* L.**	**5**	**30.32 ± 6.42**	**2**				**3**

Status			Nonnative Plant Taxa	All Sampled Sites (n = 1138)		CPL (n=567)	EMU (n=214)	IPL (n=190)	XER (n=62)	WMT (n=105)
PLANTS Symbol*	Wetland Indicator Status	Native Status	Scientific Name	# Occur	Mean Importance ± Standard Error	# Occur	# Occur	# Occur	# Occur	# Occur
POAN*	FAC/FACU	INTR	*Poa annua* L.	3	14.07 ± 3.92	1				2
**POPR***	**FAC/FACU**	**INTR**	***Poa pratensis* L.**	**116**	**29.10 ± 1.68**		**15**	**59**	**9**	**33**
**BRIN2***	**FAC/FACU/UPL**	**CRYP/INTR**	***Bromus inermis* Leyss.**	**50**	**23.17 ± 2.26**		**1**	**35**	**4**	**10**
**PHPR3***	**FAC/FACU**	**INTR**	***Phleum pratense* L.**	**46**	**26.35 ± 2.55**		**10**	**10**	**4**	**22**
**AGGI2***	**FACW/FAC**	**INTR**	***Agrostis gigantea* Roth**	**28**	**24.23 ± 3.14**		**10**	**10**		**8**
**DAGL***	**FACU**	**INTR**	***Dactylis glomerata* L.**	**12**	**24.86 ± 5.14**		**3**	**4**	**1**	**4**
POPRP2*	FAC/FACU	INTR	*Poa pratensis* L. ssp. *pratensis* L.	3	40.40 ± 3.70		1		2	
BRRA2*	UPL	INTR	*Bromus racemosus* L.	2	15.02 ± 5.01		1			1
**BRTE***	**UPL**	**INTR**	***Bromus tectorum* L.**	**22**	**27.15 ± 3.41**			**9**	**6**	**7**
**POPA2***	**FACW/FAC**	**CRYP occurrences**	***Poa palustris* L.**	**17**	**34.69 ± 4.41**			**3**	**1**	**13**
**BRAR5***	**FACU/UPL**	**INTR**	***Bromus arvensis* L.**	**13**	**20.91 ± 5.71**			**9**	**2**	**2**
**ALAR**	**FACW/FAC**	**INTR**	***Alopecurus arundinaceus* Poir.**	**8**	**35.10 ± 6.61**			**5**	**1**	**2**
**ALGE2**	**OBL**	**CRYP/INTR**	***Alopecurus geniculatus* L.**	**6**	**38.82 ± 6.80**			**3**		**3**
POCO*	FACU	INTR	*Poa compressa* L.	3	10.01 ± 0.00			2		1
SEVI4*	UPL	INTR	*Setaria viridis* (L.) P. Beauv.	4	20.77 ± 5.51			4		
SETAR	NA	INTR	*Setaria* P. Beauv.	1	52.51			1		
JUCO	FACW	INTR	*Juncus compressus* Jacq.	1	10.10			1		
THPO7*	UPL	INTR	*Thinopyrum ponticum* (Podp.) Z.-W. Liu & R.-C. Wang	4	27.91 ± 8.82			2	2	
AGCR*	UPL	INTR	*Agropyron cristatum* (L.) Gaertn.	2	38.21 ± 13.19			1	1	
**ALPR3***	**FACW/FAC**	**INTR**	***Alopecurus pratensis* L.**	**6**	**49.70 ± 8.38**				**2**	**4**
BRHO2*	FACU	INTR	*Bromus hordeaceus* L.	2	40.22 ± 20.20				1	1
**HOMAG***	**FAC**	**INTR**	***Hordeum marinum* Huds. ssp. *gussonianum* (Parl.) Thell.**	**5**	**36.22 ± 3.96**				**5**	
**LOPEM2***	**FAC**	**INTR**	***Lolium perenne* L. ssp. *multiflorum* (Lam.) Husnot**	**5**	**32.20 ± 5.93**				**5**	
AGAV*	FACW	INTR	*Agrostis avenacea* J.f. Gmel.	2	34.86 ± 24.85				2	
CRVA2	OBL	INTR	*Crypsis vaginiflora* (Forssk.) Opiz	2	52.07 ± 11.94				2	
COJU2*	FACU	INTR	*Cortaderia jubata* (Lem.) Stapf	1	34.80				1	
CRSC	OBL	INTR	*Crypsis schoenoides* (L.) Lam.	1	40.04				1	
HACY	FACW	INTR	*Hainardia cylindric* a (Willd.) Greuter	1	56.30				1	
HOMA2*	FAC	INTR	*Hordeum marinum* Huds.	1	10.01				1	
HOMU*	FACU	INTR	*Hordeum murinum* L.	1	10.01				1	

Status			Nonnative Plant Taxa	All Sampled Sites (n = 1138)		CPL (n=567)	EMU (n=214)	IPL (n=190)	XER (n=62)	WMT (n=105)
PLANTS Symbol*	Wetland Indicator Status	Native Status	Scientific Name	# Occur	Mean Importance ± Standard Error	# Occur	# Occur	# Occur	# Occur	# Occur
POBU*	UPL	INTR	*Poa bulbosa* L.	1	50.32				1	
THIN6*	UPL	INTR	*Thinopyrum intermedium* (Host) Barkworth & D.r. Dewey	1	51.12 ± 0.00				1	
SCPR4	FACU	INTR	*Schedonorus pratensis* (Huds.) P. Beauv.	2	36.51 ± 16.30					2
AGEX	FACW	ADV occurrences	*Agrostis exarat* a Trin.	1	10.01					1
AICA*	FACU	INTR	*Aira caryophyllea* L.	1	30.03					1
APIN	UPL	INTR	*Apera interrupta* (L.) P. Beauv.	1	31.61					1
CYCR	FACU	INTR	*Cynosurus cristatus* L.	1	10.20					1
FEBR7	FACU	INTR	*Festuca brevipila* Tracey	1	31.40					1
JUEFE2	FACW	INTR	*Juncus effusus* L. var. *effusus* L.	1	47.00					1
PHAQ*	FACU	INTR	*Phalaris aquatica* L.	1	50.23					1
VUMY*	FACU	INTR	*Vulpia myuros* (L.) C.c. Gmel.	1	30.21					1
			Forbs							
**ALPH***	**OBL**	**INTR**	***Alternanthera philoxeroides* (Mart.) Griseb.**	**23**	**38.52 ± 4.33**	**23**				
**MUKE***	**OBL**	**INTR**	***Murdannia keisak* (Hassk.) Hand.-Maz.**	**9**	**40.78 ± 8.14**	**9**				
**LUPE6***	**OBL**	**INTR**	***Ludwigia peruviana* (L.) H. Hara**	**7**	**30.84 ± 9.80**	**7**				
**VEBR2***	**UPL**	**INTR**	***Verbena brasiliensis* Vell.**	**5**	**52.20 ± 0.67**	**5**				
**ATPA4**	**FACW**	**INTR**	***Atriplex patula L.***	**5**	**22.15 ± 4.95**	**5**				
**CYLE7**	**FAC**	**INTR**	***Cyclospermum leptophyllum* (Pers.) Sprague Ex Britton & P. Wilson**	**5**	**30.49 ± 7.29**	**5**				
**SAMI7***	**OBL**	**INTR**	***Salvinia minima* Baker**	**5**	**44.07 ± 9.60**	**5**				
CODI5*	FACW	INTR	*Commelina diffusa* Burm. F.	4	25.26 ± 6.48	4				
EICR*	OBL	INTR	*Eichhornia crassipes* (Mart.) Solms	4	33.03 ± 13.14	4				
**BAHY***	**FACW/FAC**	**INTR**	***Bassia hyssopifolia* (Pall.) Kuntz**	**7**	**43.11 ± 5.01**	**3**			**4**	
MIPE3	FACW	ADV occurrences	*Mitreola petiolata* (J.f. Gmel.) Torr. & A. Gray	3	16.68 ± 6.67	3				
MYAQ2*	OBL	INTR	*Myriophyllum aquaticum* (Vell.) Verdc.	3	24.54 ± 14.49	3				
RASA*	FAC	INTR	*Ranunculus sardous* Crantz	3	37.45 ± 8.56	3				
CAFR6*	UPL	INTR	*Callisia fragrans* (Lindl.) Woodson	2	40.42 ± 0.20	2				
GAPE2	FACU	INTR	*Gamochaeta pensylvanica* (Willd.) Cabrera	2	20.02 ± 0.00	2				
POPE10*	FAC	INTR	*Polygonum perfoliatum* L.	2	20.16 ± 9.96	2				
YOJA*	FACU	INTR	*Youngia japonica* (L.) Dc.	2	15.02 ± 5.01	2				

Status			Nonnative Plant Taxa	All Sampled Sites (n = 1138)		CPL (n=567)	EMU (n=214)	IPL (n=190)	XER (n=62)	WMT (n=105)
PLANTS Symbol*	Wetland Indicator Status	Native Status	Scientific Name	# Occur	Mean Importance ± Standard Error	# Occur	# Occur	# Occur	# Occur	# Occur
ALFL*	UPL	INTR	*Alternanthera flavescens* Kunth	1	20.20	1				
CAAN4	UPL	CRYP	*Capsicum annuum* L.	1	10.20	1				
MECO4*	FAC	INTR	*Melochia corchorifolia* L.	1	30.03	1				
POCU6*	UPL	INTR	*Polygonum cuspidatum* Siebold & Zucc.	1	10.10	1				
SEPU7*	FAC	INTR	*Sesbania punicea* (Cav.) Benth.	1	10.10	1				
AESCH	NA	CRYP	*Aeschynomene* L.	1	10.10	1				
ALSA2*	UPL	INTR	*Allium sativum* L.	1	40.04	1				
AMPA*	FACU	ADV occurrences	*Amaranthus palmeri* S. Watson	1	10.20	1				
ARHY*	UPL	INTR	*Arachis hypogaea* L.	1	10.10	1				
CAPA11	FACW	INTR	*Caperonia palustris* (L.) A. St.-Hil.	1	50.24	1				
COESA*	FACW	INTR	*Colocasia esculenta* (L.) Schott var. *antiquorum* (Schott) Hubbard & Rehder	1	50.70	1				
COCO3*	FAC	INTR	*Commelina communis* L.	1	42.50	1				
ECPR*	FACW/FAC	ADV occurrences	*Eclipta prostrata* (L.) L.	1	10.50	1				
GLMA4*	UPL	INTR	*Glycine max* (L.) Merr.	1	20.02	1				
HYMU2	FAC	INTR	*Hyptis mutabilis* (A. Rich.) Briq.	1	10.01	1				
LUGRG2*	OBL	INTR	*Ludwigia grandiflora* (Michx.) ssp. *grandiflora* (Michx.) Greuter & Burdet	1	10.10	1				
MATO3	FACW	INTR	*Macrothelypteris torresiana* (Gaudich.) Ching	1	20.20	1				
MIHI2	FACU	INTR	*Mitracarpus hirtus* (L.) Dc.	1	10.01	1				
MOOD3	UPL	INTR	*Morrenia odorata* (Hook. & Arn.) Lindl.	1	10.01	1				
OLCO	FAC	INTR	*Oldenlandia corymbosa* L.	1	10.01	1				
OXDEC	UPL	INTR	*Oxalis debilis* Kunth var. *corymbosa* (Dc.) Lourteig	1	40.40	1				
THDE4	FACW	INTR	*Thelypteris dentata* (Forssk.) E.p. St. John	1	20.02	1				
**POCEL***	**FAC**	**INTR**	***Polygonum cespitosum* Blume var. *longisetum* (Bruijn) A.N. Steward**	**12**	**25.84 ± 4.45**	**9**	**3**			
**LECU***	**FACU**	**INTR**	***Lespedeza cuneata* (Dum. Cours.) G. Don**	**6**	**11.74 ± 1.69**	**5**	**1**			
MYAQ*	FACW/FAC	INTR	*Myosoton aquaticum* (L.) Moench	3	30.22 ± 5.81	2	1			
SAOF4*	FACU	INTR	*Saponaria officinalis* L.	3	26.88 ± 8.95	1	2			
**SISP***	**FACU**	**ADV occurrences**	***Sida spinosa* L.**	**8**	**42.94 ± 5.19**	**7**		**1**		

Status			Nonnative Plant Taxa	All Sampled Sites (n = 1138)		CPL (n=567)	EMU (n=214)	IPL (n=190)	XER (n=62)	WMT (n=105)
PLANTS Symbol*	Wetland Indicator Status	Native Status	Scientific Name	# Occur	Mean Importance ± Standard Error	# Occur	# Occur	# Occur	# Occur	# Occur
CHAM	FAC/FACU	INTR	*Chenopodium ambrosioides* L.	4	17.66 ± 4.82	2		2		
AMAR2*	FACU	CRYP occurrences	*Ambrosia artemisiifolia* L.	35	23.45 ± 2.64	14	3	18		
HEIN	FACW/FAC	INTR	*Heliotropium indicum* L.	7	20.12 ± 4.41	4	1	2		
URDI*	FACW/FAC/FACU	INTR/CRYP occurrences	*Urtica dioica* L.	9	19.09 ± 4.95	3	5	1		
POCE4*	FACW/FACU	INTR	*Polygonum cespitosum* Blume, Nom. Inq.	7	15.82 ± 2.98	2	3	2		
SONCH	NA	INTR	*Sonchus* L.	5	10.11 ± 0.10	2	2	1		
POHY	OBL	INTR	*Polygonum hydropiper* L.	14	28.46 ± 5.74	1	12	1		
ALPE4*	FAC/FACU	INTR	*Alliaria petiolata* (M. Bieb.) Cavara & Grande	14	22.22 ± 3.68	1	7	6		
TYAN*	OBL	INTR/CRYP	*Typha angustifolia* L.	67	41.28 ± 3.20	18	12	31	4	2
RUCR*	FAC	INTR	*Rumex crispus* L.	96	24.20 ± 1.46	7	7	50	14	18
TRRE3*	FAC/FACU	INTR	*Trifolium repens* L.	32	25.50 ± 2.77	4	3	6	6	13
TAOF*	FACU	INTR	*Taraxacum officinale* F.h. Wigg.	114	27.07 ± 1.48	1	15	33	10	55
PLMA2*	FAC/FACU	INTR	*Plantago major* L.	24	17.75 ± 2.34	2	3	7	1	11
POPE3*	FACW/FAC	INTR	*Polygonum persicaria* L.	17	30.02 ± 4.58	2	8	4	3	
RUAC3*	FACU/UPL	INTR	*Rumex acetosella* L.	7	10.04 ± 0.02	2	2			3
SOAS*	FAC/FACU	INTR	*Sonchus asper* (L.) Hill	7	20.14 ± 4.39	2			3	2
RUCO2*	FACW	INTR	*Rumex conglomeratus* Murray	4	20.30 ± 10.23	2			1	1
DUIN	FACU/UPL	INTR	*Duchesnea indica* (Andrews) Focke	4	10.03 ± 0.02	1	2			1
STME2*	FACU	INTR	*Stellaria media* (L.) Vill.	4	10.01 ± 0.00	1	1	1		1
ARVU*	UPL	INTR	*Artemisia vulgaris* L.	2	11.01 ± 1.00	1	1			
HYVI6	NA	ADV occurrences	*Hypericum virgatum* Lam.	2	15.02 ± 5.01	1	1			
MELU*	FACU/UPL	INTR	*Medicago lupulina* L.	10	22.70 ± 4.36	1		5		4
ANAR6*	UPL	INTR	*Anthemis arvensis* L.	3	17.04 ± 3.53	1		1	1	
POOL*	FAC/FACU	INTR	*Portulaca oleracea* L.	3	33.59 ± 12.11	1		1	1	
VEAR*	FACU/UPL	INTR	*Veronica arvensis* L.	2	15.02 ± 5.01	1		1		
AMAL*	FACU	ADV occurrences	*Amaranthus albus* L.	4	35.59 ± 9.87	1			2	1
ASOF	FACU	INTR	*Asparagus officinalis* L.	2	15.02 ± 5.01	1			1	
GATE2*	FACU	INTR	*Galeopsis tetrahit* L.	5	18.27 ± 3.93		5			
SUMA	OBL	CRYP	*Suaeda maritima* (L.) Dumort.	3	37.18 ± 7.06		3			

Status			Nonnative Plant Taxa	All Sampled Sites (n = 1138)		CPL (n=567)	EMU (n=214)	IPL (n=190)	XER (n=62)	WMT (n=105)
PLANTS Symbol*	Wetland Indicator Status	Native Status	Scientific Name	# Occur	Mean Importance ± Standard Error	# Occur	# Occur	# Occur	# Occur	# Occur
CIPA6*	FACW	INTR	*Cirsium palustre* (L.) Scop.	3	20.08 ± 5.81		3			
SEDUM	NA	INTR	*Sedum* L.	3	16.81 ± 3.40		3			
TUFA*	FACU	INTR	*Tussilago farfara* L.	3	30.61 ± 10.30		3			
ACCA4	OBL	INTR	*Acorus calamus* L.	2	32.76 ± 22.26		2			
ALVI*	FACU	INTR	*Allium vineale* L.	2	20.02 ± 10.01		2			
EPHI*	FACW	INTR	*Epilobium hirsutum* L.	2	41.62 ± 1.49		2			
EPPA5	NA	INTR	*Epilobium parviflorum* Schreb.	2	15.02 ± 5.01		2			
HILA8*	UPL	INTR	*Hieracium lachenalii* C.c. Gmel.	2	30.03 ± 10.01		2			
STGR*	UPL	INTR	*Stellaria graminea* L.	2	15.16 ± 5.15		2			
AMBL2	FACU	INTR	*Amaranthus blitum* L.	1	10.01		1			
ANMO2	UPL	INTR	*Anagallis monelli* L.	1	50.80		1			
CAHI3*	FACU	INTR	*Cardamine hirsuta* L.	1	10.01		1			
CEJA*	FACU	INTR	*Centaurea jacea* L.	1	10.01		1			
CESTM	UPL	INTR	*Centaurea stoebe* L. ssp. *micranthos* (Gugler) Hayek	1	20.02		1			
CEFO2*	FACU	INTR	*Cerastium fontanum* Baumg.	1	20.02		1			
	NA	INTR	Forb, introduced	1	10.01		1			
GAHI	UPL	ADV occurrences	*Galium hispidulum* Michx.	1	10.01		1			
GAMO*	NA	INTR	*Galium mollugo* L.	1	40.41		1			
GEUR*	UPL	INTR	*Geum urbanum* L.	1	50.62		1			
HEMA3*	FACU	INTR	*Hesperis matronalis* L.	1	10.01		1			
HICA10*	UPL	INTR	*Hieracium caespitosum* Dumort.	1	20.11		1			
HIPI*	UPL	INTR	*Hieracium pilosella* L.	1	10.01		1			
HYMO6*	OBL	INTR	*Hydrocharis morsus-ranae* L.	1	58.30		1			
LUGR9*	OBL	INTR	*Ludwigia grandiflora* (Michx.) Greuter&Burdet	1	30.03		1			
LYEU	OBL	INTR	*Lycopus europaeus* L.	1	10.01		1			
LYRU*	OBL	ADV occurrences	*Lycopus rubellus* Moench	1	40.04		1			
MEPU*	NA	INTR	*Mentha pulegium* L.	1	10.01		1			
MESP3*	FACW	INTR	*Mentha spicata* L.	1	11.00		1			
NAMI2*	OBL	INTR	*Nasturtium microphyllum* Boenn. Ex Rchb.	1	20.11		1			
SPMA10	FACU	INTR	*Spergularia maritima* (All.) Chiov.	1	30.32		1			

Status			Nonnative Plant Taxa	All Sampled Sites (n = 1138)		CPL (n=567)	EMU (n=214)	IPL (n=190)	XER (n=62)	WMT (n=105)
PLANTS Symbol*	Wetland Indicator Status	Native Status	Scientific Name	# Occur	Mean Importance ± Standard Error	# Occur	# Occur	# Occur	# Occur	# Occur
SYOF*	UPL	INTR	*Symphytum officinale* L.	1	30.21		1			
TAOFO*	FACU	INTR	*Taraxacum officinale* ssp. *officinale* F.h. Wigg.	1	10.01		1			
TRAU2	UPL	INTR	*Trifolium aureum* Pollich	1	10.01		1			
**TYGL***	**OBL**	**INTR**	***Typha ×glauca* Godr. (Pro Sp.)**	**49**	**46.78 ± 3.44**			**49**		
**LYNU***	**FACW**	**INTR**	***Lysimachia nummularia* L.**	**21**	**35.30 ± 4.91**		**13**	**8**		
**RAAC3***	**FAC**	**INTR**	***Ranunculus acris* L.**	**11**	**20.04 ± 4.87**		**10**	**1**		
**DACA6***	**UPL**	**INTR**	***Daucus carota* L.**	**7**	**24.54 ± 4.57**		**3**	**4**		
EPHE*	UPL	INTR	*Epipactis helleborine* (L.) Crantz	3	30.03 ± 5.78		2	1		
**TARAX***	**NA**	**INTR**	***Taraxacum* F.H. Wigg.**	**6**	**21.69 ± 6.55**		**5**	**1**		
**BAVU***	**FAC/FACU**	**INTR**	***Barbarea vulgaris* W.t. Aiton**	**5**	**14.01 ± 2.45**		**4**	**1**		
**STPA**	**OBL**	**INTR**	***Stachys palustris* L.**	**6**	**16.76 ± 6.73**		**1**	**5**		
TRIFO	NA	INTR	*Trifolium* L.	4	13.14 ± 2.37		1	3		
CAAC*	UPL	INTR	*Carduus acanthoides* L.	3	12.51 ± 2.50		1	2		
MESA*	FACU/UPL	INTR	*Medicago sativa* L.	3	20.91 ± 5.10		1	2		
MYSP2*	OBL	INTR	*Myriophyllum spicatum* L.	3	16.42 ± 6.17		1	2		
POCR3*	OBL	INTR	*Potamogeton crispus* L.	2	10.76 ± 0.75		1	1		
ROSY*	OBL	INTR	*Rorippa sylvestris* (L.) Besser	2	15.06 ± 4.96		1	1		
RUCRC*	FAC	INTR	*Rumex crispus* L. ssp. *crispus* L.	2	35.08 ± 5.05		1	1		
MOVE*	FAC/FACU	ADV occurrences	*Mollugo verticillata* L.	4	22.87 ± 7.78		1		3	
**PRVU***	**FAC/FACU**	**CRYP occurrences**	***Prunella vulgaris* L.**	**25**	**19.28 ± 2.53**		**12**	**5**		**8**
**GLHE2***	**FACU**	**INTR**	***Glechoma hederacea* L.**	**8**	**26.80 ± 7.53**		**5**	**2**		**1**
**HYPE***	**FAC/FACU/UPL**	**INTR**	***Hypericum perforatum* L.**	**6**	**13.44 ± 2.17**		**3**	**1**		**2**
**SOAR2***	**FAC/FACU**	**INTR**	***Sonchus arvensis* L.**	**34**	**18.57 ± 1.83**		**2**	**30**		**2**
**TRPR2***	**FACU**	**INTR**	***Trifolium pratense* L.**	**16**	**18.32 ± 2.55**		**2**	**9**		**5**
ERCH9*	FACU	INTR	*Erysimum cheiranthoides* L.	4	17.59 ± 2.53		2	1		1
**ARMI2***	**FACU/UPL**	**INTR**	***Arctium minus* Bernh.**	**11**	**17.39 ± 3.87**		**4**	**2**		**5**
**IRPS***	**OBL**	**INTR**	***Iris pseudacorus* L.**	**7**	**24.92 ± 5.98**		**4**	**1**		**2**
**VETH***	**FACU/UPL**	**INTR**	***Verbascum thapsus* L.**	**11**	**11.18 ± 0.92**		**1**	**3**		**7**
**LYSA2***	**OBL**	**INTR**	***Lythrum salicaria* L.**	**22**	**32.14 ± 4.39**		**15**	**6**	**1**	
**CIAR4***	**FAC/FACU**	**INTR**	***Cirsium arvense* (L.) Scop.**	**77**	**23.02 ± 1.60**		**6**	**48**	**3**	**20**
**MEOF***	**FACU**	**INTR**	***Melilotus officinalis* (L.) Lam.**	**20**	**25.38 ± 4.21**		**2**	**9**	**6**	**3**

Status			Nonnative Plant Taxa	All Sampled Sites (n = 1138)		CPL (n=567)	EMU (n=214)	IPL (n=190)	XER (n=62)	WMT (n=105)
PLANTS Symbol*	Wetland Indicator Status	Native Status	Scientific Name	# Occur	Mean Importance ± Standard Error	# Occur	# Occur	# Occur	# Occur	# Occur
**CIVU***	**FACU/UPL**	**INTR**	***Cirsium vulgare* (Savi) Ten.**	**25**	**16.58 ± 2.50**		**2**	**5**	**5**	**13**
**ACMI2***	**FACU**	**CRYP occurrences**	***Achillea millefolium* L.**	**21**	**30.65 ± 3.08**		**2**	**1**	**1**	**17**
**VEAN2***	**OBL**	**INTR**	***Veronica anagallis-aquatica* L.**	**7**	**20.11 ± 6.60**		**1**	**4**	**1**	**1**
**POAV***	**FACW/FAC/FACU**	**INTR**	***Polygonum aviculare* L.**	**12**	**27.14 ± 4.94**		**1**	**3**	**1**	**7**
**PORE5***	**UPL**	**INTR**	***Potentilla recta* L.**	**6**	**20.35 ± 6.54**		**1**	**1**	**1**	**3**
PASA2*	UPL	INTR	*Pastinaca sativa* L.	4	17.56 ± 2.52		1	1		2
**LOCO6***	**FAC/FACU**	**INTR**	***Lotus corniculatus* L.**	**11**	**23.89 ± 3.95**		**1**		**4**	**6**
**PLLA***	**FAC/FACU/UPL**	**INTR**	***Plantago lanceolata* L.**	**5**	**20.99 ± 8.70**		**2**		**1**	**2**
RUOB*	FAC/FACU	INTR	*Rumex obtusifolius* L.	4	15.13 ± 5.06		2		1	1
**RARE3***	**FAC**	**INTR**	***Ranunculus repens* L.**	**7**	**27.47 ± 6.15**		**4**			**3**
**VEOF2**	**FACU/UPL**	**INTR**	***Veronica officinalis* L.**	**5**	**18.08 ± 3.75**		**4**			**1**
**VESE***	**FAC**	**INTR/CRYP**	***Veronica serpyllifolia* L.**	**8**	**15.03 ± 3.78**		**3**			**5**
**TRHY***	**FAC/FACU**	**INTR**	***Trifolium hybridum* L.**	**6**	**17.08 ± 5.26**		**3**			**3**
**LEVU***	**FACU/UPL**	**INTR**	***Leucanthemum vulgare* Lam.**	**5**	**14.06 ± 2.46**		**3**			**2**
MYSC*	OBL/FACW	INTR	*Myosotis scorpioides* L.	3	29.78 ± 5.51		2			1
HIAU*	UPL	INTR	*Hieracium aurantiacum* L.	2	20.02 ± 0.00		1			1
NAOF*	OBL	INTR	*Nasturtium officinale* W.t. Aiton	2	15.11 ± 5.01		1			1
**LELA2***	**FACW/FAC**	**INTR**	***Lepidium latifolium* L.**	**17**	**28.84 ± 3.74**			**2**	**15**	
RUST4	FACW	INTR	*Rumex stenophyllus* Ledeb.	4	25.41 ± 9.75			2	2	
AMRE*	FACU	ADV/CRYP occurrences	*Amaranthus retroflexus* L.	3	37.08 ± 3.53			2	1	
**ATPR**	**FACW/FAC**	**ADV occurrences**	***Atriplex prostrata* Bouchér Ex Dc.**	**19**	**27.58 ± 3.77**			**1**	**18**	
ABTH*	UPL	INTR	*Abutilon theophrasti* Medik.	3	26.90 ± 8.87			1	2	
AMBL*	FACW/FAC	INTR	*Amaranthus blitoides* S. Watson	2	26.06 ± 16.05			1	1	
**LASE**	**FAC/FACU**	**INTR**	***Lactuca serriola* L.**	**27**	**22.64 ± 3.05**			**11**	**11**	**5**
**CHAL7***	**FACU**	**INTR/CRYP occurrences**	***Chenopodium album* L.**	**12**	**15.19 ± 1.97**			**9**	**1**	**2**
**BASC5***	**FAC/FACU**	**INTR**	***Bassia scoparia* (L.) A.j. Scott**	**14**	**37.59 ± 5.54**			**8**	**4**	**2**
**TRDU***	**UPL**	**INTR**	***Tragopogon dubius* Scop.**	**16**	**19.56 ± 3.33**			**8**	**2**	**6**
**CYOF***	**FACU**	**INTR**	***Cynoglossum officinale* L.**	**11**	**18.30 ± 3.58**			**5**	**1**	**5**
**SOOL***	**UPL**	**INTR**	***Sonchus oleraceus* L.**	**17**	**21.24 ± 4.26**			**4**	**10**	**3**
**POAR11***	**UPL**	**INTR**	***Polygonum arenastrum* Jord. Ex Boreau**	**10**	**27.05 ± 4.75**			**2**	**6**	**2**

Status			Nonnative Plant Taxa	All Sampled Sites (n = 1138)		CPL (n=567)	EMU (n=214)	IPL (n=190)	XER (n=62)	WMT (n=105)
PLANTS Symbol*	Wetland Indicator Status	Native Status	Scientific Name	# Occur	Mean Importance ± Standard Error	# Occur	# Occur	# Occur	# Occur	# Occur
**COMA2***	**FACW/FAC**	**INTR**	***Conium maculatum* L.**	**6**	**32.02 ± 7.16**			**2**	**3**	**1**
CANU4*	FACU/UPL	INTR	*Carduus nutans* L.	4	12.51 ± 2.50			1	1	2
SATR12*	FACU	INTR	*Salsola tragus* L.	4	17.59 ± 7.52			1	1	2
CABU2*	FACU	INTR	*Capsella bursa-pastoris* (L.) Medik.	4	15.04 ± 2.90			1		3
PONO3	FAC/FACU	ADV occurrences	*Potentilla norvegica* L.	3	20.02 ± 10.01			1		2
BRRA*	FACU/UPL	INTR	*Brassica rapa* L.	2	20.21 ± 10.10			1		1
**THAR5***	**FACU/UPL**	**INTR**	***Thlaspi arvense* L.**	**9**	**22.49 ± 5.81**			**6**		**3**
**DESO2***	**UPL**	**INTR**	***Descurainia sophia* (L.) Webb Ex Prantl**	**6**	**16.72 ± 4.22**			**2**		**4**
**MELIL**	**NA**	**INTR**	***Melilotus* Mill.**	**6**	**13.76 ± 2.02**			**6**		
ARBI2*	FACU	INTR	*Artemisia biennis* Willd.	4	10.06 ± 0.03			4		
CHGL3	FAC	INTR	*Chenopodium glaucum* L.	4	12.53 ± 2.52			4		
ARAB3*	UPL	INTR	*Artemisia absinthium* L.	3	13.35 ± 3.34			3		
EUES*	UPL	INTR	*Euphorbia esula* L.	3	13.51 ± 3.50			3		
AMARE*	FACU	INTR	*Ambrosia artemisiifolia* L. var. *elatior* (L.) Descourtils	2	47.27 ± 2.97			2		
BIFR	FACW	ADV occurrences	*Bidens frondosa* L.	2	15.02 ± 5.01			2		
KUST*	FACU	INTR	*Kummerowia stipulacea* (Maxim.) Makino	2	10.06 ± 0.04			2		
PEFR4*	FAC	INTR	*Perilla frutescens* (L.) Britton	2	15.11 ± 5.01			2		
SALSO	NA	INTR	*Salsola* L.	2	25.03 ± 5.01			2		
SAKA*	FACU	INTR	*Salsola kali* L.	2	30.30 ± 10.10			2		
SILA21*	UPL	INTR	*Silene latifolia* Poir.	2	25.03 ± 15.02			2		
TAVU*	FACU	INTR	*Tanacetum vulgare* L.	2	10.06 ± 0.04			2		
TRFR2	FAC	INTR	*Trifolium fragiferum* L.	2	25.41 ± 15.40			2		
ARLA3*	UPL	INTR	*Arctium lappa* L.	1	10.01			1		
BIBI7*	FAC	ADV occurrences	*Bidens bipinnata* L.	1	30.07			1		
CASA3*	FACU	INTR	*Cannabis sativa* L.	1	10.01			1		
CEPU3	FACU	INTR	*Centaurium pulchellum* (Sw.) Druce	1	10.10			1		
CIIN*	FACU	INTR	*Cichorium intybus* L.	1	10.01			1		
EUDA5	UPL	INTR	*Euphorbia davidii* Subils	1	20.02			1		
GEDI*	UPL	INTR	*Geranium dissectum* L.	1	10.20			1		
HEFU*	UPL	INTR	*Hemerocallis fulva* (L.) L.	1	10.20			1		

Status			Nonnative Plant Taxa	All Sampled Sites (n = 1138)		CPL (n=567)	EMU (n=214)	IPL (n=190)	XER (n=62)	WMT (n=105)
PLANTS Symbol*	Wetland Indicator Status	Native Status	Scientific Name	# Occur	Mean Importance ± Standard Error	# Occur	# Occur	# Occur	# Occur	# Occur
LECA5*	UPL	INTR	*Lepidium campestre* (L.) W.t. Aiton	1	10.01			1		
LYVU*	FACW	INTR	*Lysimachia vulgaris* L.	1	20.21			1		
MALVA	NA	INTR	*Malva* L.	1	10.10			1		
NECA2*	FACU	INTR	*Nepeta cataria* L.	1	10.01			1		
POOR2*	FACW	INTR	*Polygonum orientale* L.	1	10.10			1		
RUAC2*	UPL	INTR	*Rumex acetosa* L.	1	10.01			1		
SIAR4*	UPL	INTR	*Sinapis arvensis* L.	1	35.60			1		
SIARA*	UPL	INTR	*Sinapis arvensis* L. ssp. *arvensis* L.	1	10.10			1		
TORIL*	NA	INTR	*Torilis* Adans.	1	10.10			1		
TOAR*	UPL	INTR	*Torilis arvensis* (Huds.) Link	1	32.21			1		
TRPE21*	UPL	INTR	*Tripleurospermum perforatum* (Mérat) M.Lainz	1	10.01			1		
**COCO7***	**OBL**	**INTR**	***Cotula coronopifolia* L.**	**9**	**32.93 ± 5.36**				**9**	
**PIEC***	**FACU**	**INTR**	***Picris echioides* L.**	**6**	**15.02 ± 2.24**				**6**	
**RASA2***	**UPL**	**INTR**	***Raphanus sativus* L.**	**5**	**22.14 ± 7.37**				**5**	
**RUPU3**	**FAC**	**INTR**	***Rumex pulcher* L.**	**5**	**10.01 ± 0.00**				**5**	
MEIN2*	FACU	INTR	*Melilotus indicus* (L.) All.	3	16.68 ± 6.67				3	
ACRE3*	UPL	INTR	*Acroptilon repens* (L.) Dc.	2	28.86 ± 18.76				2	
ATMI2	UPL	INTR	*Atriplex micrantha* Ledeb.	2	10.10 ± 0.00				2	
ANCO2*	FACU	INTR	*Anthemis cotula* L.	1	50.05				1	
CAMA*	FAC	INTR	*Cakile maritima* Scop.	1	10.01				1	
CADR*	UPL	INTR	*Cardaria draba* (L.) Desv.	1	51.51				1	
CAPY2*	UPL	INTR	*Carduus pycnocephalus* L.	1	10.01				1	
CESO3*	UPL	INTR	*Centaurea solstitialis* L.	1	10.01				1	
CESE4	UPL	INTR	*Cerastium semidecandrum* L.	1	40.08				1	
DIFU2*	FAC	INTR	*Dipsacus fullonum* L.	1	20.20				1	
LACO3*	FAC	INTR	*Lapsana communis* L.	1	10.01				1	
LEPE2	FACU	INTR	*Lepidium perfoliatum* L.	1	30.03				1	
LYTR2	OBL	INTR	*Lythrum tribracteatum* Salzm. Ex Spreng.	1	10.01				1	
MYDI	FACW	INTR	*Myosotis discolor* Pers.	1	30.31				1	
POAR5*	FAC	INTR	*Polygonum argyrocoleon* Steud. Ex Kunze	1	30.03				1	
POPE2*	FACW	ADV occurrences	*Polygonum pensylvanicum* L.	1	20.02				1	
RUKE	FACW	INTR	*Rumex kerneri* Borbás	1	10.20				1	

Status			Nonnative Plant Taxa	All Sampled Sites (n = 1138)		CPL (n=567)	EMU (n=214)	IPL (n=190)	XER (n=62)	WMT (n=105)
PLANTS Symbol*	Wetland Indicator Status	Native Status	Scientific Name	# Occur	Mean Importance ± Standard Error	# Occur	# Occur	# Occur	# Occur	# Occur
**LYHY3**	**OBL**	**INTR**	***Lythrum hyssopifolium* L.**	**7**	**38.80 ± 4.66**				**6**	**1**
ERCI6*	UPL	INTR	*Erodium cicutarium* (L.) L'hér. Ex Aiton	4	25.07 ± 5.02				2	2
ONAC*	UPL	INTR	*Onopordum acanthium* L.	3	30.09 ± 10.04				1	2
SIAL2*	FACU	INTR	*Sisymbrium altissimum* L.	2	30.12 ± 10.10				1	1
**ARGL**	**UPL**	**ADV occurrences**	***Arabis glabra* (L.) Bernh.**	**5**	**40.28 ± 7.78**					**5**
**TRDU2***	**FACU/UPL**	**INTR**	***Trifolium dubium* Sibth.**	**5**	**20.10 ± 4.52**					**5**
**DIPU***	FACU	INTR	*Digitalis purpurea* L.	3	36.94 ± 13.42					3
ERMI6*	FACU	INTR	*Erechtites minima* (Poir.) Dc.	2	25.25 ± 5.05					2
GAPA5	FACU	INTR	*Galium parisiense* L.	2	35.50 ± 15.20					2
BRNI*	UPL	INTR	*Brassica nigra* (L.) W.d.j. Koch	1	20.11					1
CAMI2*	FACU	INTR	*Camelina microcarpa* Andrz. Ex Dc.	1	10.01					1
CARU15	UPL	INTR	*Camelina rumelica* Velen.	1	10.01					1
CEGL2	FACU	INTR	*Cerastium glomeratum* Thuill.	1	30.22					1
CRTE3*	UPL	INTR	*Crepis tectorum* L.	1	20.02					1
DRNE	UPL	ADV occurrences	*Draba nemorosa* L.	1	10.10					1
HYRA3*	FACU	INTR	*Hypochaeris radicata* L.	1	10.01					1
IMGL*	FACW	INTR	*Impatiens glandulifera* Royle	1	36.11					1
LASA*	UPL	INTR	*Lactuca saligna* L.	1	56.20					1
LAAM*	UPL	INTR	*Lamium amplexicaule* L.	1	30.12					1
MADI6*	FACU	INTR	*Matricaria discoidea* Dc.	1	10.01					1
MYMU*	UPL	INTR	*Mycelis muralis* (L.) Dumort.	1	10.01					1
POLA4*	OBL/FACW	ADV occurrences	*Polygonum lapathifolium* L.	1	10.01					1
PORA3	FACW/FAC/FACU	ADV occurrences	*Polygonum ramosissimum* Michx.	1	10.01					1
SCLA6*	UPL	INTR	*Scorzonera laciniata* L.	1	10.01					1
SEJA*	FACU	INTR	*Senecio jacobaea* L.	1	30.03					1
SIGA*	UPL	INTR	*Silene gallica* L.	1	50.05					1
TRAGO	NA	INTR	*Tragopogon* L.	1	20.02					1
