# Supplementary material for: Characterizing nonnative plants in wetlands across the conterminous United States
Source: Environ Monit Assess. 2019 Jun 20;191(Suppl 1):344. doi: 10.1007/s10661-019-7317-3 (PMC6586712; doi:10.1007/s10661-019-7317-3)
Supplement: Supplementary file 2 — (PDF 122 kb) [file 10661_2019_7317_MOESM2_ESM.pdf]

Supplement 2 – Supplementary Results, Tables A through D

Journal: Environmental Monitoring and Assessment

Paper title: *Characterizing nonnative plants in wetlands across the conterminous United States*

Authors: Teresa K. Magee, Karen A. Blocksom, Alan T. Herlihy, and Amanda M. Nahlik

Corresponding Author: Teresa K. Magee, US Environmental Protection Agency, Office Research and Development, National Health Effects Laboratory, Western Ecology Division, Corvallis, Oregon

Table A Estimated wetland area (hectares) and 95% confidence intervals for the NWCA sampled population in each of four wetland types (see Table 3 in paper for definitions) within five ecoregions (see Fig. 1 in paper for definitions) spanning the conterminous US. n = the number of probability sampled sites on which estimates are based. See Fig. 2 in paper for graphical presentation of these results

	**NWCA Wetland Types**			
**Ecoregion**	Estuarine Herbaceous (EH)	Estuarine Woody (EW)	Inland Herbaceous (PRLH)	Inland Woody (PRLW)
Coastal Plains (CPL)	1,936,692 ± 418,343 (n = 220)	201,460 ± 125,561 (n =68)	1,517,794 ± 517,065 (n = 62)	8,846,131 ± 1,347,912 (n =163)
Eastern Mountains and Upper Midwest (EMU)	11,805 ± 6,573 (n =13)	no estimate (n =1)	1,522,464 ± 513,893 (n = 55)	6,541,908 ± 1,253,409 (n = 83)
Interior Plains (IPL)	0	0	1,861,081 ± 405,396	1,238,474 ± 666,268
Xeric West (XER)	63,187 ± 10,936 (n = 18)	0	415,216 ± 248,265 (n = 24)	599,753 ± 257,540 (n = 17)
Western Mountains and Valleys (WMT)	6,817 ± 3,301 (n = 7)	0	187,012 ± 87,562 (n = 46)	203,926 ± 65,693 (n = 34)

Table B Population-weighted means and 95% confidence intervals for Nonnative Plant Indicator (NNPI) metrics (see Table 1 in paper for definitions), nationally and for NWCA ecoregion (see Fig. 1 in paper for definitions), and by NWCA wetland type (see Table 3 in paper for definitions). See Fig. 4 in paper for graphical presentation of these results

**NNPI Component**	**Conterminous US and NWCA Ecoregions**						**NWCA Wetland Types**			
**Metrics**	**US**	**CPL**	**EMU**	**IPL**	**XER**	**WMT**	**EH**	**EW**	**PRLH**	**PRLW**
Nonnative Richness (# nonnative species)	2.14 ± 0.25	1.30 ± 0.21	1.98 ± 0.61	4.61 ± 0.77	4.87 ± 0.88	4.97 ± 0.82	0.61 ± 0.19	0.34 ± 0.32	3.82 ± 0.32	1.81 ± 0.58
Nonnative Relative Frequency (0-100 %)	7.98 ± 0.96	6.07 ± 1.31	3.51 ± 1.20	17.37 ± 3.73	34.76 ± 4.00	12.89 ± 2.63	15.11 ± 5.60	1.81 ± 1.72	16.08 ± 2.50	4.67 ± 0.77
Nonnative Relative Cover (0-100 %)	9.34 ± 1.51	6.63 ± 1.67	4.54 ± 2.11	24.11 ± 6.29	33.46 ± 12.16	11.53 ± 3.85	17.49 ± 7.32	0.82 ± 0.77	22.04 ± 4.40	4.49 ± 1.29
See Table 4 for the number of sampled probability sites and the estimated wetland area within NWCA ecoregion and wetland types on which population-weighted means are based										

Table C Population-weighted means and 95% CIs for combined absolute cover of nonnatives within growth-habits, nationally and by NWCA ecoregion (see Fig. 1 in paper for definitions) and NWCA wetland type (see Table 3 in paper for definitions). See Fig. 5 in paper for graphical presentation of these results

	Conterminous US and NWCA Ecoregions						NWCA Wetland Types			
**Absolute Cover**	**US**	**CPL**	**EMU**	**IPL**	**XER**	**WMT**	**EH**	**EW**	**PRLH**	**PRLW**
Nonnative Forb	3.8 ± 1.03	2.02 ± 1.17	2.18 ± 1.74	13.30 ± 4.38	10.45 ± 6.67	2.12 ± 0.76	2.96 ± 2.61	0.40 ± 0.45	10.20 ± 3.38	1.95 ± 0.88
Nonnative Graminoid	4.73 ± 1.12	4.17 ± 1.48	3.82 ± 2.12	8.87 ± 3.41	4.43 ± 2.32	9.53 ± 3.52	17.60 ± 7.33	0.33 ± 0.31	9.07 ± 2.50	1.92 ± 1.09
Nonnative Vine	0.36 ± 0.14	0.55 ± 0.25	0.17 ± 0.16	0.20 ± 0.11	0.16 ± 0.22	0.04 ± 0.04	0.13 ± 0.20	< 0.001	0.51 ± 0.50	0.35 ± 0.13
Nonnative Tree/Shrub	1.02 ± 0.29	1.48 ± 0.56	0.24 ± 0.16	0.47 ± 0.25	3.56 ± 1.23	0.03 ± 0.03	0.02 ± 0.03	0.26 ± 0.42	0.42 ± 0.27	1.34 ± 0.41

See Table 4 for the number of sampled probability sites and the estimated wetland area within NWCA ecoregion and wetland types on which population-weighted means are based

Table D Population-weighted means and 95% confidence intervals for human-mediated disturbance metrics, presented nationally and for NWCA ecoregion (see Fig. 1 in paper for definitions), and NWCA wetland type (see Table 3 in paper for definitions). See Fig. 6 in paper for graphical presentation of these results

	Conterminous US and NWCA Ecoregions						NWCA Wetland Types			
**Human-Mediated Disturbance**	**US**	**CPL**	**EMU**	**IPL**	**XER**	**WMT**	**EH**	**EW**	**PRLH**	**PRLW**
Site-level Disturbance Index (SDI) (0-100)	3.98 ± 0.48	3.58 ± 0.77	3.25 ± 0.72	4.46 ± 0.92	10.96 ± 1.17	8.48 ± 1.71	1.62 ± 0.79	2.45 ± 0.83	6.15 ± 1.16	3.58 ± 0.53
2006 Agricultural Land (% in surrounding 1000m)	18.98 ± 2.24	19.47 ± 3.62	10.02 ± 3.73	43.23 ± 5.96	15.65 ± 3.69	5.34 ± 5.30	0.69 ± 0.61	0.18 ± 0.18	31.78 ± 5.01	17.28 ± 2.95
2006 Developed Land (% in surrounding 1000m)	4.91 ± 0.93	4.15 ± 0.88	5.74 ± 1.95	4.43 ± 0.69	9.75 ± 11.33	2.64 ± 1.14	2.00 ± 0.63	9.69 ± 5.22	7.98 ± 3.33	4.22 ± 0.82

See Table 4 for the number of sampled probability sites and the estimated wetland area within NWCA ecoregion and wetland types on which population-weighted means are based.
