# Supplementary material for: Characterizing nonnative plants in wetlands across the conterminous United States
Source: Environ Monit Assess. 2019 Jun 20;191(Suppl 1):344. doi: 10.1007/s10661-019-7317-3 (PMC6586712; doi:10.1007/s10661-019-7317-3)
Supplement: Supplementary file 3 — (PDF 229 kb) [file 10661_2019_7317_MOESM3_ESM.pdf]

Supplement 3 – Partial dependence plots (Figs. A – O) from random forests classification analysis for Nonnative Plant Indicator (NNPI) stressor-levels

Journal: Environmental Monitoring and Assessment

Paper title: *Characterizing nonnative plants in wetlands across the conterminous United States*

Authors: Teresa K. Magee, Karen A. Blocksom, Alan T. Herlihy, and Amanda M. Nahlik

Corresponding Author: Teresa K. Magee, US Environmental Protection Agency, Office Research and Development, National Health Effects Laboratory, Western Ecology Division, Corvallis, Oregon

We conducted a random forests classification analysis (Liaw and Wiener 2015, R Core Team 2017) on the 1138 sampled sites from the 2011 National Wetland Condition Assessment to identify relationships between the nonnative plant indicator (NNPI) and a variety of potential predictor variable. NNPI was the response variable and we considered 15 predictor variables that described the native vegetation, the abiotic environment, human-mediated disturbance, ecoregion, and wetland type and at each sampled site. The NNPI is categorized into 4 stressor-levels (low, moderate, high, very high), but for this analysis the categories were collapsed into 2 stressor-levels (L-M = low and moderate, H-VH = high and very high). The *Methods* and *Results and Discussion* sections of the journal article describe predictor variables and details of the random forest analysis. (Predictor variables are also highlighted in Fig. 1 - ecoregions, Table 3 – wetland types, Table 7 – natural vegetation attributes and environmental characteristics, Fig. 6 – human-mediated disturbance metrics, and Fig. 7 – random forest variable importance plot).

Partial Dependence Plots of the predictor variables from the random forest analysis are provided in this appendix. The partial dependence plots were created using the partialPlot function in the randomForest package in R (Liaw and Wiener 2015), and depict the probability of the H-VH stressor-level for the Nonnative Plant Indicator (NNPI) as a function of a specific predictor variable after averaging out the effects of the other predictor variables in the model. The y-axis in plots represents one half the logit of the probability of the H-VH stressor-level (i.e., 0.5*log(p/(1-p), where p is the probability of H-VH). For the purposes of interpretation, this means that larger values on the y-axis indicate a higher probability of the H-VH status. The partial dependence plots for individual predictor variables (Figures A through M) are presented in descending order of variable importance (see Fig. 7 in the paper).

Liaw, A., & Wiener, M. (2015). 'randomForest': Breiman and Cutler's Random Forests for Classification and Regression. R package version 4.6-12. (https://CRAN.R-project.org/package=randomForest).

R Core Team (2017). R: A language and environment for statistical computing. R Foundation for Statistical Computing, Vienna, Austria (http://www.R-project.org/).

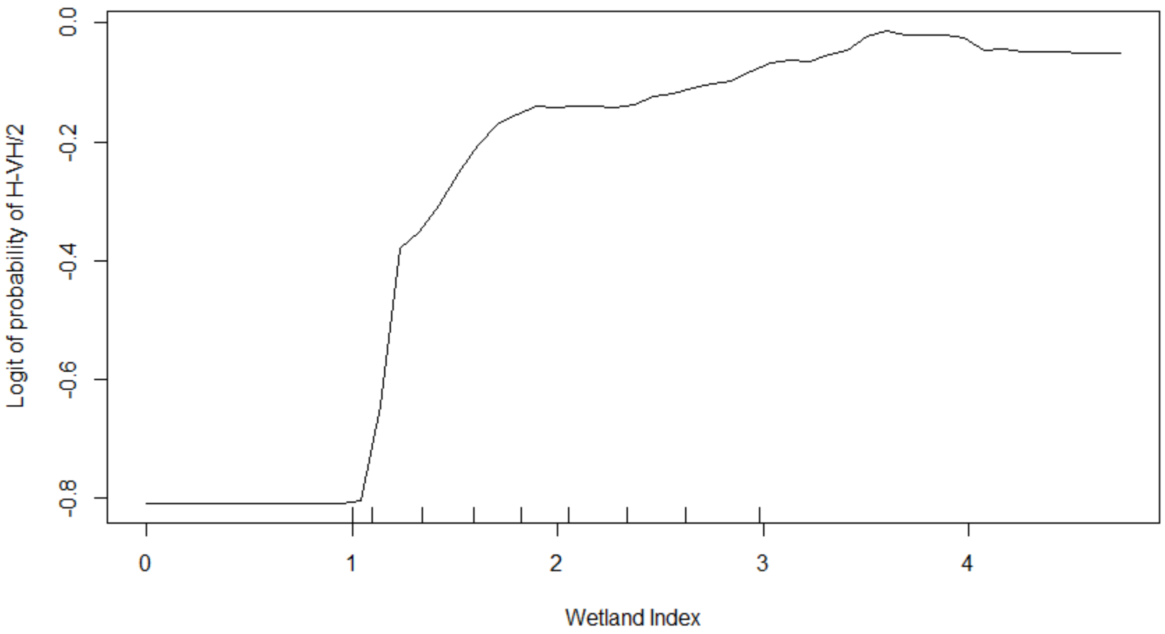

Fig. A Partial dependence plot for presence of H-VH NNPI stressor-level based on the Wetland Index (WI, range = 1 – 5). Larger y-axis values indicate higher probability of the H-VH status. WI = 1 indicates species composition (presence and abundance) is 100% obligate hydrophytes, WI = 5 indicates species composition is 100% upland taxa. In general, smaller WI indicates wetter conditions.

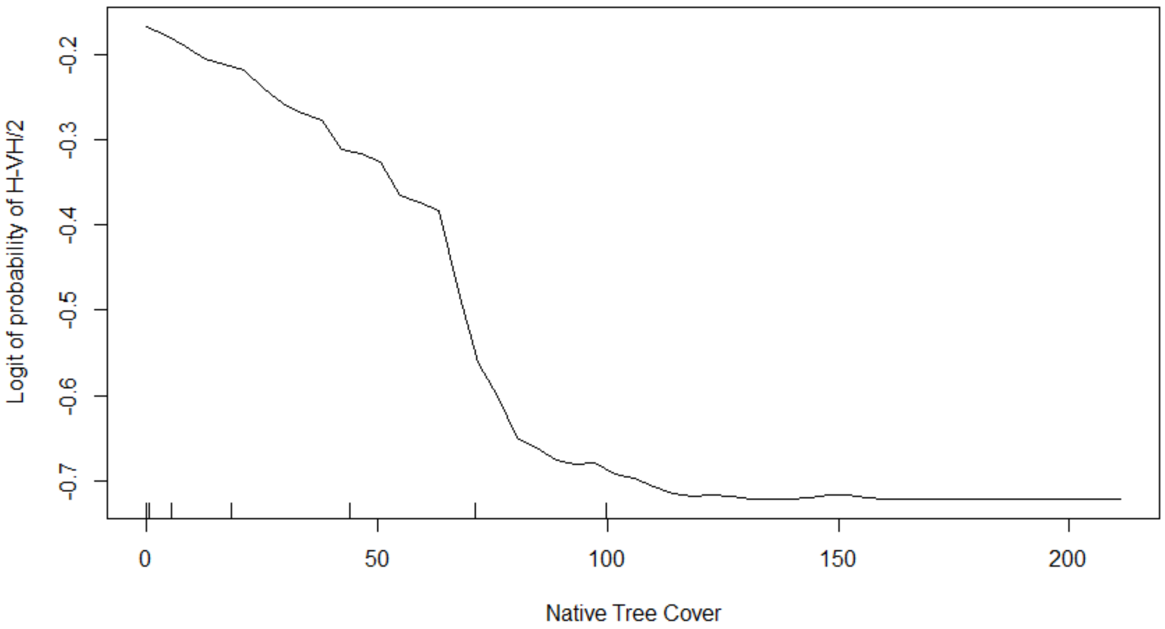

Fig. B Partial dependence plot for presence of H-VH NNPI stressor-level based on absolute (total) native tree cover. Larger y-axis values indicate higher probability of the H-VH status.

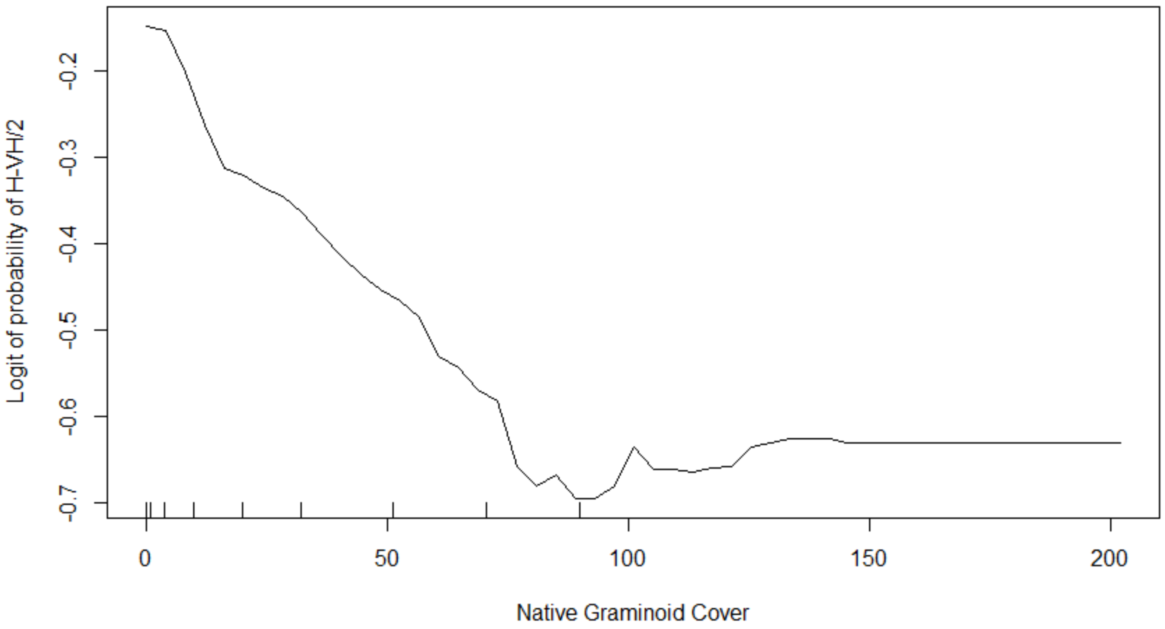

Fig. C Partial dependence plot for presence of H-VH NNPI stressor-level based on absolute (total) native graminoid cover. Larger y-axis values indicate higher probability of H-VH status.

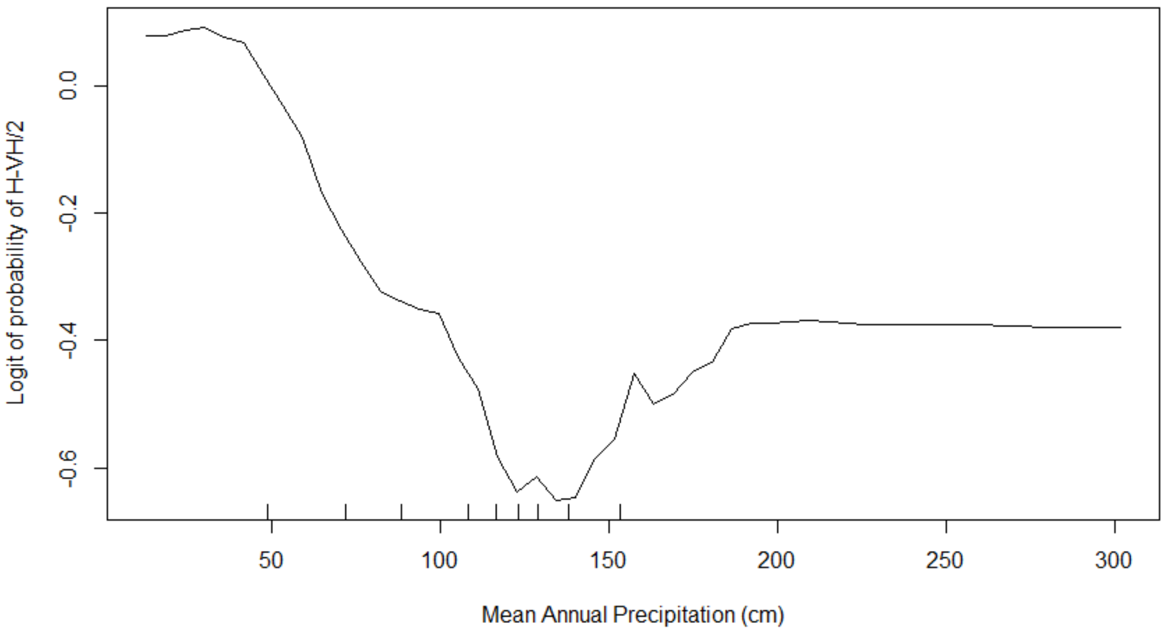

Fig. D Partial dependence plot for presence of H-VH NNPI stressor-level based on mean annual precipitation (centimeters). Larger y-axis values indicate higher probability of H-VH status.

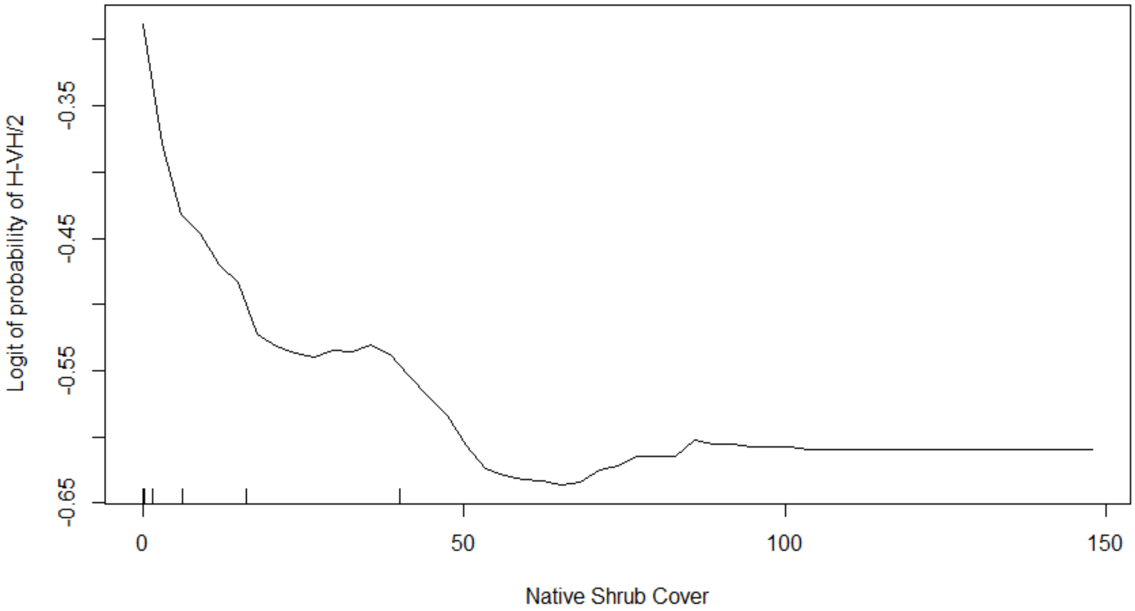

Fig. E Partial dependence plot for presence of H-VH NNPI stressor-level based on absolute (total) native shrub cover. Larger y-axis values indicate higher probability of H-VH status.

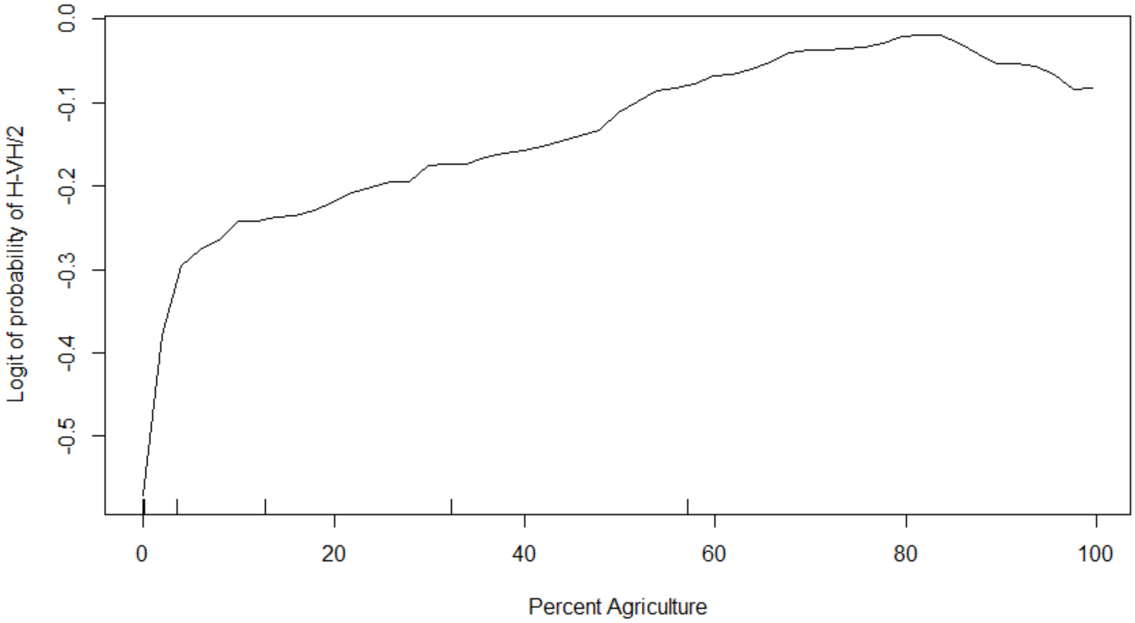

Fig. F Partial dependence plot for presence of H-VH NNPI stressor-level based on percent of area with agriculture land use in 1000m radius surrounding the center of a sampled site. Larger y-axis values indicate higher probability of H-VH status.

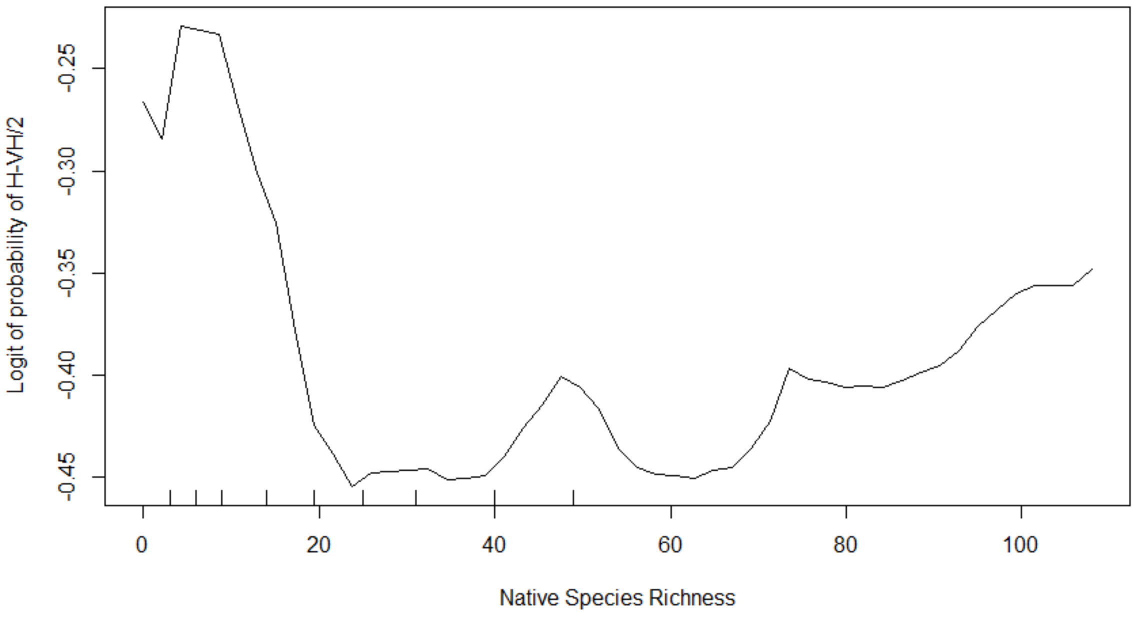

Fig. G Partial dependence plot for presence of H-VH NNPI stressor-level based on native species richness. Larger y-axis values indicate higher probability of H-VH status.

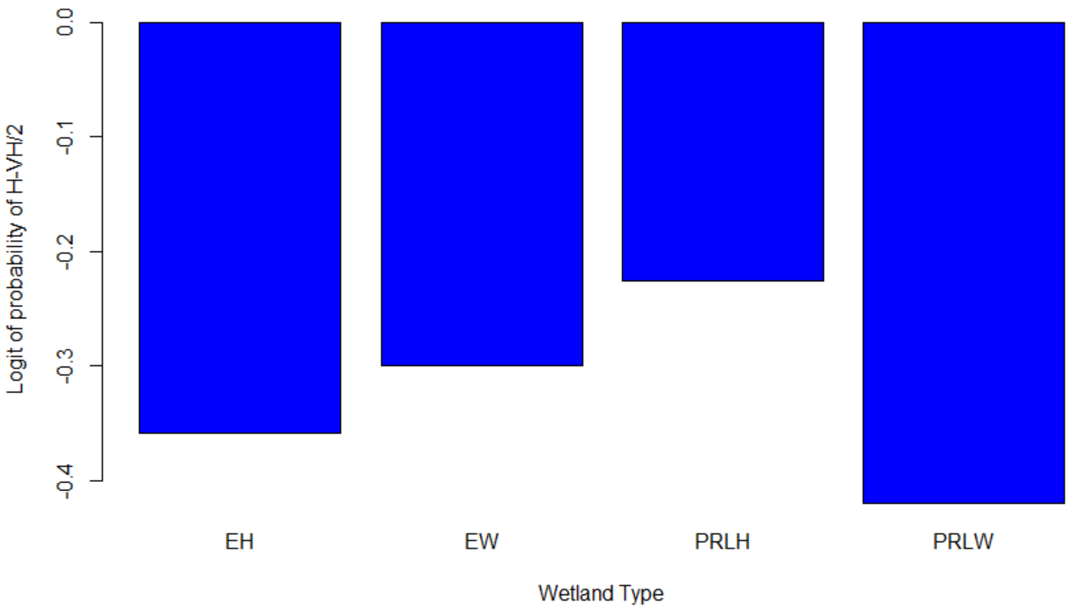

Fig. H Partial dependence plot for presence of H-VH NNPI stressor-level based on NWCA Aggregated Wetland Type (EH – Estuarine herbaceous, EW – Estuarine Woody, PRLH – Inland (palustrine, riverine, or lacustrine) herbaceous, PRLW – Inland Woody). Larger y-axis values indicate higher probability of H-VH status.

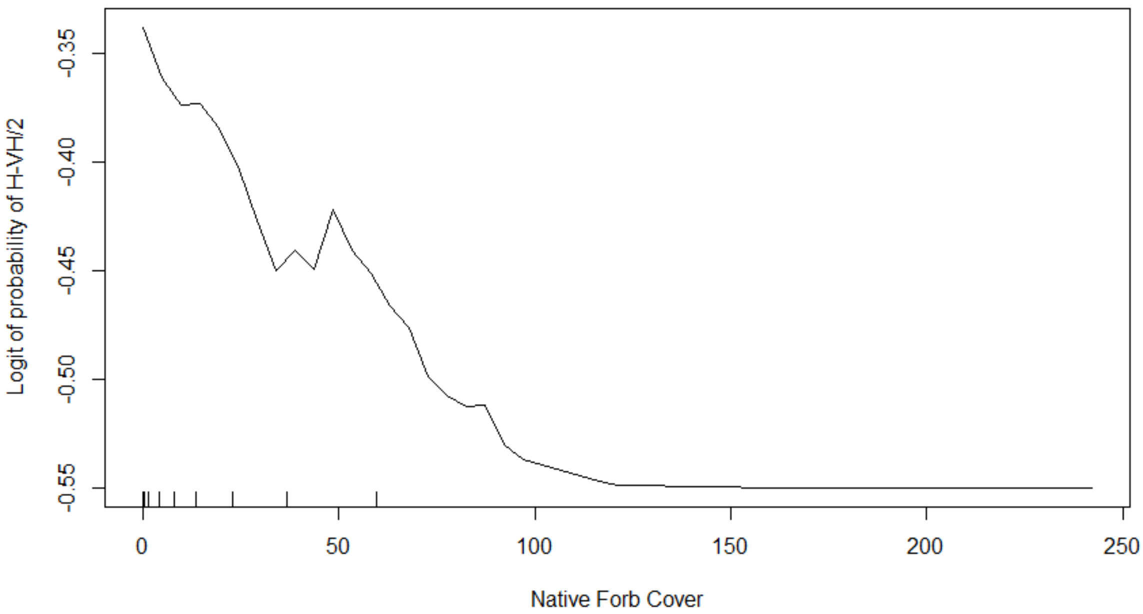

Fig. I Partial dependence plot for presence of H-VH NNPI stressor-level based on absolute (total) native forb cover. Larger y-axis values indicate higher probability of H-VH status.

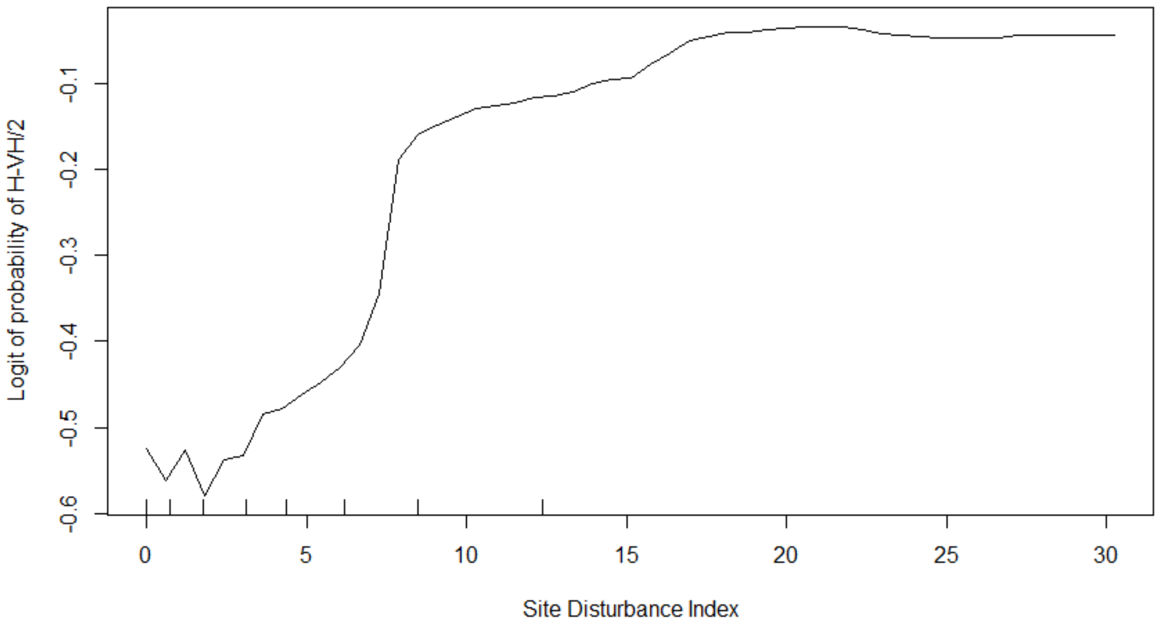

Fig. J Partial dependence plot for presence of H-VH NNPI stressor-level based on the Site Disturbance Index (SDI). Larger y-axis values indicate higher probability of H-VH status.

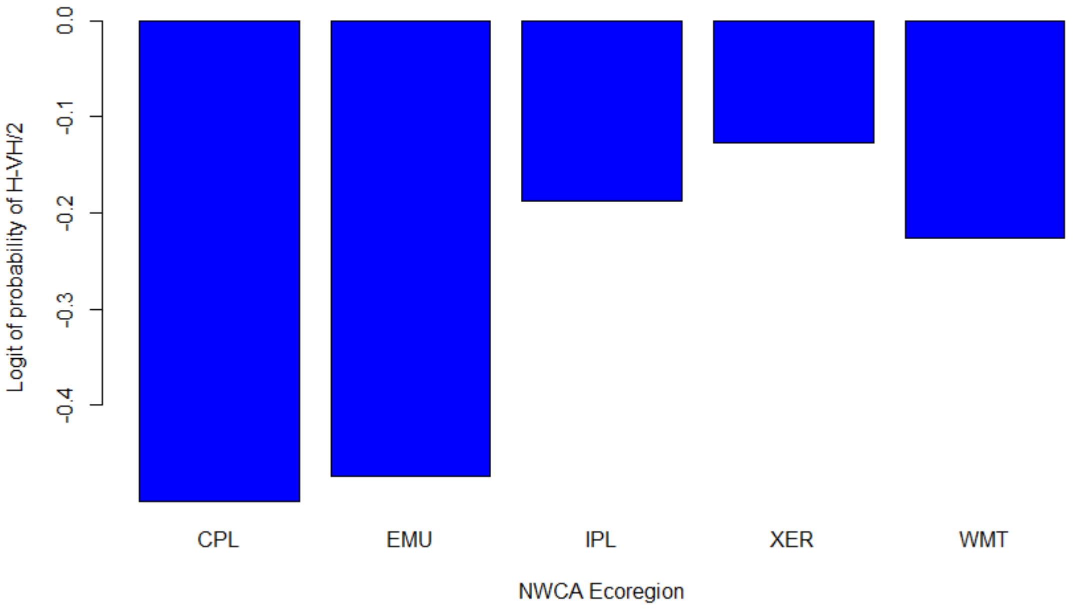

Fig. K Partial dependence plot for presence of H-VH NNPI stressor-level based on NWCA Aggregated Ecoregion (CPL – Coastal Plains, EMU – Eastern Mountains and Upper Midwest, IPL – Interior Plains, XER – Xeric West, WMT – Western Mountains and Valleys). Larger y-axis values indicate higher probability of H-VH status.

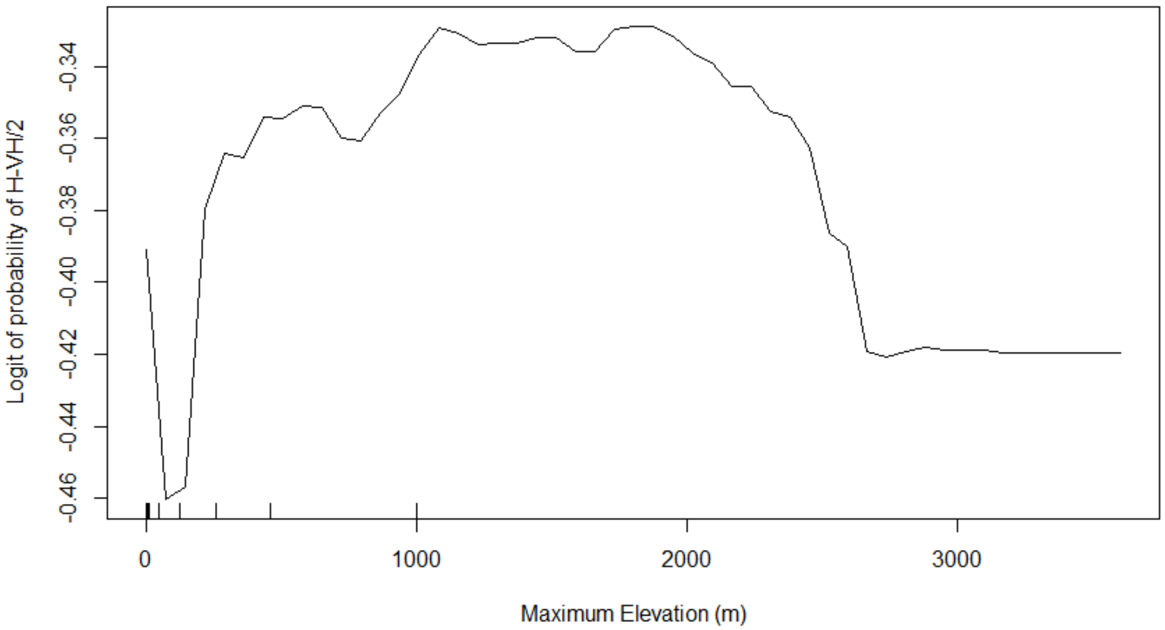

Fig. L Partial dependence plot for presence of H-VH NNPI stressor-level based on maximum elevation (meters) in the 200 m radius around the site center. Larger y-axis values indicate higher probability of H-VH status.

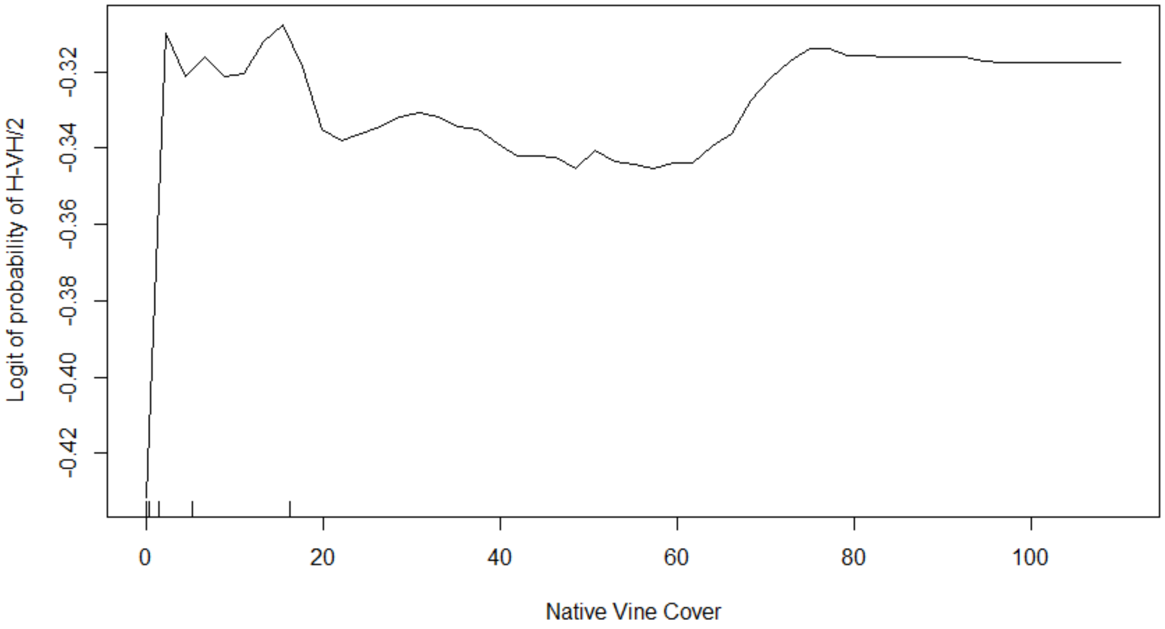

Fig. M Partial dependence plot for presence of H-VH NNPI stressor-level based on absolute (total) native vine. Larger y-axis values indicate higher probability of H-VH status.

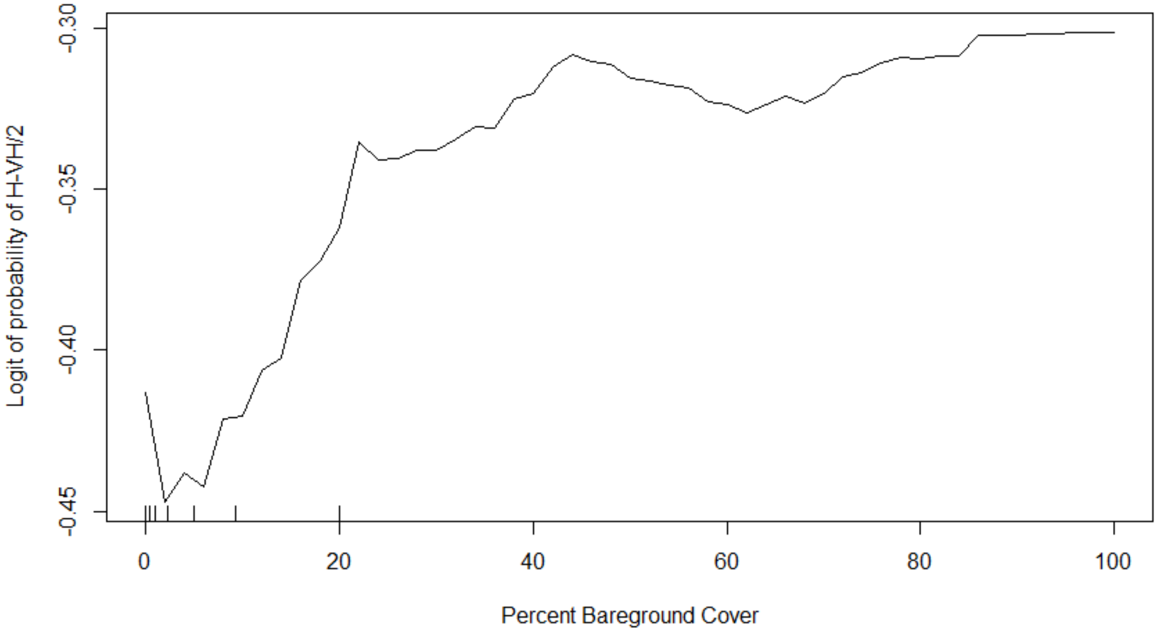

Fig. N Partial dependence plot for presence of H-VH NNPI stressor-level based on percent bareground cover. Larger y-axis values indicate higher probability of H-VH status.

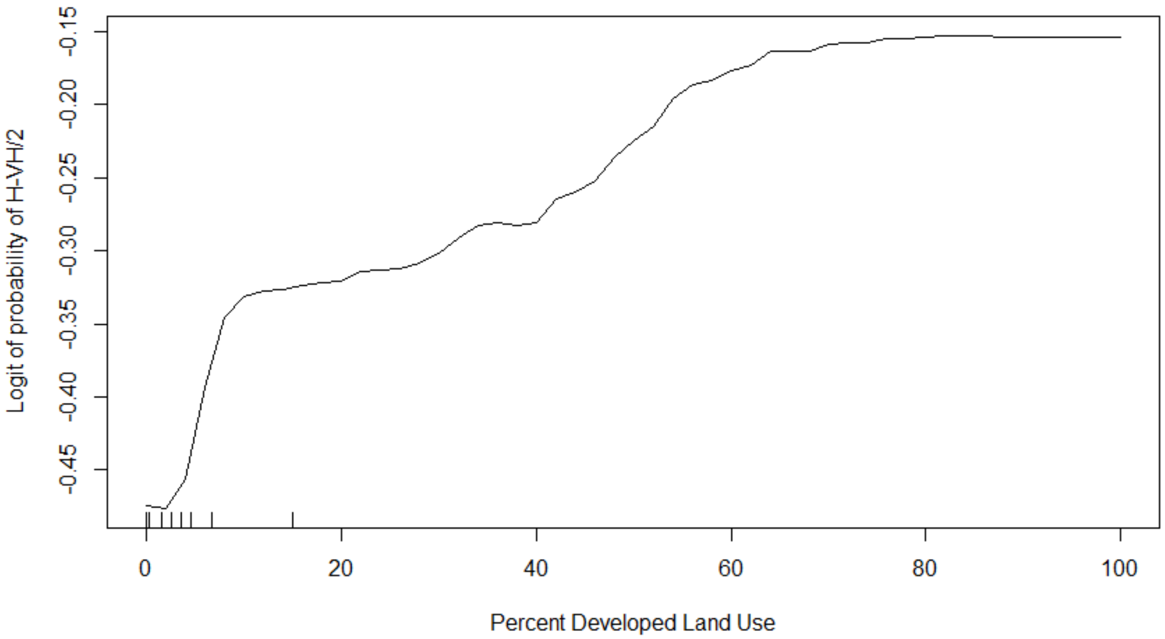

Fig. O Partial dependence plot for presence of H-VH NNPI stressor-level based on percent of area with developed land use in the 1000m radius surrounding the center of a sampled site. Larger y-axis values indicate higher probability of H-VH status.
